# HRD1-induced TMEM2 ubiquitination promotes ER stress-mediated apoptosis through a non-canonical pathway in intestinal ischemia/reperfusion

**DOI:** 10.1038/s41419-024-06504-0

**Published:** 2024-02-20

**Authors:** Xuzi Zhao, Deshun Liu, Yan Zhao, Zhecheng Wang, Yue Wang, Zhao Chen, Shili Ning, Guangzhi Wang, Lu Meng, Jihong Yao, Xiaofeng Tian

**Affiliations:** 1 Department of General Surgery, The Second Affiliated Hospital of Dalian Medical University, 116023 Dalian, China; 2 Department of Pharmacology, Dalian Medical University, 116044 Dalian, China

**Keywords:** Ubiquitin ligases, Gastrointestinal diseases

## Abstract

Intestinal ischemia/reperfusion (I/R) injury is a typical pathological course in the clinic with a high morbidity rate. Recent research has pointed out the critical role of ubiquitination during the occurrence and development of intestinal I/R by precisely mediating protein quality control and function. Here, we conducted an integrated multiomic analysis to identify critical ubiquitination-associated molecules in intestinal I/R and identified endoplasmic reticulum-located HRD1 as a candidate molecule. During intestinal I/R, excessive ER stress plays a central role by causing apoptotic pathway activation. In particular, we found that ER stress-mediated apoptosis was mitigated by HRD1 knockdown in intestinal I/R mice. Mechanistically, TMEM2 was identified as a new substrate of HRD1 in intestinal I/R by mass spectrometry analysis, which has a crucial role in attenuating apoptosis and promoting non-canonical ER stress resistance. A strong negative correlation was found between the protein levels of HRD1 and TMEM2 in human intestinal ischemia samples. Specifically, HRD1 interacted with the lysine 42 residue of TMEM2 and reduced its stabilization by K48-linked polyubiquitination. Furthermore, KEGG pathway analysis revealed that TMEM2 regulated ER stress-mediated apoptosis in association with the PI3k/Akt signaling pathway rather than canonical ER stress pathways. In summary, HRD1 regulates ER stress-mediated apoptosis through a non-canonical pathway by ubiquitinating TMEM2 and inhibiting PI3k/Akt activation during intestinal I/R. The current study shows that HRD1 is an intestinal I/R critical regulator and that targeting the HRD1/TMEM2 axis may be a promising therapeutic approach.

## Introduction

Intestinal ischemia/reperfusion (I/R) injury is a severe pathological process related to mucosal barrier damage and microbiota abnormalities. Intestinal I/R initially occurs in the intestine and eventually develops into multiple organ dysfunction syndrome [1, 2]. Some approaches to treating intestinal I/R have been identified, including ischemic pretreatment and antioxidant administration [3, 4]. Unfortunately, there are no specific treatments for intestinal I/R that are being used in the clinic. Thus, there is an urgency to explore the underlying mechanisms of intestinal I/R and identify potential molecular targets for its treatment.

The ubiquitin-proteasome system (UPS) is a critical regulator of proteolysis [5]. Ubiquitination exerts multiple biological effects by covalently attaching ubiquitin to lysine residues on a target substrate [6]. In particular, current research suggests that ubiquitination is essential for the development of I/R. The E3 ligase HUWE1 prevents ferroptosis in acute hepatic injury by regulating ubiquitination of the target protein [7]. In addition, preventing ubiquitinated BNIP3L degradation subsequently improves cardiac I/R injury [8]. Thus, it is essential to identify critical ubiquitination-associated regulators that can influence intestinal I/R. Through integrated multiomic analysis, we found aberrant expression of the E3 ligase HMG-CoA reductase degradation protein 1 (HRD1) in intestinal I/R.

ER-localized HRD1 is an important E3 ligase that controls protein stability by ligating ubiquitin to target substrates. The classic function of HRD1 is to ubiquitinate misfolded proteins in the ER-associated degradation (ERAD) process [9–11]. However, ERAD mainly occurs in the ischemic phase, which is an adaptive cellular response to counteract the destructive effects of ER stress. When the adaptive response fails or ER stress conditions are prolonged, apoptosis will follow in the reperfusion phase [12]. A recent study reported that in kidney I/R injury, abnormally elevated HRD1 exacerbated apoptosis by downregulating specific substrates through the ubiquitination pathway rather than degrading misfolded proteins [13]. Moreover, we found that HRD1 expression was significantly upregulated in intestinal I/R by integrated multiomic analysis. However, whether HRD1 plays a role in ER stress-mediated apoptosis in intestinal I/R and its specific mechanism remains unclear.

Using coimmunoprecipitation (Co-IP)/MS, we identified transmembrane protein 2 (TMEM2) as a novel substrate of HRD1 in intestinal I/R. TMEM2 was originally identified as a classic hyaluronidase [14]. Recent studies have shown that TMEM2 can avoid the adverse impacts of ER stress rather than changing the early protective phase of the ER response [15]. Notably, TMEM2 regulates ER stress by decomposing HMW-HA to LMW-HA, which is independent of canonical UPR^ER^ activation. Non-canonical ER stress programs gradually influence cellular processes in combination with the UPR. In heart failure, AGGF1 regulates ER stress via a noncanonical manner to block cardiomyocyte apoptosis [16]. Therefore, we speculated that HRD1 may regulate ER stress-mediated apoptosis via a non-canonical pathway by ubiquitinating TMEM2 in intestinal I/R.

Our study confirmed that the expression of the E3 ligase HRD1 was positively correlated with intestinal I/R injury in patients and mice. Mechanistically, HRD1 could catalyze K48-linked polyubiquitination and proteasomal degradation of TMEM2 via ubiquitination of the lysine 42 residue, which in turn affected ER stress-mediated apoptosis via a non-canonical pathway. The results provide new perspectives into the role of HRD1 and suggest that focusing on the HRD1/TMEM2 axis may offer an innovative therapeutic strategy for intestinal I/R.

## Results

### HRD1 knockdown protects against ER stress-mediated apoptosis in intestinal I/R

**Fig. 1:**
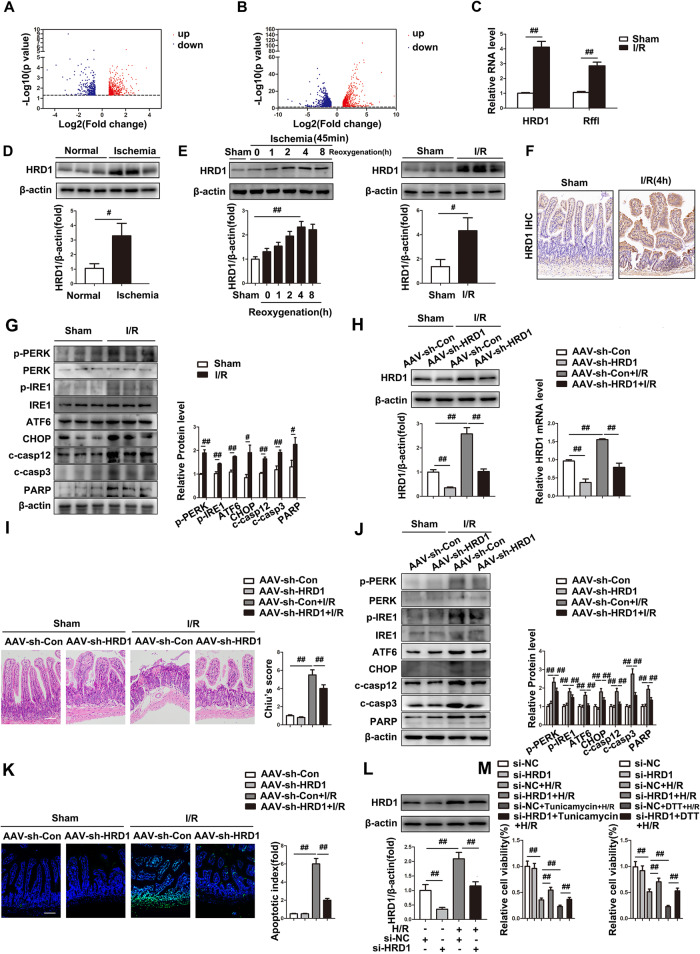
HRD1 knockdown protects against ER stress-mediated apoptosis in intestinal I/R. **A** Volcano plots of differentially expressed genes (DEGs) in the intestinal I/R and sham groups. **B** Volcano plots of DEGs in Caco-2 cells treated with H/R and the control. **C** Expression of the 2 selected RNAs (*n* = 6). **D** The expression of HRD1 in clinical patients with intestinal ischemia (*n* = 3). **E** The expression of HRD1 after hypoxia followed by reoxygenation for 0–8 h or normoxia (control) (*n* = 3). **F** HRD1 IHC staining (Scale bar = 100 µm). **G** Protein expressions (*n* = 3). **H**–**K** AAV-sh-HRD1 or AAV empty vectors were injected into C57BL/6 mice for three weeks before intestinal I/R challenge (*n* = 6). **H** HRD1 protein and mRNA expression. **I** H&E staining (Scale bar = 100 µm). **J** Protein expressions (*n* = 3). **K** TUNEL staining (Scale bar = 100 µm). **L**, **M** Caco-2 cells were transfected with si-HRD1 or si-NC before H/R challenge (*n* = 3). **L** HRD1 protein expressions (*n* = 3). **M** Relative cell viability was measured through CTG analysis (*n* = 6). ^#^*P* < 0.05, ^##^*P* < 0.01.

To identify aberrant ubiquitination-associated molecules in intestinal I/R, we used two orthogonal independent experiments (RNA-seq) (Fig. 1A, B). The E3 ligases HRD1 and Rffl were identified from two datasets, among which HRD1 exhibited the greatest alteration in expression (Fig. 1C). Initially, we demonstrated that intestine samples of patients with intestinal ischemia had a significantly elevated HRD1 protein level (Fig. 1D). Similar to human I/R injury, HRD1 protein expression was evaluated in I/R mice and peaked at 4 h after reperfusion (Fig. 1E). IHC staining supported this finding (Fig. 1F). Furthermore, an increase in ER stress-mediated apoptosis was detected in I/R intestines (Fig. 1G). These findings demonstrated a positive correlation between intestinal I/R severity and HRD1 expression.

Next, we generated HRD1-knockdown mice with AAV-shRNA-HRD1 to examine how HRD1 functions in intestinal I/R (Fig. 1H). As expected, HRD1 knockdown improved intestinal and remote organ injury, as evidenced by H&E staining, and decreased the serum levels of alanine transaminase (ALT), aspartate aminotransferase (AST) and myeloperoxidase (MPO) (Fig. 1I, S1). More strikingly, HRD1-knockdown mice exhibited reduced expression of ER stress-mediated apoptotic markers (p-PERK, p-IRE1, ATF6, CHOP, cleaved caspase12, cleaved caspase3, and cleaved PARP) after intestinal I/R (Fig. 1J). Similarly, the number of TUNEL-positive apoptotic cells was notably decreased in HRD1 shRNA-injected mice (Fig. 1K). Furthermore, HRD1 silencing enhanced ER stress resistance [17] and decreased cleaved caspase-3 activity under H/R conditions (Fig. 1L-M, S2A). In contrast, ER stress-mediated apoptosis during intestinal I/R was exacerbated by HRD1 overexpression in vitro (Fig. S2B–E). Based on these results, HRD1 is pivotal for ER stress-mediated apoptosis in intestinal I/R.

### HRD1 interacts with TMEM2 and promotes its ubiquitination

**Fig. 2:**
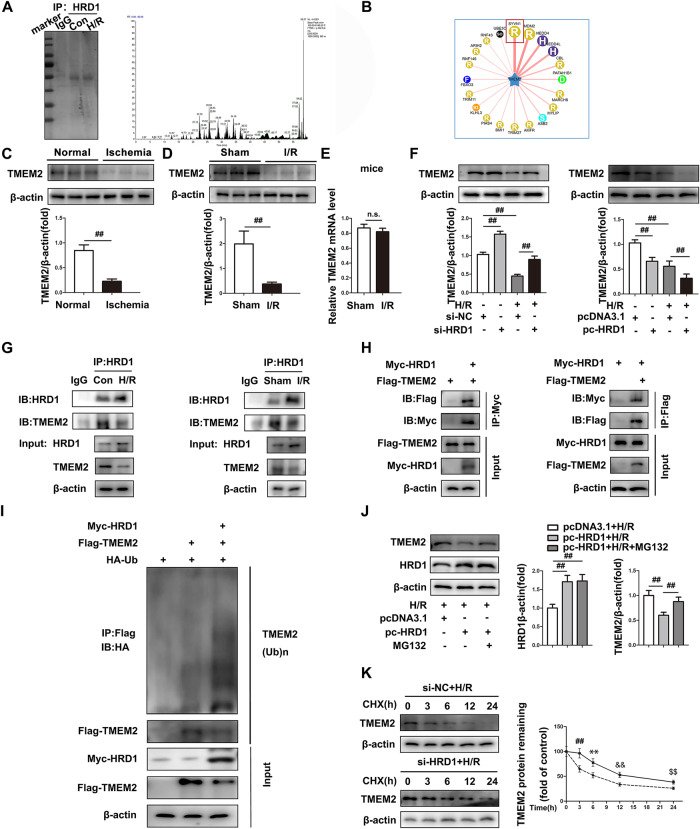
HRD1 interacts with TMEM2 and promotes its ubiquitination. **A** Detection of HRD1-specific binding proteins by IP-MS. **B** The network view of the predicted E3 ligase of TMEM2 by UbiBrowser. **C** The expression of TMEM2 in clinical patients with intestinal ischemia (*n* = 3). **D** TMEM2 protein expressions (*n* = 3). **E** TMEM2 mRNA levels (*n* = 6). **F** Assessment of TMEM2 expression after transfection of si-HRD1 or pc-HRD1 into Caco-2 cells (*n* = 3). **G** The interaction between endogenous TMEM2 and HRD1 in Caco-2 cells. **H** The interaction between ectopic Flag-TMEM2 and Myc-HRD1 in HEK293 cells. **I** An IP assay was used to examine TMEM2 ubiquitination. **J** MG132 treatment was administered after pc-HRD1 transfection of Caco-2 cells. Evaluation of TMEM2 expression (*n* = 3). **K** Before CHX treatment at the specified times, si-HRD1 was transfected into Caco-2 cells. Evaluation of TMEM2 expression (*n* = 3). ^**^P < 0.01, ^##^*P* < 0.01, ^&&^*P* < 0.01, ^$$^*P* < 0.01.

As a ubiquitin ligase, HRD1 typically interacts with a target protein and degrades it via the ubiquitination pathway [18]. To clarify the molecular mechanism of HRD1 in response to ER stress-mediated apoptosis in intestinal I/R, we investigated HRD1 binding proteins by mass spectrometric analysis. Coomassie blue staining was used to distinguish proteins that interacted with HRD1 following IP with anti-HRD1 antibody in Caco-2 cells (Fig. 2A). Figure 2A shows that 58 proteins were potential substrates of HRD1 in intestinal I/R. Surprisingly, among these proteins, TMEM2 has the ability to avoid ER stress-mediated apoptosis via a non-canonical pathway [15]. Additionally, HRD1 was identified as a candidate E3 ligase that could ubiquitinate TMEM2, as shown by UbiBrowser software [19] (Fig. 2B). Furthermore, TMEM2 protein expression was observably decreased after intestinal I/R (Fig. 2C, D, S3A). However, TMEM2 mRNA changed slightly both in vivo and vitro, highlighting the importance of posttranslational modifications (PTMs) in regulating TMEM2 expression under intestinal I/R conditions (Fig. 2E, S3B).

Then, we focused on the interaction between HRD1 and TMEM2. TMEM2 protein expression was markedly increased by HRD1 silencing and decreased by HRD1 overexpression (Fig. 2F). Moreover, there were no discernible changes in TMEM2 mRNA with HRD1 overexpression or knockdown (Fig. S3C). During I/R or H/R, HRD1 could interact with TMEM2 (Fig. 2G). The interaction of ectopic FLAG-TMEM2 with Myc-HRD1 was shown by Co-IP in HEK293 cells (Fig. 2H). Having determined the interaction of HRD1 and TMEM2, we next investigated whether HRD1 catalyzed TMEM2 ubiquitination. As shown in Fig. 2I, the transfection of Myc-HRD1 in HEK293 cells increased the level of ubiquitination of TMEM2. Additionally, the proteasome-specific inhibitor MG132 prevented TMEM2 degradation caused by pc-HRD1 (Fig. 2J). Following CHX exposure, TMEM2 protein expression decreased with time, while HRD1 knockdown prolonged the half-life of TMEM2 (Fig. 2K). These results suggest that the HRD1-induced reduction in TMEM2 protein levels is ubiquitination dependent.

### HRD1 targets the TMEM2 lysine 42 residue for K48-linked polyubiquitination

**Fig. 3:**
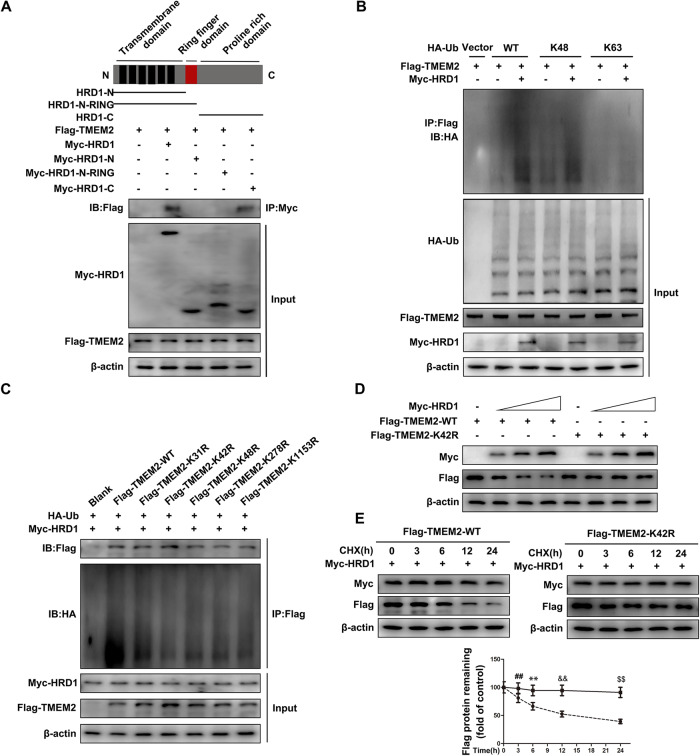
HRD1 targets the TMEM2 lysine 42 residue for K48-linked polyubiquitination. **A** The specific domain of HRD1 that binds to TMEM2 was detected by Co-IP. **B** IP analysis of lysates from HEK293 cells cotransfected with K48-Ub or K63-Ub mutant, Flag-TMEM2, and Myc-HRD1. **C** Co-IP detection of TMEM2-specific binding sites to HRD1. **D** Lysates from HEK293 cells cotransfected with Flag-TMEM2, Flag-TMEM2-K42R, and Myc-HRD1 (0, 0.5, 1 or 2 μg) expression plasmids were subjected to immunoblot analysis. **E** Before CHX treatment at the specified times, Flag-TMEM2 or Flag-TMEM2-k42R and Myc-HRD1 were cotransfected into HEK293 cells. Evaluation of Flag-TMEM2 expression (*n* = 3). ^**^P < 0.01, ^##^*P* < 0.01, ^&&^*P* < 0.01, ^$$^*P* < 0.01.

To explore the domain of HRD1 that is associated with the TMEM2 interaction, numerous deletion mutations of Myc-HRD1 were produced. As expected, the removal of the COOH-terminal domain completely eliminated the connection between TMEM2 and HRD1, providing more evidence that HRD1 interacts with TMEM2 through its cytosolic COOH-terminal domain (Fig. 3A). Subsequently, to determine the polyubiquitin chain linkage on TMEM2 catalyzed by HRD1, we designed HA-Ub ubiquitin mutants. K48 and K63 residues on the ubiquitin chain were preserved, while the remaining lysine residues were substituted with arginine. HRD1 significantly enhanced K48-linked polyubiquitination of TMEM2 but not K63-linked polyubiquitination (Fig. 3B). These data validated HRD1 as an E3 ligase that directly initiates the K48-linked polyubiquitination of TMEM2.

Given that ubiquitination typically takes place on lysine residues in protein substrates, we next used the Biogrid database (https://thebiogrid.org) [20] to predict that five lysine sites were present in TMEM2. Five lysine residues on TMEM2 were mutated to arginine and cotransfected into HEK293 cells with HA-Ub. The findings revealed that the level of ubiquitination was greatly reduced after the TMEM2 lysine 42 residue was mutated (Fig. 3C). The Flag-TMEM2 decrease caused by Myc-HRD1 overexpression was restored after the lysine 42 residue was changed to arginine (Fig. 3D). Similarly, the protein stability of TMEM2 with the lysine 42 mutation was not affected by HRD1 expression (Fig. 3E). These results suggest that HRD1 ubiquitinates and degrades TMEM2 mainly through the lysine 42 residue.

### TMEM2 is required for resistance to ER-mediated apoptosis in intestinal I/R

**Fig. 4:**
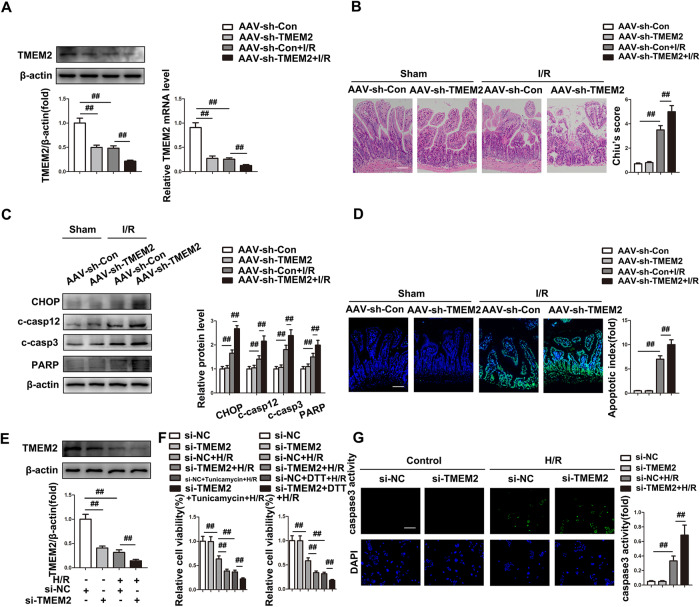
TMEM2 is required for resistance to ER-mediated apoptosis in intestinal I/R. **A**–**D** AAV-sh-TMEM2 or AAV empty vectors were injected into C57BL/6 mice for three weeks before intestinal I/R challenge (*n* = 6). **A** TMEM2 protein and mRNA expression. **B** H&E staining (Scale bar = 100 µm). **C** Protein expressions (*n* = 3). **D** TUNEL staining (Scale bar = 100 µm). **E**–**G** Caco-2 cells were transfected with si-TMEM2 or si-NC before the H/R challenge (*n* = 3). **E** TMEM2 protein expressions (*n* = 3). **F** Relative cell viability was measured through CTG analysis (*n* = 6). **G** Caspase-3 activity (Scale bar = 100 µm). ^##^*P* < 0.01.

Because TMEM2 plays an indispensable role in avoiding the deleterious consequences of ER stress, we investigated TMEM2 functions in intestinal I/R and generated TMEM2-knockdown mice with AAV-shRNA-TMEM2 (Fig. 4A). TMEM2 silencing markedly promoted intestinal injury, as evidenced by intestinal histopathology (Fig. 4B). Recent research has shown that decreased ER stress resistance caused by TMEM2 knockdown is unaffected by canonical ER stress pathway inhibitors [15]. As expected, the lack of TMEM2 exerted no discernible effects on the gene expression of ER stress canonical pathways (PERK1, ATF6, or IRE1) under H/R challenge, as shown by western blotting (Fig. S4). Notably, increased ER stress-mediated apoptotic markers (CHOP, cleaved caspase12, cleaved caspase3, and cleaved PARP) were discovered after TMEM2 knockdown in intestinal I/R (Fig. 4C). The number of TUNEL-positive apoptotic cells was markedly elevated in TMEM2 shRNA-injected mice (Fig. 4D). Furthermore, TMEM2 knockdown attenuated ER stress resistance and increased cleaved caspase-3 activity under H/R conditions (Fig. 4E–G). In contrast, ER stress-mediated apoptosis during intestinal I/R was reversed by TMEM2 overexpression in vitro (Fig. S5). Collectively, TMEM2 plays an important role in ER stress-mediated apoptosis in intestinal I/R.

### TMEM2 suppresses ER stress-mediated apoptosis by regulating the PI3k-Akt signaling pathway

**Fig. 5:**
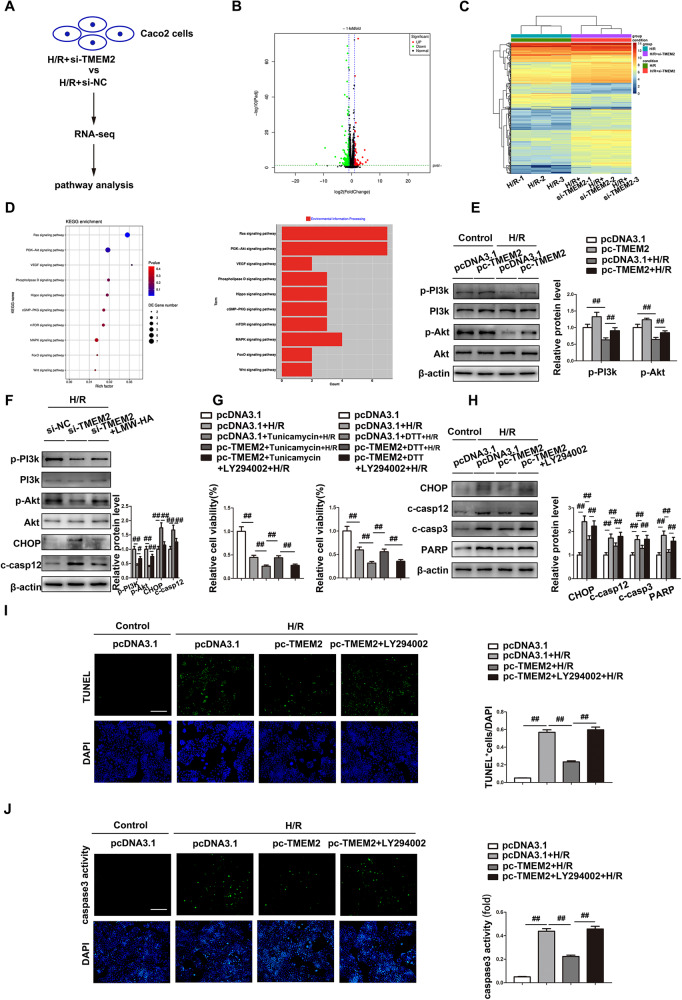
TMEM2 suppresses ER stress-mediated apoptosis by regulating the PI3k-Akt signaling pathway. **A** Scheme showing the procedure for identifying the signaling pathways regulated by TMEM2 in H/R. **B** A volcano plot showing DEGs (red, upregulated genes; green, downregulated genes) in Caco-2 cells transfected with si-NC or si-TMEM2 and treated with H/R. **C** Heatmap showing differential gene expression between the H/R and H/R+si-TMEM2 groups. **D** KEGG analysis of the RNA-seq data. **E** p-PI3k and p-Akt protein expressions (*n* = 3). **F** Caco-2 cells were transfected with si-TMEM2 and then exposed to 100 μg/ml LMWHA for 24 h and H/R (*n* = 3). Protein expressions (*n* = 3). **G** Relative cell viability was measured through CTG analysis (*n* = 6). **H**–**J** Caco-2 cells were transfected with pc-TMEM2 and then exposed to LY294002 (10 μmol/L) for 8 h and H/R (*n* = 3). **H** Protein expressions (*n* = 3). **I** TUNEL staining (Scale bar = 100 µm). **J** Caspase-3 activity (Scale bar =100 µm). ^##^*P* < 0.01.

To investigate the mechanism of the protective function of TMEM2 in response to ER stress-mediated apoptosis in intestinal I/R, we performed RNA-seq analysis between the H/R and H/R+si-TMEM2 groups (Fig. 5A). Figure 5B, C shows differentially expressed genes (fold change ≥2 and *P* < 0.05) (Supplementary Material 3). Furthermore, KEGG transcriptomics analysis identified 10 pathways regulated by TMEM2 associated with ER stress. Previous work has found that PI3k-Akt pathway components are critical in cell fate decisions when ER stress is present [21, 22]. In particular, the PI3k-Akt pathway exhibited a high enrichment score in KEGG analysis, while three canonical ER stress pathways (PERK1, ATF6, or IRE1) were not included (Fig. 5D, Supplementary Material 4). As shown in Fig. 5E, TMEM2 overexpression increased PI3k and Akt activation after H/R in Caco-2 cells. Moreover, the HAase activity of TMEM2 is responsible for the breakdown of HMW-HA to LMW-HA [23]. To determine whether TMEM2 activates PI3k/Akt by increasing LMW-HA, LMW-HA was coupled with TMEM2 silencing under H/R conditions, and the results showed that LMW-HA activated PI3k/Akt and inhibited ER stress-mediated apoptosis (Fig. 5F).

We further assessed whether TMEM2 plays a role in I/R injury by activating PI3k/Akt. Although TMEM2 overexpression resulted in a substantial decrease in ER stress-mediated apoptosis (Fig. S5), the PI3k-Akt inhibitor LY294002 reversed these effects. LY294002 attenuated ER stress resistance in the pc-TMEM2 group (Fig. 5G). Furthermore, the decreased ER stress-mediated apoptotic markers (CHOP, cleaved caspase12, cleaved caspase3, and cleaved PARP) were elevated by LY294002 in the TMEM2 overexpression group (Fig. 5H). Similarly, LY294002 attenuated the protection against H/R injury conferred by TMEM2 overexpression (Fig. 5I, J). Thus, these findings imply that TMEM2 attenuates ER stress-mediated apoptosis by triggering the PI3k-Akt pathway in intestinal I/R.

### Inhibition of TMEM2 abolishes the protective effect of HRD1 deficiency in intestinal I/R

**Fig. 6:**
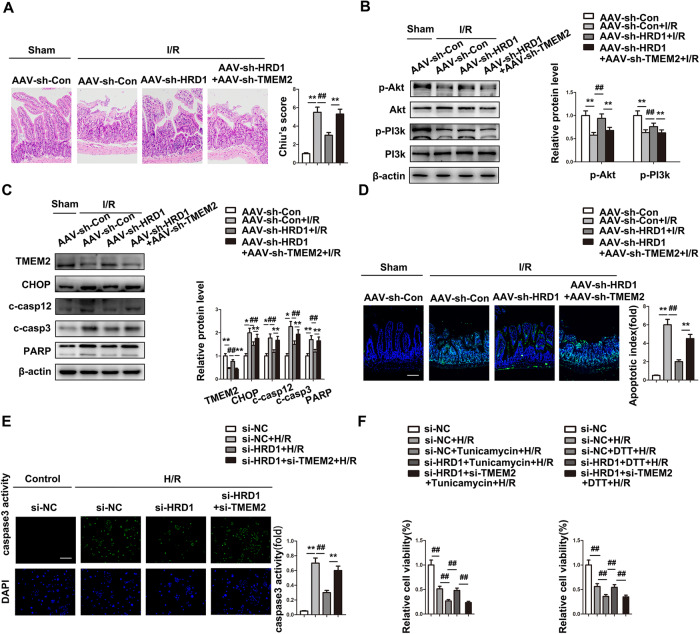
Inhibition of TMEM2 abolishes the protective effect of HRD1 deficiency in intestinal I/R. **A**–**D** AAV-sh-HRD1, AAV-sh-TMEM2 or AAV empty vectors were coinjected into C57BL/6 mice for three weeks before intestinal I/R challenge (*n* = 6). **A** H&E staining (Scale bar=100 µm). **B** p-PI3k and p-Akt protein expressions (*n* = 3). **C** Protein expressions (*n* = 3). **D** TUNEL staining (Scale bar = 100 µm). **E** Caspase-3 activity (Scale bar = 100 µm). **F** Relative cell viability was measured through CTG analysis (*n* = 6). ^#^*P* < 0.05, ^##^*P* < 0.01.

To confirm that the effect of HRD1 on intestinal I/R relies on modulating TMEM2 expression, TMEM2 and HRD1 were simultaneously knocked down under I/R conditions. The extent of intestinal histopathology was mitigated in HRD1 shRNA-injected mice, and this effect was blocked by TMEM2 knockdown (Fig. 6A). Accordingly, PI3k and Akt activation caused by HRD1 silencing were disrupted by reducing TMEM2 expression (Fig. 6B). Furthermore, the protective effect of HRD1 knockdown against ER stress-mediated apoptosis was reversed by TMEM2 deficiency (Fig. 6C–F). Collectively, these data show that the injurious effects of HRD1 on intestinal I/R are at least partly reliant on the reduction in TMEM2.

## Discussion

Intestinal I/R injury is a serious pathological process that can lead to clinically significant events that eventually progress to multiple organ dysfunction syndrome (MODS) [24, 25]. Detrimental consequences of ER stress are key in the pathogenesis of intestinal I/R [26, 27]. This investigation demonstrated several findings. (1) HRD1 silencing protects against ER stress-mediated apoptosis in intestinal I/R. (2) HRD1 interacts with TMEM2 and degrades TMEM2 through the ubiquitin‒proteasome pathway. (3) The interaction of HRD1 and TMEM2 controls ER stress-mediated apoptosis via a non-canonical pathway during intestinal I/R.

Ubiquitination affects various aspects of cellular processes, including transcriptional regulation, cell proliferation, DNA damage and signal transmission [28, 29]. Importantly, the activity and expression of ubiquitin enzymes aberrantly change with the induction of various cellular stresses [30–32], which is presumed to possess a significant impact on I/R damage across numerous organs. Elucidating the specific mechanism of the ubiquitin enzyme in intestinal I/R is expected to provide an alternative therapeutic strategy. To identify crucial ubiquitination-associated molecules that affect intestinal I/R, we performed an integrated multiomic analysis and found that E3 ubiquitin ligase HRD1 exhibited the greatest expression alteration.

HRD1 is crucial for the ubiquitination and dislocation of misfolded ERAD proteins under physiological conditions [33]. The ERAD process is mainly characterized as a protective response during the ischemic process. However, if the injury is excessive during the reperfusion process, numerous elements, including ROS, intracellular Ca2^+^ overload and inflammation, disrupt the normal function of the ER and lead to the induction of the proapoptotic signaling pathway [34]. Moreover, the current study emphasized the crucial role of ER stress-mediated apoptosis in regulating the pathogenesis of I/R. RTN1-C knockdown reduced the levels of ER stress-mediated apoptotic molecules, which alleviated cerebral I/R damage [35]. Similar to what was observed in acute kidney injury [13], HRD1 expression in the current study had a positive correlation with the degree of intestinal damage. A lack of HRD1 markedly relieved ER stress-mediated apoptosis, as indicated by the decreased expression of proapoptotic ER markers. These findings show that HRD1 upregulation is adequate to induce ER stress-mediated apoptosis, which promotes intestinal I/R injury.

The molecular mechanisms driving the ubiquitination of HRD1 are mostly connected to its substrates [36, 37]. We found that the ER stress-associated factor TMEM2 was a potential substrate of HRD1 through mass spectrometry. TMEM2 has been shown to be crucial for avoiding detrimental ER stress-mediated consequences, as indicated by reducing apoptosis or cellular senescence rather than enhancing the initial protective phase of the ER stress response [17]. The clinical negative correlation between HRD1 and TMEM2 was identified in patients suffering from intestinal ischemia, demonstrating a potential interaction between HRD1 and TMEM2. Moreover, HRD1 overexpression increased TMEM2 ubiquitination, leading to decreased TMEM2 protein expression, which was responsible for aberrant TMEM2 degradation after intestinal I/R. HRD1 is composed of a transmembrane domain, a RING-finger domain, and a proline-rich C-terminal domain [38]. Interestingly, we found that HRD1 directly bound to TMEM2 through the cytosolic C-terminal domain. In addition, the ubiquitinating lysine sites in TMEM2 have not been identified. After the lysine 42 residue on TMEM2 was mutated, HRD1 was unable to influence the expression of TMEM2. Thus, our findings show that HRD1 is involved in TMEM2 ubiquitination, indicating that the interaction between HRD1 and TMEM2 is important for ER stress-mediated apoptosis.

Recent research has demonstrated that TMEM2 alters ER stress by breaking down glycosaminoglycan and hyaluronan, which is a non-canonical ER stress manner [15]. It has been documented that non-canonical ER stress responses are essential for regulating cell fate through a variety of mechanisms [39, 40]. For instance, in mutant melanoma with BRAF inhibitor treatment, Grp78 binds the scaffold protein KSR2 to form a multiprotein complex, which acts independently of the UPR to activate ERK and promote the phosphorylation of ATF4 [41]. Notably, we found that TMEM2 was unable to influence the canonical UPR pathways of PERK, ATF6, and IRE1 in intestinal I/R by RNA-seq analysis. Furthermore, PERK, ATF6, and IRE1 expression were not changed by TMEM2 silencing. Additionally, pharmacological inhibition of the canonical ER stress pathway had no effect on the decrease in ER stress sensitivity when TMEM2 knockdown [15]. Notably, the PI3k/Akt pathway was an underlying downstream target of TMEM2 in intestinal I/R. The PI3k/Akt pathway components are crucial in determining cell fate when ER stress occurs [42, 43]. Moreover, PI3k-Akt inhibition caused the levels of CHOP and cleaved caspase12 to increase after TMEM2 overexpression. Based on this information, we concluded that TMEM2 regulates ER stress via a non-canonical pathway during intestinal I/R. However, whether TMEM2 activates the PI3k/Akt and is associated with recruiting different HA-related receptors needs further study.

Our findings indicate that HRD1 can interact with TMEM2, a key component of the non-canonical ER stress pathway, to modulate ER signaling. HRD1 ubiquitinates and reduces the expression of TMEM2, which then negatively regulates the activation of PI3k/Akt by inhibiting HA breakdown, thereby promoting ER stress-mediated apoptosis. Additionally, HRD1 can modulate classical ER pathway markers, such as p-PERK, p-IRE1, and ATF6, in intestinal I/R injury. The classical ER stress response protein XBP1 participates in the crosstalk between ER stress and mitochondrial dysfunction by upregulating HRD1 expression, consequently contributing to the development of acute kidney injury [44]. Therefore, HRD1 may be involved in other molecular processes in addition to the inhibition of TMEM2, which can impact the classical ER pathway. Although excessive HRD1 expression causes ER stress-mediated apoptosis, HRD1 also reduces misfolded proteins to preserve Treg cell function under physiologically normal intestinal conditions [45]. Similarly, HRD1 improves pancreatic b-cell function by efficiently degrading misfolded proinsulin, but it also impairs b-cell insulin secretion by ubiquitinating MAFA in diabetic patients [46, 47]. These findings indicate that the HRD1 substrates may differ under physiological and pathological conditions. Hence, related research is required to delve deeper into the role of HRD1.

Herein, we used RNA-seq analysis to identify crucial ubiquitination-associated molecules involved in intestinal I/R. Additionally, HRD1 could promote the ubiquitinated degradation of TMEM2 and thus facilitate ER stress-mediated apoptosis through a non-canonical pathway, suggesting that a potential treatment approach for intestinal I/R could involve focusing on the HRD1/TMEM2 axis.

## Materials and methods

### Animal experiments

Eight- to ten-week-old male C57BL/6 mice weighing 17–23 g were supplied with a standard laboratory chow nutrition and water. Mice were assigned to groups at random, with 10–12 mice in each group. Briefly, after anesthesia, the superior mesenteric artery was blocked for 45 min before reperfusion for 4 h [48]. The same steps were performed on sham mice in the absence of vascular obstruction. Following I/R, the mice were killed, and specimens were collected for examination. Male C57BL/6 mice from Dalian Medical University were injected with AAV-shRNA-HRD1, AAV-shRNA-TMEM2 and AAV-empty vectors three weeks before intestinal I/R treatment [49]. The adeno-associated virus (AAV) was stably expressed approximately three weeks after injection (Hanbio Biotechnology, Shanghai, China). The Institutional Ethics Committee of Dalian Medical University approved this study. This study followed the Guidelines for the Care and Use of Laboratory Animals.

### Patient tissue specimens

Terminal ileal specimens from six clinical patients were collected from the Second Hospital of Dalian Medical University. All patients provided a signed written notification of consent (Table S3). The procedure was approved by the Dalian Medical University Ethics Committee and performed in accordance with the Declaration of Helsinki.

### Cell hypoxia/reoxygenation and transient transfection

Caco-2 cells were cultured in a mixed MEM solution. We incubated the cells in a microaerobic system containing 94% N_2_, 1% O_2_ and 5% CO_2_ for 15 h to simulate hypoxia. For reoxygenation, the cells were grown on the condition of normal oxygen for 1, 2, 4 or 8 h. By cloning matching human full-length DNA sequence into Flag, MYC, or HA-pcDNA 3.1, Myc-HRD1, Myc-HRD1-N, Myc-HRD1-N-RING, Myc-HRD1-C, HA-Ub, HA-Ub-K48 (with the exception of lysine 48, all lysines in HA-Ub-K48 ubiquitin were changed to arginine), HA-Ub-K63 (with the exception of lysine 63, all lysines in HA-Ub-K63 ubiquitin were changed to arginine), Flag-TMEM2, Flag-TMEM2-K31R, Flag-TMEM2-K42R, Flag-TMEM2-K48R, Flag-TMEM2-K278R, and Flag-TMEM2-K1153R plasmids were acquired from GenePharma (Suzhou, China), and its mutant plasmid was created using infusion cloning kits. The plasmid sequences are shown in Table S2. Transfection of plasmids or siRNA (GenePharma, Suzhou, China) into Caco-2 cells was performed for 48 h before H/R with Lipofectamine 3000 (Invitrogen, USA). In addition, following pc-HRD1 transfection, the cells were treated for 3 h with 20 μM MG132 (Selleck, USA), and after HRD1 siRNA transfection, 100 μM cycloheximide (CHX, Sigma) was applied to Caco-2 cells for 0, 3, 6, 12 and 24 h. Furthermore, Caco-2 cells were transfected with si-TMEM2 and treated with 100 μg/ml LMWHA (Sigma Aldrich) for 24 h. After 8 h of PI3K/Akt inhibitor treatment with LY294002 (10 μmol/L) (Beyotime, Nantong, China), the cells were exposed to H/R. HEK293 cells were cultured in a DMEM mixed solution and transfected with the associated plasmids with Lipofectamine 3000 for 48 h before the Co-IP tests. The siRNA sequences were as follows: si-HRD1, 5′-GCAUGGCAGUCCUGUACAUTT-3′, and si-TMEM2, 5′-AUAAAUACCAUAUUCUCGCAUCCUG-3′.

### RNA-seq

For RNA-seq, total RNA was collected from I/R intestinal tissues as well as H/R Caco-2 cells using si-NC or si-TMEM2. cDNA libraries were constructed from total RNA to analyze gene expression differences. The concentration and purity of total RNA and the quality of the library were determined by Bosite Biotech (Shenyang, China). The PE150 sequencing strategy on the Illumina platform was used to sequence suitable libraries, and then the R package was used for subsequent data analysis.

### Co-IP/MS

At room temperature, the corresponding antibody was incubated with Protein A/G magnetic beads for 4 h. The supernatant was subsequently added to the antibody and bead mixture, and the mixture was incubated for another hour. Afterward, PBS was used to store the magnetic beads. To visualize the bead-bound proteins for MS, Coomassie Brilliant Blue staining was used to separate them using SDS-PAGE. Easy nLC 1200 chromatography and a Q Exactive HF-X mass spectrometer (Thermo Scientific) were used to obtain anti-HRD1 IP-specific peptides. For LC-MS/MS data analysis, MaxQuant 1.6.1.0 was used.

### Western blotting

Tissues and cells were lysed, and protein concentrations were determined. Antibodies: HRD1, c-caspase12, Flag, Myc, HA, PI3k, Akt and p-Akt (Proteintech); β-actin (Bimake); p-PI3k (Bioworld); TMEM2 (AVIVA); p-PERK, PERK, c-PARP, c-caspase3, ATF6, CHOP, p-IRE1, and IRE1 (ABclonal); and ubiquitin (Cell Signaling Technology). Gel-Pro Analyzer version 4.0 (Media Cybernetics, MD, USA) was used for protein quantification.

### RNA extraction, gDNA extraction, and qRT-PCR

TRIzol reagent (Invitrogen) was used to isolate total RNA from Caco-2 cells and intestinal tissues. PrimeScript RT Master Mix (TaKaRa, Japan) was used to generate cDNA. qRT‒PCR was carried out using the SYBR Premix Ex TaqTM II kit (TaKaRa). The PCR primers are shown in Table S1.

### TUNEL assay

Apoptosis caused by H/R or I/R was measured using the One-step TUNEL In Situ Apoptosis Kit (Elabscience, Wuhan, China). Paraffin sections of the intestine were deparaffinized with xylene, soaked in different concentrations of alcohol, fixed with apposed cells, and permeabilized with paraformaldehyde and Triton X-100. Then, TUNEL and DAPI were used to stain the adherent cells and paraffin sections.

### Caspase-3 activity determination

The caspase-3 activity assay was carried out using the GreenNuc™ Caspase-3 Assay Kit (Beyotime, Nanjing, China). Apoptotic cells were labeled with GreenNuc™ Caspase-3 substrate for 30 min at 37 °C. Afterward, DAPI was added sequentially to the adherent cells.

### CellTiter-Glo luminescence viability assay

We used tunicamycin or dithiothreitol (DTT) to induce ER stress. Cells were cultivated in 96-well plates with the density of 5 × 10^3^ cells/well and treated with tunicamycin (5 μg/ml, 24 h) or DTT (2 mM, 24 h) (MedChemExpress). After H/R, 200 µl CellTiter-Glo® reagent (CellTiter-Glo® Luminescent Cell Viability Assay) was added to the plates. After incubation for 20 min at room temperature, luminometer readings were taken.

### Histology and immunofluorescence staining

Sections of excised intestinal tissue were immobilized in paraformaldehyde for at least one night and then stained with hematoxylin-eosin (H&E). Histological damage in the intestine, liver, and lung was scored using the Chiu, Mikawa, and Eckhoff scoring systems, respectively. Immunohistochemistry (IHC) was performed by incubating intestinal paraffin sections with HRD1 antibodies (13473-1-AP, Proteintech) and then staining. Finally, light microscopy was used to visualize the sections.

### Statistical analysis

All data were presented as the means ± standard deviations (SD) using GraphPad Prism software. We employed an unpaired two-tailed Student’s *t* test for two-group comparisons and conducted statistical comparisons among multiple groups using one-way ANOVA, followed by Tukey’s test for datasets with homogeneous variances or Dunnett’s T3 test for datasets exhibiting heterogeneity of variance. *P* value < 0.05 was considered to indicate a significant difference.

### Supplementary information

Supplementary Material

Original western blots

Checklist

## Data Availability

The datasets used and/or analyzed during the current study are available from the corresponding author on reasonable request. The RNA-seq data have been deposited at GEO (https://www.ncbi.nlm.nih.gov/geo/): GSE232246, GSE233710, and GSE232090.
