# Supplementary material for: HRD1-induced TMEM2 ubiquitination promotes ER stress-mediated apoptosis through a non-canonical pathway in intestinal ischemia/reperfusion
Source: Cell Death Dis. 2024 Feb 20;15(2):154. doi: 10.1038/s41419-024-06504-0 (PMC10879504; doi:10.1038/s41419-024-06504-0)
Supplement: Supplementary file 1 — Supplementary Material [file 41419_2024_6504_MOESM1_ESM.pdf]

Supplementary Material 1

Supplementary Table 1. Primer sequences.

Gene	Forward primer (5′–3′)	Reverse primer (5′–3′)
β-actin (human)	CGTGGACATCCGCAAAGA	GAAGGTGGACAGCGAGGC
TMEM2 (human)	ATGCAAACCCCGAAAAACC	CACTGGCAAAGGTCAGTCCTATT
β-actin (mouse)	GTGCTATGTTGCTCTAGACTTCG	ATGCCACAGGATTCCATACC
HRD1 (mouse)	TCCAGGCCTTTGTCCTTGTC	CATCCCGAAAAACGGTGAAG
Rffl (mouse)	CCAGCGGGAGGAGCTCAT	GTCCTGTCCGCCTCAGAAATT
TMEM2 (mouse)	CAGAAGAAGCCGTCTGAGAGAAA	GGAGTCTGTTGCTGCCTGAAT

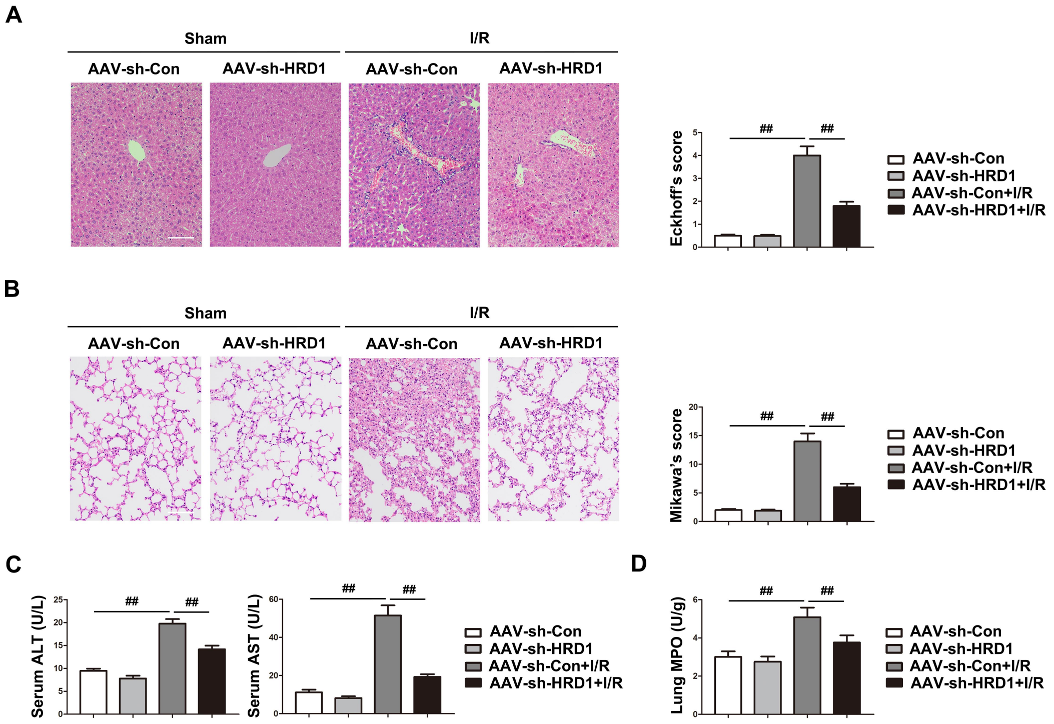

Supplementary Figure 1. HRD1 aggravates remote organ injury induced by intestinal I/R in mice. (A-D) AAV-sh-HRD1 or AAV empty vectors were injected into C57BL/6 mice for three weeks before intestinal I/R challenge (n=6). (A) Liver H&E staining (Scale bar=100 μm). (B) Lung H&E staining (Scale bar=100 μm). (C) Levels of ALT and AST (n=6). (D) Activity of lung MPO (n=6). $^{\#\#}P < 0.01$.

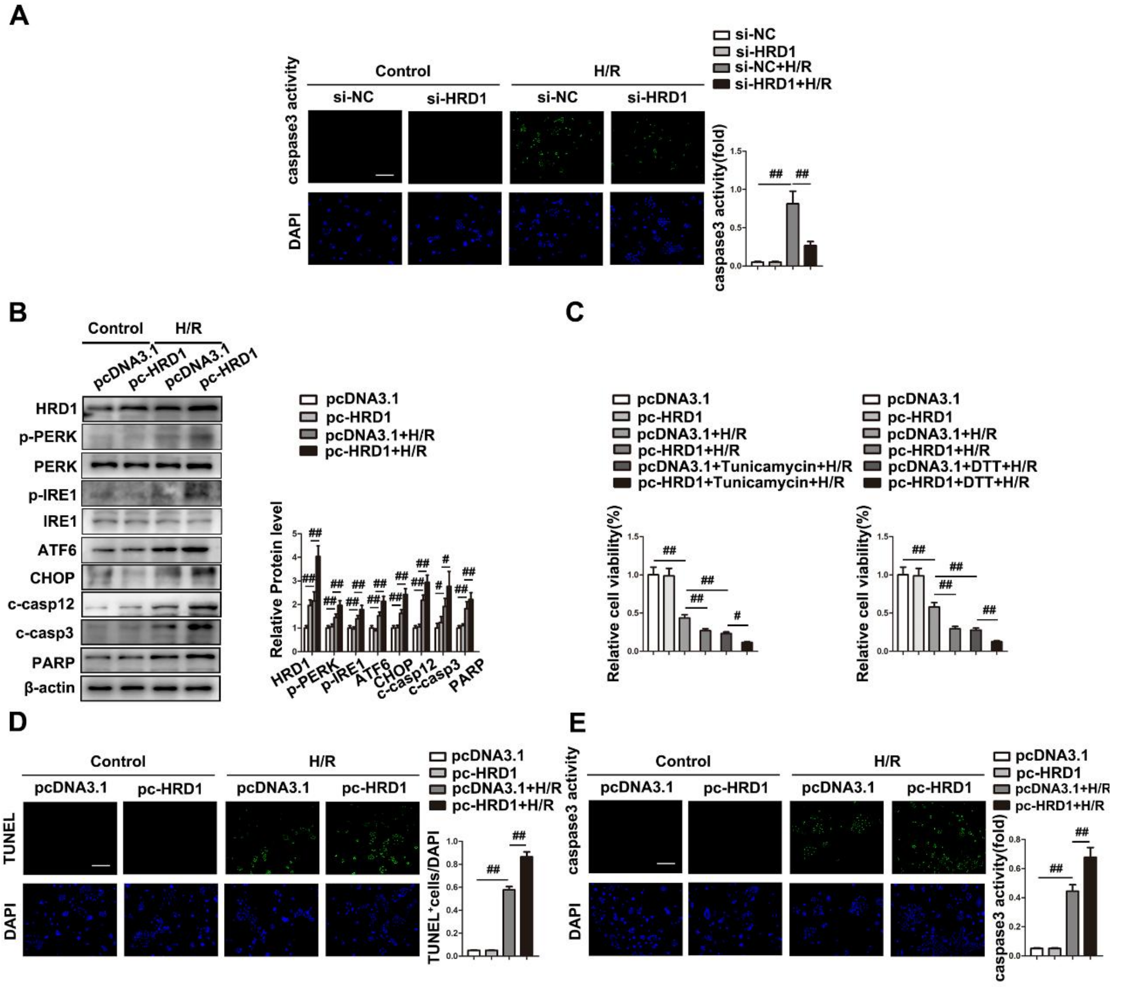

Supplementary Figure 2. HRD1 overexpression promotes ER stress-mediated apoptosis in intestinal I/R. (A) Caco-2 cells were transfected with si-HRD1 or si-NC before H/R challenge (n=3). Caspase-3 activity (Scale bar=100 μm). (B-E) Before the H/R challenge, Caco-2 cells were transfected with pc-HRD1 or pcDNA3.1 (n=3). (B) Protein expressions (n=3). (C) Relative cell viability was measured through CTG analysis (n = 6). (D) TUNEL staining (Scale bar=100 μm). (E) Caspase-3 activity (Scale bar=100 μm). $^{\#}P < 0.05$, $^{\#\#}P < 0.01$.

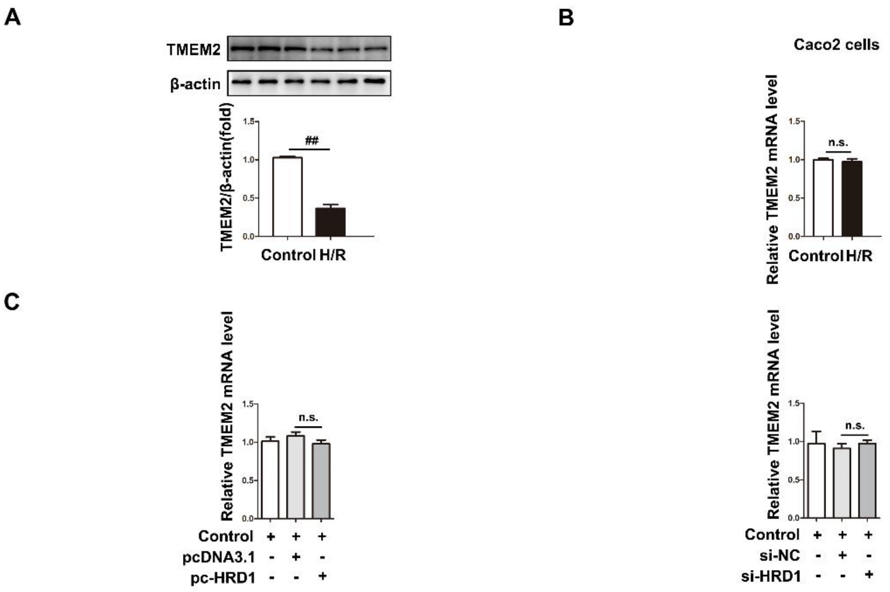

Supplementary Figure 3. TMEM2 expression in Caco-2 cells under H/R conditions. (A) TMEM2 protein expression (n=3). (B-C) TMEM2 mRNA levels (n=6). $^{\#\#}$P<0.01.

A

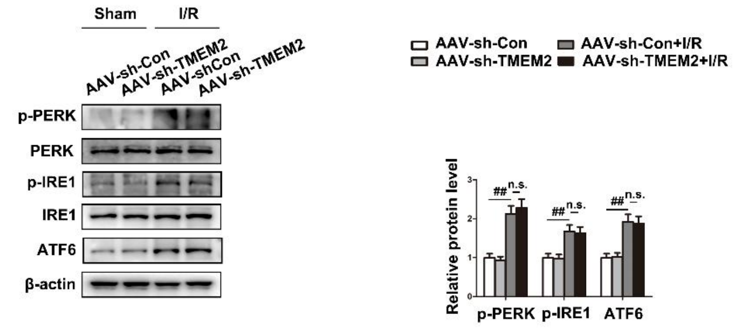

Supplementary Figure 4. TMEM2 knockdown has no impact on canonical ER stress pathway genes. (A) AAV-sh-TMEM2 or AAV empty vectors were injected into C57BL/6 mice for three weeks before intestinal I/R challenge (n=6). p-PERK, ATF6, and p-IRE1 protein expressions (n=3). $^{\#\#}P < 0.01$.

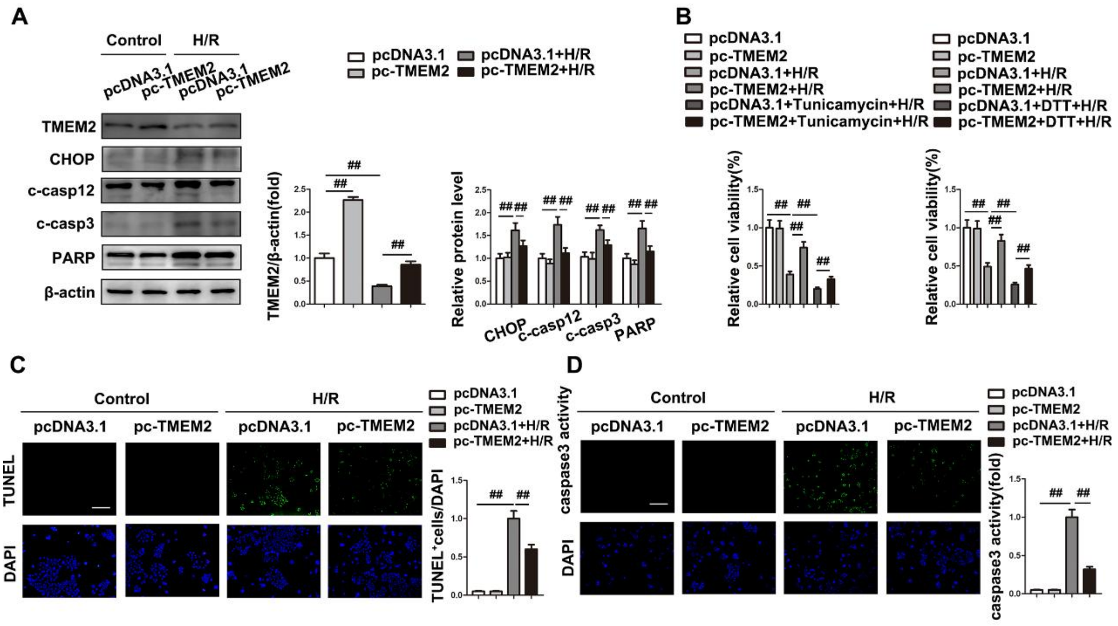

Supplementary Figure 5. TMEM2 overexpression attenuates ER stress-mediated apoptosis in intestinal I/R. (A-D) Before H/R challenge, Caco-2 cells were transfected with pc-TMEM2 or pcDNA3.1 (n=3). (A) Protein expressions (n=3). (B) Relative cell viability was measured through CTG analysis (n = 6). (C) TUNEL staining (Scale bar=100 μm). (D) Caspase-3 activity (Scale bar=100 μm). $^{\#\#}P < 0.01$.

Supplementary Material 2

Supplementary Table 2. Plasmid sequences.

Flag-TMEM2	ATGTATGCCACTGATTCCAGGGGACACTCCCCTGCTTTCCTCCAAC<br>CTCAGAATGGAAATAGTCGTCACCCATCTGGCTATGTTCCAGGGA<br>AGGTTGTCCCATTGCGTCCCCCTCCTCCTCCAAAGAGTCAAGCTTC<br>AGCCAAATTTACCTCCATCAGACGAGAAGACCGGGCAACCTTCGC<br>ATTCTCACCTGAAGAACAGCAAGCCCAGAGAGAAAGTCAAAAGC<br>AAAAGAGACACAAAAATACTTTCATTTGTTTTGCTATTACTAGTTT<br>CTCATTTTTTATTGCACTTGCAATCATTTTAGGAATATCCTCAAAAT<br>ATGCTCCAGATGAAAATTGCCCAGATCAAAATCCTCGTCTCAGGA<br>ATTGGGATCCAGGACAAGATTCTGCAAAGCAAGTTGTTATCAAGG<br>AGGGAGATATGCTCCGTCTGACCTCAGACGCCACCGTGCATTCTAT<br>AGTCATTCAGGATGGAGGACTGCTTGTATTTGGGGACAATAAAGA<br>TGGATCCAGAAATATTACTTTGAGGACTCATTACATCCTGATCCAG<br>GATGGTGGGGCGCTTCATATTGGAGCAGAAAAATGCCGCTATAAA<br>TCCAAAGCGACAATTACCTTGTATGGCAAGTCAGATGAAGGTGAA<br>AGTATGCCAACATTTGGCAAAAAGTTTATTGGTGTGGAAGCTGGC<br>GGGACACTGGAGTTACATGGGGCACGGAAGGCATCGTGGACGTTG<br>TTGGCAAGGACCCTGAATTCCTCAGGCTTGCCCTTTGGGTCCTATA<br>CCTTTGAAAAGGACTTTTCCCGGGGCCTCAATGTGAGGGTCATTGA<br>CCAAGACACGGCCAAAATTTTGGAAAGTGAGAGATTTGATACCCA<br>TGAATACCGCAATGAGAGCAGGCGGCTTCAGGAGTTTCTGAGATT<br>CCAGGATCCAGGTCGGATTGTTGCCATAGCTGTCGGGGATTCAGC<br>CGCTAAAAGTCTCTTACAAGGAACCATCCAGATGATCCAGGAACG<br>GTTGGGAAGTGAACTGATCCAAGGACTGGGCTACAGGCAAGCTTG<br>GGCTTTAGTTGGTGTCATTGATGGTGGAAGCACTTCTTGCAATGAA<br>TCCGTGAGAAACTATGAAAATCATAGCAGTGGCGGGAAGGCTCTT<br>GCCCAAAGAGAATTTTATACTGTGGATGGCCAGAAGTTCTCTGTG<br>ACAGCTTATAGTGAATGGATTGAAGGCGTTTCTCTTTCAGGATTCC<br>GGGTAGAGGTTGTAGATGGAGTGAAGCTAAATTTGCTAGATGATG<br>TTAGTAGTTGGAAACCTGGAGACCAGATTGTGGTCGCAAGCACAG<br>ACTATTCCATGTACCAAGCAGAGGAGTTCACTCTTCTTCCCTGTTC<br>TGAATGCAGCCATTTTCAGGTCAAAGTCAAAGAAACCCCTCAGTT<br>CCTGCACATGGGTGAGATCATAGACGGTGTAGACATGAGAGCTGA<br>GGTTGGAATTCTTACCCGGAATATTGTGATCCAAGGAGAAGTGGA<br>GGACTCATGCTACGCAGAAAATCAGTGCCAATTTTTTGATTATGAT<br>ACCTTTGGGGGACACATTATGATAATGAAAAATTTTACTTCAGTCC<br>ATCTTTCTTATGTGGAATTGAAACACATGGGTCAGCAGCAGATGG<br>GGCGATACCCTGTTCATTTTCACCTGTGTGGTGACGTGGATTATAA<br>AGGAGGATACAGACATGCAACATTTGTGGACGGCCTGTCTATTCA<br>TCACAGCTTCTCAAGGTGCATCACTGTGCATGGGACAAATGGCTT<br>GCTAATAAAAGACACCATTGGGTTTGACACACTAGGTCATTGTTTC<br>TTTTTGGAAGATGGTATTGAACAGAGGAATACTTTGTTCCACAATC<br>TGGGACTCCTCACCAAGCCGGGTACTCTCCTGCCCACCGATAGGA<br>ACAACTCCATGTGTACCACCATGCGAGATAAAGTGTTTGGAAATT

	ACATTCCTGTGCCTGCTACTGACTGTATGGCTGTTTCAACTTTCTG<br>GATTGCTCATCCCAACAATAATCTGATTAATAATGCAGCTGCAGG<br>CTCACAGGATGCTGGAATATGGTATTTATTCCACAAGGAACCAAC<br>TGGGGAATCCAGTGGATTGCAGCTCTTGGCAAAACCAGAACTCAC<br>TCCATTGGGTATATTTTATAACAACAGGGTCCATTCAAATTTTAAG<br>GCTGGCTTATTTATTGACAAAGGTGTCAAAACAACCAACTCTAGT<br>GCTGCTGACCCAAGGGAATACCTCTGTTTGGACAATAGTGCAAGA<br>TTTCGACCTCATCAGGATGCAAACCCCGAAAAACCACGTGTTGCT<br>GCTCTAATTGACAGGCTCATTGCTTTTAAAAATAATGATAATGGAG<br>CTTGGGTCAGAGGAGGAGATATTATCGTTCAAAATTCAGCATTTG<br>CAGATAATGGAATAGGACTGACCTTTGCCAGTGATGGAAGCTTCC<br>CAAGTGATGAAGGTTCCAGCCAAGAGGTATCTGAATCTCTCTTTGT<br>TGGGGAGAGCAGGAATTACGGCTTTCAGGGTGGTCAGAACAAGTA<br>TGTAGGCACTGGAGGAATAGACCAGAAGCCTCGAACATTACCCAG<br>GAACAGGACGTTCCCAATTAGAGGCTTTCAGATTTATGATGGGCC<br>CATTCATCTCACAAGGAGCACTTTCAAAAAATATGTGCCAACTCC<br>AGATAGGTACAGCAGTGCAATTGGCTTCCTCATGAAGAATTCCTG<br>GCAGATAACCCCCAGGAATAATATCTCCCTCGTGAAGTTTGGTCC<br>ACATGTCTCTCTGAATGTCTTTTTTGGAAAGCCTGGTCCCTGGTTT<br>GAAGATTGTGAGATGGATGGTGATAAGAACTCCATATTCCATGAC<br>ATTGATGGCTCTGTGACAGGATACAAGGATGCTTATGTGGGAAGA<br>ATGGACAACTACCTGATCCGCCATCCAAGCTGTGTAAATGTGTCTA<br>AGTGGAATGCAGTGATCTGCAGTGGGACCTATGCACAGGTCTATG<br>TACAGACATGGAGCACTCAGAATCTTTCTATGACCATTACACGAG<br>ATGAGTATCCGTCCAACCCTATGGTGCTCCGAGGTATTAATCAGA<br>AGGCTGCCTTTCCACAGTACCAGCCTGTCGTCATGCTGGAGAAGG<br>GTTATACCATCCACTGGAATGGGCCGGCACCACGGACTACATTTCT<br>ATACCTCGTCAACTTCAACAAGAATGACTGGATTCGAGTTGGCCTT<br>TGCTATCCATCAAACACAAGTTTTCAAGTTACCTTTGGCTATTTGC<br>AGCGGCAGAATGGCTCATTATCCAAAATCGAAGAATATGAGCCTG<br>TGCATTCACTGGAAGAACTGCAAAGAAAGCAATCCGAGAGGAAA<br>TTCTATTTTGACTCCAGCACGGGGTTACTGTTTTTGTATCTCAAAG<br>CCAAAAGCCACAGGCATGGCCACAGTTACTGTTCATCTCAGGGAT<br>GTGAAAGAGTCAAGATCCAAGCAGCCACAGACTCAAAGGACATC<br>AGTAACTGCATGGCCAAAGCATACCCACAGTACTACAGAAAGCCG<br>TCAGTGGTCAAGCGGATGCCGGCCATGCTCACTGGACTCTGTCAA<br>GGCTGTGGCACTCGGCAGGTGGTGTTTACTAGTGATCCTCATAAA<br>AGTTACCTCCCTGTGCAATTCCAGTCACCTGATAAAGCAGAAACC<br>CAGCGTGGAGACCCGTCTGTTATTTCTGTCAATGGCACTGACTTTA<br>CCTTCCGAAGTGCAGGCGTCCTCCTCCTTGTTGTGGATCCGTGCAG<br>CGTTCCATTCCGCTTGACGGAAAAAACGGTTTTTCCTCTTGCTGAT<br>GTCAGTCGCATTGAAGAGTATTTAAAAACAGGCATCCCTCCAAGG<br>TCCATTGTTCTGTTGAGCACAAGAGGAGAAATAAAGCAGTTAAAC<br>ATTTCACACTTACTAGTACCTCTGGGATTAGCCAAACCAGCTCATC

	TTTATGACAAAGGGAGTACCATATTTTTGGGATTCAGTGGAAACTT<br>TAAACCATCATGGACTAAGCTATTTACCAGTCCTGCTGGACAGGG<br>CCTTGGGGTGCTTGAACAATTCATACCTTTGCAGCTGGACGAATAT<br>GGTTGTCCCAGAGCCACCACTGTCCGCAGAAGAGACCTGGAACTG<br>CTAAAGCAAGCTTCAAAAGCACAT
Myc-HRD1	TGTTCCGCACGGCAGTGATGATGGCGGCCAGCCTGGCGCTGACCG<br>GGGCTGTGGTGGCTCACGCCTACTACCTCAAACACCAGTTCTACCC<br>CACTGTGGTGTACCTGACCAAGTCCAGCCCCAGCATGGCAGTCCT<br>GTACATCCAGGCCTTTGTCCTTGTCTTCCTTCTGGGCAAGGTGATG<br>GGCAAGGTGTTCTTTGGGCAACTGAGGGCAGCAGAGATGGAGCAC<br>CTTCTGGAACGTTCCTGGTACGCCGTCACAGAGACTTGTCTGGCCT<br>TCACCGTTTTTCGGGATGACTTCAGCCCCCGCTTTGTTGCACTCTTC<br>ACTCTTCTTCTCTTCCTCAAATGTTTCCACTGGCTGGCTGAGGACC<br>GTGTGGACTTTATGGAACGCAGCCCCAACATCTCCTGGCTCTTTCA<br>CTGCCGCATTGTCTCTCTTATGTTCCTCCTGGGCATCCTGGACTTCC<br>TCTTCGTCAGCCACGCCTATCACAGCATCCTGACCCGTGGGGCCTC<br>TGTGCAGCTGGTGTTTGGCTTTGAGTATGCCATCCTGATGACGATG<br>GTGCTCACCATCTTCATCAAGTATGTGCTGCACTCCGTGGACCTCC<br>AGAGTGAGAACCCCTGGGACAACAAGGCTGTGTACATGCTCTACA<br>CAGAGCTGTTTACAGGCTTCATCAAGGTTCTGCTGTACATGGCCTT<br>CATGACCATCATGATCAAGGTGCACACCTTCCCACTCTTTGCCATC<br>CGGCCCATGTACCTGGCCATGAGACAGTTCAAGAAAGCTGTGACA<br>GATGCCATCATGTCTCGCCGAGCCATCCGCAACATGAACACCCTG<br>TATCCAGATGCCACCCCAGAGGAGCTCCAGGCAATGGACAATGTC<br>TGCATCATCTGCCGAGAAGAGATGGTGACTGGTGCCAAGAGACTG<br>CCCTGCAACCACATTTTCCATACCAGCTGCCTGCGCTCCTGGTTCC<br>AGCGGCAGCAGACCTGCCCCACCTGCCGTATGGATGTCCTTCGTG<br>CATCGCTGCCAGCGCAGTCACCACCACCCCCGGAGCCTGCGGATC<br>AGGGGCCACCCCCTGCCCCCCACCCCCCACCACTCTTGCCTCAGCC<br>CCCCAACTTCCCCCAGGGCCTCCTGCCTCCTTTTCCTCCAGGCATG<br>TTCCCACTGTGGCCCCCCATGGGCCCCTTTCCACCTGTCCCGCCTC<br>CCCCCAGCTCAGGAGAGGCTGTGGCTCCTCCATCCACCAGTGCAG<br>CCCTTTCTCGGCCCAGTGGAGCAGCTACAACCACAGCTGCTGGCA<br>CCAGTGCTACTGCTGCTTCTGCCACAGCATCTGGCCCAGGCTCTGG<br>CTCTGCCCCAGAGGCTGGCCCTGCCCCTGGTTTCCCCTTCCCTCCT<br>CCCTGGATGGGTATGCCCCTGCCTCCACCCTTTGCCTTCCCCCCAA<br>TGCCTGTGCCCCCTGCGGGCTTTGCTGGGCTGACCCCAGAGGAGCT<br>ACGAGCTCTGGAGGGCCATGAGCGGCAGCACCTGGAGGCCCGGCT<br>GCAGAGCCTGCGTAACATCCACACACTGCTGGACGCCGCCATGCT<br>GCAGATCAACCAGTACCTCACCGTGCTGGCCTCCTTGGGGCCCCCC<br>CGGCCTGCCACTTCAGTCAACTCCACTGAGGAGACTGCCACTACA<br>GTTGTTGCTGCTGCCTCCTCCACCAGCATCCCTAGCTCAGAGGCCA<br>CGACCCCAACCCCAGGAGCCTCCCCACCAGCCCCTGAAATGGAAA<br>GGCCTCCAGCTCCTGAGTCAGTGGGCACAGAGGAGATGCCTGAGG

	ATGGAGAGCCCGATGCAGCAGAGCTCCGCCGGCGCCGCCTGCAGA<br>AGCTGGAGTCTCCTGTTGCCCAC<br>CACTGGCAAAGGTCAGTCCTATT
Flag-TMEM2-K31R	ATGTATGCCACTGATTCCAGGGGACACTCCCCTGCTTTCCTCCAAC<br>CTCAGAATGGAAATAGTCGTCACCCATCTGGCTATGTTCCAGGGA<br>GGGTTGTCCCATTGCGTCCCCCTCCTCCTCCAAAGAGTCAAGCTTC<br>AGCCAAATTTACCTCCATCAGACGAGAAGACCGGGCAACCTTCGC<br>ATTCTCACCTGAAGAACAGCAAGCCCAGAGAGAAAGTCAAAAGC<br>AAAAGAGACACAAAAATACTTTCATTTGTTTTGCTATTACTAGTTT<br>CTCATTTTTTATTGCACTTGCAATCATTTTAGGAATATCCTCAAAAT<br>ATGCTCCAGATGAAAATTGCCCAGATCAAAATCCTCGTCTCAGGA<br>ATTGGGATCCAGGACAAGATTCTGCAAAGCAAGTTGTTATCAAGG<br>AGGGAGATATGCTCCGTCTGACCTCAGACGCCACCGTGCATTCTAT<br>AGTCATTCAGGATGGAGGACTGCTTGTATTTGGGGACAATAAAGA<br>TGGATCCAGAAATATTACTTTGAGGACTCATTACATCCTGATCCAG<br>GATGGTGGGGCGCTTCATATTGGAGCAGAAAAATGCCGCTATAAA<br>TCCAAAGCGACAATTACCTTGTATGGCAAGTCAGATGAAGGTGAA<br>AGTATGCCAACATTTGGCAAAAAGTTTATTGGTGTGGAAGCTGGC<br>GGGACACTGGAGTTACATGGGGCACGGAAGGCATCGTGGACGTTG<br>TTGGCAAGGACCCTGAATTCCTCAGGCTTGCCCTTTGGGTCCTATA<br>CCTTTGAAAAGGACTTTTCCCGGGGCCTCAATGTGAGGGTCATTGA<br>CCAAGACACGGCCAAAATTTTGGAAAGTGAGAGATTTGATACCCA<br>TGAATACCGCAATGAGAGCAGGCGGCTTCAGGAGTTTCTGAGATT<br>CCAGGATCCAGGTCGGATTGTTGCCATAGCTGTCGGGGATTCAGC<br>CGCTAAAAGTCTCTTACAAGGAACCATCCAGATGATCCAGGAACG<br>GTTGGGAAGTGAACTGATCCAAGGACTGGGCTACAGGCAAGCTTG<br>GGCTTTAGTTGGTGTCATTGATGGTGGAAGCACTTCTTGCAATGAA<br>TCCGTGAGAAACTATGAAAATCATAGCAGTGGCGGGAAGGCTCTT<br>GCCCAAAGAGAATTTTATACTGTGGATGGCCAGAAGTTCTCTGTG<br>ACAGCTTATAGTGAATGGATTGAAGGCGTTTCTCTTTCAGGATTCC<br>GGGTAGAGGTTGTAGATGGAGTGAAGCTAAATTTGCTAGATGATG<br>TTAGTAGTTGGAAACCTGGAGACCAGATTGTGGTCGCAAGCACAG<br>ACTATTCCATGTACCAAGCAGAGGAGTTCACTCTTCTTCCCTGTTC<br>TGAATGCAGCCATTTTCAGGTCAAAGTCAAAGAAACCCCTCAGTT<br>CCTGCACATGGGTGAGATCATAGACGGTGTAGACATGAGAGCTGA<br>GGTTGGAATTCTTACCCGGAATATTGTGATCCAAGGAGAAGTGGA<br>GGACTCATGCTACGCAGAAAATCAGTGCCAATTTTTTGATTATGAT<br>ACCTTTGGGGGACACATTATGATAATGAAAAATTTTACTTCAGTCC<br>ATCTTTCTTATGTGGAATTGAAACACATGGGTCAGCAGCAGATGG<br>GGCGATACCCTGTTCATTTTCACCTGTGTGGTGACGTGGATTATAA<br>AGGAGGATACAGACATGCAACATTTGTGGACGGCCTGTCTATTCA<br>TCACAGCTTCTCAAGGTGCATCACTGTGCATGGGACAAATGGCTT<br>GCTAATAAAAGACACCATTGGGTTTGACACACTAGGTCATTGTTTC<br>TTTTTGGAAGATGGTATTGAACAGAGGAATACTTTGTTCCACAATC

	TGGGACTCCTCACCAAGCCGGGTACTCTCCTGCCCACCGATAGGA<br>ACAACTCCATGTGTACCACCATGCGAGATAAAGTGTTTGGAAATT<br>ACATTCCTGTGCCTGCTACTGACTGTATGGCTGTTTCAACTTTCTG<br>GATTGCTCATCCCAACAATAATCTGATTAATAATGCAGCTGCAGG<br>CTCACAGGATGCTGGAATATGGTATTTATTCCACAAGGAACCAAC<br>TGGGGAATCCAGTGGATTGCAGCTCTTGGCAAAACCAGAACTCAC<br>TCCATTGGGTATATTTTATAACAACAGGGTCCATTCAAATTTTAAG<br>GCTGGCTTATTTATTGACAAAGGTGTCAAAACAACCAACTCTAGT<br>GCTGCTGACCCAAGGGAATACCTCTGTTTGGACAATAGTGCAAGA<br>TTTCGACCTCATCAGGATGCAAACCCCGAAAAACCACGTGTTGCT<br>GCTCTAATTGACAGGCTCATTGCTTTTAAAAATAATGATAATGGAG<br>CTTGGGTCAGAGGAGGAGATATTATCGTTCAAAATTCAGCATTTG<br>CAGATAATGGAATAGGACTGACCTTTGCCAGTGATGGAAGCTTCC<br>CAAGTGATGAAGGTTCCAGCCAAGAGGTATCTGAATCTCTCTTTGT<br>TGGGGAGAGCAGGAATTACGGCTTTCAGGGTGGTCAGAACAAGTA<br>TGTAGGCACTGGAGGAATAGACCAGAAGCCTCGAACATTACCCAG<br>GAACAGGACGTTCCCAATTAGAGGCTTTCAGATTTATGATGGGCC<br>CATTCATCTCACAAGGAGCACTTTCAAAAAATATGTGCCAACTCC<br>AGATAGGTACAGCAGTGCAATTGGCTTCCTCATGAAGAATTCCTG<br>GCAGATAACCCCCAGGAATAATATCTCCCTCGTGAAGTTTGGTCC<br>ACATGTCTCTCTGAATGTCTTTTTTGGAAAGCCTGGTCCCTGGTTT<br>GAAGATTGTGAGATGGATGGTGATAAGAACTCCATATTCCATGAC<br>ATTGATGGCTCTGTGACAGGATACAAGGATGCTTATGTGGGAAGA<br>ATGGACAACTACCTGATCCGCCATCCAAGCTGTGTAAATGTGTCTA<br>AGTGGAATGCAGTGATCTGCAGTGGGACCTATGCACAGGTCTATG<br>TACAGACATGGAGCACTCAGAATCTTTCTATGACCATTACACGAG<br>ATGAGTATCCGTCCAACCCTATGGTGCTCCGAGGTATTAATCAGA<br>AGGCTGCCTTTCCACAGTACCAGCCTGTCGTCATGCTGGAGAAGG<br>GTTATACCATCCACTGGAATGGGCCGGCACCACGGACTACATTTCT<br>ATACCTCGTCAACTTCAACAAGAATGACTGGATTCGAGTTGGCCTT<br>TGCTATCCATCAAACACAAGTTTTCAAGTTACCTTTGGCTATTTGC<br>AGCGGCAGAATGGCTCATTATCCAAAATCGAAGAATATGAGCCTG<br>TGCATTCACTGGAAGAACTGCAAAGAAAGCAATCCGAGAGGAAA<br>TTCTATTTTGACTCCAGCACGGGGTTACTGTTTTTGTATCTCAAAG<br>CCAAAAGCCACAGGCATGGCCACAGTTACTGTTCATCTCAGGGAT<br>GTGAAAGAGTCAAGATCCAAGCAGCCACAGACTCAAAGGACATC<br>AGTAACTGCATGGCCAAAGCATACCCACAGTACTACAGAAAGCCG<br>TCAGTGGTCAAGCGGATGCCGGCCATGCTCACTGGACTCTGTCAA<br>GGCTGTGGCACTCGGCAGGTGGTGTTTACTAGTGATCCTCATAAA<br>AGTTACCTCCCTGTGCAATTCCAGTCACCTGATAAAGCAGAAACC<br>CAGCGTGGAGACCCGTCTGTTATTTCTGTCAATGGCACTGACTTTA<br>CCTTCCGAAGTGCAGGCGTCCTCCTCCTTGTTGTGGATCCGTGCAG<br>CGTTCCATTCCGCTTGACGGAAAAAACGGTTTTTCCTCTTGCTGAT<br>GTCAGTCGCATTGAAGAGTATTTAAAAACAGGCATCCCTCCAAGG

	TCCATTGTTCTGTTGAGCACAAGAGGAGAAATAAAGCAGTTAAAC<br>ATTTCACACTTACTAGTACCTCTGGGATTAGCCAAACCAGCTCATC<br>TTTATGACAAAGGGAGTACCATATTTTTGGGATTCAGTGGAAACTT<br>TAAACCATCATGGACTAAGCTATTTACCAGTCCTGCTGGACAGGG<br>CCTTGGGGTGCTTGAACAATTCATACCTTTGCAGCTGGACGAATAT<br>GGTTGTCCCAGAGCCACCACTGTCCGCAGAAGAGACCTGGAACTG<br>CTAAAGCAAGCTTCAAAAGCACAT
Flag-TMEM2-K42R	ATGTATGCCACTGATTCCAGGGGACACTCCCCTGCTTTCCTCCAAC<br>CTCAGAATGGAAATAGTCGTCACCCATCTGGCTATGTTCCAGGGA<br>AGGTTGTCCCATTGCGTCCCCCTCCTCCTCCAAGGAGTCAAGCTTC<br>AGCCAAATTTACCTCCATCAGACGAGAAGACCGGGCAACCTTCGC<br>ATTCTCACCTGAAGAACAGCAAGCCCAGAGAGAAAGTCAAAAGC<br>AAAAGAGACACAAAAATACTTTCATTTGTTTTGCTATTACTAGTTT<br>CTCATTTTTTATTGCACTTGCAATCATTTTAGGAATATCCTCAAAAT<br>ATGCTCCAGATGAAAATTGCCCAGATCAAAATCCTCGTCTCAGGA<br>ATTGGGATCCAGGACAAGATTCTGCAAAGCAAGTTGTTATCAAGG<br>AGGGAGATATGCTCCGTCTGACCTCAGACGCCACCGTGCATTCTAT<br>AGTCATTCAGGATGGAGGACTGCTTGTATTTGGGGACAATAAAGA<br>TGGATCCAGAAATATTACTTTGAGGACTCATTACATCCTGATCCAG<br>GATGGTGGGGCGCTTCATATTGGAGCAGAAAAATGCCGCTATAAA<br>TCCAAAGCGACAATTACCTTGTATGGCAAGTCAGATGAAGGTGAA<br>AGTATGCCAACATTTGGCAAAAAGTTTATTGGTGTGGAAGCTGGC<br>GGGACACTGGAGTTACATGGGGCACGGAAGGCATCGTGGACGTTG<br>TTGGCAAGGACCCTGAATTCCTCAGGCTTGCCCTTTGGGTCCTATA<br>CCTTTGAAAAGGACTTTTCCCGGGGCCTCAATGTGAGGGTCATTGA<br>CCAAGACACGGCCAAAATTTTGGAAAGTGAGAGATTTGATACCCA<br>TGAATACCGCAATGAGAGCAGGCGGCTTCAGGAGTTTCTGAGATT<br>CCAGGATCCAGGTCGGATTGTTGCCATAGCTGTCGGGGATTCAGC<br>CGCTAAAAGTCTCTTACAAGGAACCATCCAGATGATCCAGGAACG<br>GTTGGGAAGTGAACTGATCCAAGGACTGGGCTACAGGCAAGCTTG<br>GGCTTTAGTTGGTGTCATTGATGGTGGAAGCACTTCTTGCAATGAA<br>TCCGTGAGAAACTATGAAAATCATAGCAGTGGCGGGAAGGCTCTT<br>GCCCAAAGAGAATTTTATACTGTGGATGGCCAGAAGTTCTCTGTG<br>ACAGCTTATAGTGAATGGATTGAAGGCGTTTCTCTTTCAGGATTCC<br>GGGTAGAGGTTGTAGATGGAGTGAAGCTAAATTTGCTAGATGATG<br>TTAGTAGTTGGAAACCTGGAGACCAGATTGTGGTCGCAAGCACAG<br>ACTATTCCATGTACCAAGCAGAGGAGTTCACTCTTCTTCCCTGTTC<br>TGAATGCAGCCATTTTCAGGTCAAAGTCAAAGAAACCCCTCAGTT<br>CCTGCACATGGGTGAGATCATAGACGGTGTAGACATGAGAGCTGA<br>GGTTGGAATTCTTACCCGGAATATTGTGATCCAAGGAGAAGTGGA<br>GGACTCATGCTACGCAGAAAATCAGTGCCAATTTTTTGATTATGAT<br>ACCTTTGGGGGACACATTATGATAATGAAAAATTTTACTTCAGTCC<br>ATCTTTCTTATGTGGAATTGAAACACATGGGTCAGCAGCAGATGG<br>GGCGATACCCTGTTCATTTTCACCTGTGTGGTGACGTGGATTATAA

	AGGAGGATACAGACATGCAACATTTGTGGACGGCCTGTCTATTCA<br>TCACAGCTTCTCAAGGTGCATCACTGTGCATGGGACAAATGGCTT<br>GCTAATAAAAGACACCATTGGGTTTGACACACTAGGTCATTGTTTC<br>TTTTTGGAAGATGGTATTGAACAGAGGAATACTTTGTTCCACAATC<br>TGGGACTCCTCACCAAGCCGGGTACTCTCCTGCCCACCGATAGGA<br>ACAACTCCATGTGTACCACCATGCGAGATAAAGTGTTTGGAAATT<br>ACATTCCTGTGCCTGCTACTGACTGTATGGCTGTTTCAACTTTCTG<br>GATTGCTCATCCCAACAATAATCTGATTAATAATGCAGCTGCAGG<br>CTCACAGGATGCTGGAATATGGTATTTATTCCACAAGGAACCAAC<br>TGGGGAATCCAGTGGATTGCAGCTCTTGGCAAAACCAGAACTCAC<br>TCCATTGGGTATATTTTATAACAACAGGGTCCATTCAAATTTTAAG<br>GCTGGCTTATTTATTGACAAAGGTGTCAAAACAACCAACTCTAGT<br>GCTGCTGACCCAAGGGAATACCTCTGTTTGGACAATAGTGCAAGA<br>TTTCGACCTCATCAGGATGCAAACCCCGAAAAACCACGTGTTGCT<br>GCTCTAATTGACAGGCTCATTGCTTTTAAAAATAATGATAATGGAG<br>CTTGGGTCAGAGGAGGAGATATTATCGTTCAAAATTCAGCATTTG<br>CAGATAATGGAATAGGACTGACCTTTGCCAGTGATGGAAGCTTCC<br>CAAGTGATGAAGGTTCCAGCCAAGAGGTATCTGAATCTCTCTTTGT<br>TGGGGAGAGCAGGAATTACGGCTTTCAGGGTGGTCAGAACAAGTA<br>TGTAGGCACTGGAGGAATAGACCAGAAGCCTCGAACATTACCCAG<br>GAACAGGACGTTCCCAATTAGAGGCTTTCAGATTTATGATGGGCC<br>CATTCATCTCACAAGGAGCACTTTCAAAAAATATGTGCCAACTCC<br>AGATAGGTACAGCAGTGCAATTGGCTTCCTCATGAAGAATTCCTG<br>GCAGATAACCCCCAGGAATAATATCTCCCTCGTGAAGTTTGGTCC<br>ACATGTCTCTCTGAATGTCTTTTTTGGAAAGCCTGGTCCCTGGTTT<br>GAAGATTGTGAGATGGATGGTGATAAGAACTCCATATTCCATGAC<br>ATTGATGGCTCTGTGACAGGATACAAGGATGCTTATGTGGGAAGA<br>ATGGACAACTACCTGATCCGCCATCCAAGCTGTGTAAATGTGTCTA<br>AGTGGAATGCAGTGATCTGCAGTGGGACCTATGCACAGGTCTATG<br>TACAGACATGGAGCACTCAGAATCTTTCTATGACCATTACACGAG<br>ATGAGTATCCGTCCAACCCTATGGTGCTCCGAGGTATTAATCAGA<br>AGGCTGCCTTTCCACAGTACCAGCCTGTCGTCATGCTGGAGAAGG<br>GTTATACCATCCACTGGAATGGGCCGGCACCACGGACTACATTTCT<br>ATACCTCGTCAACTTCAACAAGAATGACTGGATTCGAGTTGGCCTT<br>TGCTATCCATCAAACACAAGTTTTCAAGTTACCTTTGGCTATTTGC<br>AGCGGCAGAATGGCTCATTATCCAAAATCGAAGAATATGAGCCTG<br>TGCATTCACTGGAAGAACTGCAAAGAAAGCAATCCGAGAGGAAA<br>TTCTATTTTGACTCCAGCACGGGGTTACTGTTTTTGTATCTCAAAG<br>CCAAAAGCCACAGGCATGGCCACAGTTACTGTTCATCTCAGGGAT<br>GTGAAAGAGTCAAGATCCAAGCAGCCACAGACTCAAAGGACATC<br>AGTAACTGCATGGCCAAAGCATACCCACAGTACTACAGAAAGCCG<br>TCAGTGGTCAAGCGGATGCCGGCCATGCTCACTGGACTCTGTCAA<br>GGCTGTGGCACTCGGCAGGTGGTGTTTACTAGTGATCCTCATAAA<br>AGTTACCTCCCTGTGCAATTCCAGTCACCTGATAAAGCAGAAACC

	CAGCGTGGAGACCCGTCTGTTATTTCTGTCAATGGCACTGACTTTA<br>CCTTCCGAAGTGCAGGCGTCCTCCTCCTTGTTGTGGATCCGTGCAG<br>CGTTCCATTCCGCTTGACGGAAAAAACGGTTTTTCCTCTTGCTGAT<br>GTCAGTCGCATTGAAGAGTATTTAAAAACAGGCATCCCTCCAAGG<br>TCCATTGTTCTGTTGAGCACAAGAGGAGAAATAAAGCAGTTAAAC<br>ATTTCACACTTACTAGTACCTCTGGGATTAGCCAAACCAGCTCATC<br>TTTATGACAAAGGGAGTACCATATTTTTGGGATTCAGTGGAAACTT<br>TAAACCATCATGGACTAAGCTATTTACCAGTCCTGCTGGACAGGG<br>CCTTGGGGTGCTTGAACAATTCATACCTTTGCAGCTGGACGAATAT<br>GGTTGTCCCAGAGCCACCACTGTCCGCAGAAGAGACCTGGAACTG<br>CTAAAGCAAGCTTCAAAAGCACAT
Flag-TMEM2-K4<br>8R	ATGTATGCCACTGATTCCAGGGGACACTCCCCTGCTTTCCTCCAAC<br>CTCAGAATGGAAATAGTCGTCACCCATCTGGCTATGTTCCAGGGA<br>AGGTTGTCCCATTGCGTCCCCCTCCTCCTCCAAAGAGTCAAGCTTC<br>AGCCAGATTTACCTCCATCAGACGAGAAGACCGGGCAACCTTCGC<br>ATTCTCACCTGAAGAACAGCAAGCCCAGAGAGAAAGTCAAAAGC<br>AAAAGAGACACAAAAATACTTTCATTTGTTTTGCTATTACTAGTTT<br>CTCATTTTTTATTGCACTTGCAATCATTTTAGGAATATCCTCAAAAT<br>ATGCTCCAGATGAAAATTGCCCAGATCAAAATCCTCGTCTCAGGA<br>ATTGGGATCCAGGACAAGATTCTGCAAAGCAAGTTGTTATCAAGG<br>AGGGAGATATGCTCCGTCTGACCTCAGACGCCACCGTGCATTCTAT<br>AGTCATTCAGGATGGAGGACTGCTTGTATTTGGGGACAATAAAGA<br>TGGATCCAGAAATATTACTTTGAGGACTCATTACATCCTGATCCAG<br>GATGGTGGGGCGCTTCATATTGGAGCAGAAAAATGCCGCTATAAA<br>TCCAAAGCGACAATTACCTTGTATGGCAAGTCAGATGAAGGTGAA<br>AGTATGCCAACATTTGGCAAAAAGTTTATTGGTGTGGAAGCTGGC<br>GGGACACTGGAGTTACATGGGGCACGGAAGGCATCGTGGACGTTG<br>TTGGCAAGGACCCTGAATTCCTCAGGCTTGCCCTTTGGGTCCTATA<br>CCTTTGAAAAGGACTTTTCCCGGGGCCTCAATGTGAGGGTCATTGA<br>CCAAGACACGGCCAAAATTTTGGAAAGTGAGAGATTTGATACCCA<br>TGAATACCGCAATGAGAGCAGGCGGCTTCAGGAGTTTCTGAGATT<br>CCAGGATCCAGGTCGGATTGTTGCCATAGCTGTCGGGGATTCAGC<br>CGCTAAAAGTCTCTTACAAGGAACCATCCAGATGATCCAGGAACG<br>GTTGGGAAGTGAACTGATCCAAGGACTGGGCTACAGGCAAGCTTG<br>GGCTTTAGTTGGTGTCATTGATGGTGGAAGCACTTCTTGCAATGAA<br>TCCGTGAGAAACTATGAAAATCATAGCAGTGGCGGGAAGGCTCTT<br>GCCCAAAGAGAATTTTATACTGTGGATGGCCAGAAGTTCTCTGTG<br>ACAGCTTATAGTGAATGGATTGAAGGCGTTTCTCTTTCAGGATTCC<br>GGGTAGAGGTTGTAGATGGAGTGAAGCTAAATTTGCTAGATGATG<br>TTAGTAGTTGGAAACCTGGAGACCAGATTGTGGTCGCAAGCACAG<br>ACTATTCCATGTACCAAGCAGAGGAGTTCACTCTTCTTCCCTGTTC<br>TGAATGCAGCCATTTTCAGGTCAAAGTCAAAGAAACCCCTCAGTT<br>CCTGCACATGGGTGAGATCATAGACGGTGTAGACATGAGAGCTGA<br>GGTTGGAATTCTTACCCGGAATATTGTGATCCAAGGAGAAGTGGA

	GGACTCATGCTACGCAGAAAATCAGTGCCAATTTTTTGATTATGAT<br>ACCTTTGGGGGACACATTATGATAATGAAAAATTTACTTCAGTCC<br>ATCTTTCTTATGTGGAATTGAAACACATGGGTCAGCAGCAGATGG<br>GGCGATACCCTGTTCATTTTCACCTGTGTGGTGACGTGGATTATAA<br>AGGAGGATACAGACATGCAACATTTGTGGACGGCCTGTCTATTCA<br>TCACAGCTTCTCAAGGTGCATCACTGTGCATGGGACAAATGGCTT<br>GCTAATAAAAGACACCATTGGGTTTGACACACTAGGTCATTGTTTC<br>TTTTTGGAAGATGGTATTGAACAGAGGAATACTTTGTTCCACAATC<br>TGGGACTCCTCACCAAGCCGGGTACTCTCCTGCCCACCGATAGGA<br>ACAACTCCATGTGTACCACCATGCGAGATAAAGTGTTTGGAAATT<br>ACATTCCTGTGCCTGCTACTGACTGTATGGCTGTTTCAACTTTCTG<br>GATTGCTCATCCCAACAATAATCTGATTAATAATGCAGCTGCAGG<br>CTCACAGGATGCTGGAATATGGTATTTATTCCACAAGGAACCAAC<br>TGGGGAATCCAGTGGATTGCAGCTCTTGGCAAAACCAGAACTCAC<br>TCCATTGGGTATATTTTATAACAACAGGGTCCATTCAAATTTTAAG<br>GCTGGCTTATTTATTGACAAAGGTGTCAAAACAACCAACTCTAGT<br>GCTGCTGACCCAAGGGAATACCTCTGTTTGGACAATAGTGCAAGA<br>TTTCGACCTCATCAGGATGCAAACCCCGAAAAACCACGTGTTGCT<br>GCTCTAATTGACAGGCTCATTGCTTTTAAAAATAATGATAATGGAG<br>CTTGGGTCAGAGGAGGAGATATTATCGTTCAAAATTCAGCATTTG<br>CAGATAATGGAATAGGACTGACCTTTGCCAGTGATGGAAGCTTCC<br>CAAGTGATGAAGGTTCCAGCCAAGAGGTATCTGAATCTCTCTTTGT<br>TGGGGAGAGCAGGAATTACGGCTTTCAGGGTGGTCAGAACAAGTA<br>TGTAGGCACTGGAGGAATAGACCAGAAGCCTCGAACATTACCCAG<br>GAACAGGACGTTCCCAATTAGAGGCTTTCAGATTTATGATGGGCC<br>CATTCATCTCACAAGGAGCACTTTCAAAAAATATGTGCCAACTCC<br>AGATAGGTACAGCAGTGCAATTGGCTTCCTCATGAAGAATTCCTG<br>GCAGATAACCCCCAGGAATAATATCTCCCTCGTGAAGTTTGGTCC<br>ACATGTCTCTCTGAATGTCTTTTTTGGAAAGCCTGGTCCCTGGTTT<br>GAAGATTGTGAGATGGATGGTGATAAGAACTCCATATTCCATGAC<br>ATTGATGGCTCTGTGACAGGATACAAGGATGCTTATGTGGGAAGA<br>ATGGACAACTACCTGATCCGCCATCCAAGCTGTGTAAATGTGTCTA<br>AGTGGAATGCAGTGATCTGCAGTGGGACCTATGCACAGGTCTATG<br>TACAGACATGGAGCACTCAGAATCTTTCTATGACCATTACACGAG<br>ATGAGTATCCGTCCAACCCTATGGTGCTCCGAGGTATTAATCAGA<br>AGGCTGCCTTTCCACAGTACCAGCCTGTCGTCATGCTGGAGAAGG<br>GTTATACCATCCACTGGAATGGGCCGGCACCACGGACTACATTTCT<br>ATACCTCGTCAACTTCAACAAGAATGACTGGATTCGAGTTGGCCTT<br>TGCTATCCATCAAACACAAGTTTTCAAGTTACCTTTGGCTATTTGC<br>AGCGGCAGAATGGCTCATTATCCAAAATCGAAGAATATGAGCCTG<br>TGCATTCACTGGAAGAACTGCAAAGAAAGCAATCCGAGAGGAAA<br>TTCTATTTTGACTCCAGCACGGGGTTACTGTTTTTGTATCTCAAAG<br>CCAAAAGCCACAGGCATGGCCACAGTTACTGTTCATCTCAGGGAT<br>GTGAAAGAGTCAAGATCCAAGCAGCCACAGACTCAAAGGACATC

	AGTAACTGCATGGCCAAAGCATACCCACAGTACTACAGAAAGCCGTCAGTGGTCAAGCGGATGCCGGCCATGCTCACTGGACTCTGTCAAGGCTGTGGCACTCGGCAGGTGGTGTTTACTAGTGATCCTCATAAAAGTTACCTCCCTGTGCAATTCCAGTCACCTGATAAAGCAGAAACCCAGCGTGGAGACCCGTCTGTTATTTCTGTCAATGGCACTGACTTTACCTTCCGAAGTGCAGGCGTCCTCCTCCTTGTTGTGGATCCGTGCAGCGTTCCATTCCGCTTGACGGAAAAAACGGTTTTTCCTCTTGCTGATGTCAGTCGCATTGAAGAGTATTTAAAAACAGGCATCCCTCCAAGGTCCATTGTTCTGTTGAGCACAAGAGGAGAAATAAAGCAGTTAAACATTTCACACTTACTAGTACCTCTGGGATTAGCCAAACCAGCTCATCTTTATGACAAAGGGAGTACCATATTTTTGGGATTCAGTGGAAACTTTAAACCATCATGGACTAAGCTATTTACCAGTCCTGCTGGACAGGGCCTTGGGGTGCTTGAACAATTCATACCTTTGCAGCTGGACGAATATGGTTGTCCCAGAGCCACCACTGTCCGCAGAAGAGACCTGGAACTGCTAAAGCAAGCTTCAAAAGCACAT
Flag-TMEM2-K278R	ATGTATGCCACTGATTCCAGGGGACACTCCCCTGCTTTCCTCCAACCTCAGAATGGAAATAGTCGTCACCCATCTGGCTATGTTCCAGGGAAGGTTGTCCCATTGCGTCCCCCTCCTCCTCCAAAGAGTCAAGCTTCAGCCAAATTTACCTCCATCAGACGAGAAGACCGGGCAACCTTCGCATTCTCACCTGAAGAACAGCAAGCCCAGAGAGAAAGTCAAAAGCAAAAGAGACACAAAAATACTTTCATTTGTTTTGCTATTACTAGTTTCTCATTTTTTATTGCACTTGCAATCATTTTAGGAATATCCTCAAAATATGCTCCAGATGAAAATTGCCCAGATCAAAATCCTCGTCTCAGGAATTGGGATCCAGGACAAGATTCTGCAAAGCAAGTTGTTATCAAGGAGGGAGATATGCTCCGTCTGACCTCAGACGCCACCGTGCATTCTATAGTCATTCAGGATGGAGGACTGCTTGTATTTGGGGACAATAAAGATGGATCCAGAAATATTACTTTGAGGACTCATTACATCCTGATCCAGGATGGTGGGGCGCTTCATATTGGAGCAGAAAAATGCCGCTATAAATCCAAAGCGACAATTACCTTGTATGGCAAGTCAGATGAAGGTGAAAGTATGCCAACATTTGGCAAAAAGTTTATTGGTGTGGAAGCTGGCGGGACACTGGAGTTACATGGGGCACGGAAGGCATCGTGGACGTTGTTGGCAAGGACCCTGAATTCCTCAGGCTTGCCCTTTGGGTCCTATACCTTTGAAAAGGACTTTTCCCGGGGCCTCAATGTGAGGGTCATTGACCCAAGACACGGCCAGAATTTTGGAAAGTGAGAGATTTGATACCCATGAATACCGCAATGAGAGCAGGCGGCTTCAGGAGTTTCTGAGATTCCAGGATCCAGGTCGGATTGTTGCCATAGCTGTCGGGGATTCAGCCGCTAAAAGTCTCTTACAAGGAACCATCCAGATGATCCAGGAACGGTTGGGAAGTGAACTGATCCAAGGACTGGGCTACAGGCAAGCTTGGGCTTTAGTTGGTGTCATTGATGGTGGAAGCACTTCTTGCAATGAATCCGTGAGAAACTATGAAAATCATAGCAGTGGCGGGAAGGCTCTTGCCCAAAGAGAATTTTATACTGTGGATGGCCAGAAGTTCTCTGTGACAGCTTATAGTGAATGGATTGAAGGCGTTTCTCTTTCAGGATTCCGGGTAGAGGTTGTAGATGGAGTGAAGCTAAATTTGCTAGATGATGTTAGTAGTTGGAAACCTGGAGACCAGATTGTGGTCGCAAGCACAG

	ACTATTCCATGTACCAAGCAGAGGAGTTCACTCTTCTTCCCTGTTC<br>TGAATGCAGCCATTTTCAGGTCAAAGTCAAAGAAACCCCTCAGTT<br>CCTGCACATGGGTGAGATCATAGACGGTGTAGACATGAGAGCTGA<br>GGTTGGAATTCTTACCCGGAATATTGTGATCCAAGGAGAAGTGGA<br>GGACTCATGCTACGCAGAAAATCAGTGCCAATTTTTTGATTATGAT<br>ACCTTTGGGGGACACATTATGATAATGAAAAATTTTACTTCAGTCC<br>ATCTTTCTTATGTGGAATTGAAACACATGGGTCAGCAGCAGATGG<br>GGCGATACCCTGTTCATTTTCACCTGTGTGGTGACGTGGATTATAA<br>AGGAGGATACAGACATGCAACATTTGTGGACGGCCTGTCTATTCA<br>TCACAGCTTCTCAAGGTGCATCACTGTGCATGGGACAAATGGCTT<br>GCTAATAAAAGACACCATTGGGTTTGACACACTAGGTCATTGTTTC<br>TTTTTGGAAGATGGTATTGAACAGAGGAATACTTTGTTCCACAATC<br>TGGGACTCCTCACCAAGCCGGGTACTCTCCTGCCCACCGATAGGA<br>ACAACTCCATGTGTACCACCATGCGAGATAAAGTGTTTGGAAATT<br>ACATTCCTGTGCCTGCTACTGACTGTATGGCTGTTTCAACTTTCTG<br>GATTGCTCATCCCAACAATAATCTGATTAATAATGCAGCTGCAGG<br>CTCACAGGATGCTGGAATATGGTATTTATTCCACAAGGAACCAAC<br>TGGGGAATCCAGTGGATTGCAGCTCTTGGCAAAACCAGAACTCAC<br>TCCATTGGGTATATTTTATAACAACAGGGTCCATTCAAATTTTAAG<br>GCTGGCTTATTTATTGACAAAGGTGTCAAAACAACCAACTCTAGT<br>GCTGCTGACCCAAGGGAATACCTCTGTTTGGACAATAGTGCAAGA<br>TTTCGACCTCATCAGGATGCAAACCCCGAAAAACCACGTGTTGCT<br>GCTCTAATTGACAGGCTCATTGCTTTTAAAAATAATGATAATGGAG<br>CTTGGGTCAGAGGAGGAGATATTATCGTTCAAAATTCAGCATTTG<br>CAGATAATGGAATAGGACTGACCTTTGCCAGTGATGGAAGCTTCC<br>CAAGTGATGAAGGTTCCAGCCAAGAGGTATCTGAATCTCTCTTTGT<br>TGGGGAGAGCAGGAATTACGGCTTTCAGGGTGGTCAGAACAAGTA<br>TGTAGGCACTGGAGGAATAGACCAGAAGCCTCGAACATTACCCAG<br>GAACAGGACGTTCCCAATTAGAGGCTTTCAGATTTATGATGGGCC<br>CATTCATCTCACAAGGAGCACTTTCAAAAAATATGTGCCAACTCC<br>AGATAGGTACAGCAGTGCAATTGGCTTCCTCATGAAGAATTCCTG<br>GCAGATAACCCCCAGGAATAATATCTCCCTCGTGAAGTTTGGTCC<br>ACATGTCTCTCTGAATGTCTTTTTTGGAAAGCCTGGTCCCTGGTTT<br>GAAGATTGTGAGATGGATGGTGATAAGAACTCCATATTCCATGAC<br>ATTGATGGCTCTGTGACAGGATACAAGGATGCTTATGTGGGAAGA<br>ATGGACAACTACCTGATCCGCCATCCAAGCTGTGTAAATGTGTCTA<br>AGTGGAATGCAGTGATCTGCAGTGGGACCTATGCACAGGTCTATG<br>TACAGACATGGAGCACTCAGAATCTTTCTATGACCATTACACGAG<br>ATGAGTATCCGTCCAACCCTATGGTGCTCCGAGGTATTAATCAGA<br>AGGCTGCCTTTCCACAGTACCAGCCTGTCGTCATGCTGGAGAAGG<br>GTTATACCATCCACTGGAATGGGCCGGCACCACGGACTACATTTCT<br>ATACCTCGTCAACTTCAACAAGAATGACTGGATTCGAGTTGGCCTT<br>TGCTATCCATCAAACACAAGTTTTCAAGTTACCTTTGGCTATTTGC<br>AGCGGCAGAATGGCTCATTATCCAAAATCGAAGAATATGAGCCTG

	TGCATTCACTGGAAGAACTGCAAAGAAAGCAATCCGAGAGGAAA TTCTATTTTGACTCCAGCACGGGGTTACTGTTTTTGTATCTCAAAG CCAAAAGCCACAGGCATGGCCACAGTTACTGTTCATCTCAGGGAT GTGAAAGAGTCAAGATCCAAGCAGCCACAGACTCAAAGGACATC AGTAACTGCATGGCCAAAGCATACCCACAGTACTACAGAAAGCCG TCAGTGGTCAAGCGGATGCCGGCCATGCTCACTGGACTCTGTCAA GGCTGTGGCACTCGGCAGGTGGTGTTTACTAGTGATCCTCATAAA AGTTACCTCCCTGTGCAATTCCAGTCACCTGATAAAGCAGAAACC CAGCGTGGAGACCCGTCTGTTATTTCTGTCAATGGCACTGACTTTA CCTTCCGAAGTGCAGGCGTCCTCCTCCTTGTTGTGGATCCGTGCAG CGTTCCATTCCGCTTGACGGAAAAAACGGTTTTTCCTCTTGCTGAT GTCAGTCGCATTGAAGAGTATTTAAAAACAGGCATCCCTCCAAGG TCCATTGTTCTGTTGAGCACAAGAGGAGAAATAAAGCAGTTAAAC ATTTCACACTTACTAGTACCTCTGGGATTAGCCAAACCAGCTCATC TTTATGACAAAGGGAGTACCATATTTTTGGGATTCAGTGGAAACTT TAAACCATCATGGACTAAGCTATTTACCAGTCCTGCTGGACAGGG CCTTGGGGTGCTTGAACAATTCATACCTTTGCAGCTGGACGAATAT GGTTGTCCCAGAGCCACCACTGTCCGCAGAAGAGACCTGGAACTG CTAAAGCAAGCTTCAAAAGCACAT
Flag-TMEM2-K1153R	ATGTATGCCACTGATTCCAGGGGACACTCCCCTGCTTTCCTCCAAC CTCAGAATGGAAATAGTCGTCACCCATCTGGCTATGTTCCAGGGA AGGTTGTCCCATTGCGTCCCCCTCCTCCTCCAAAGAGTCAAGCTTC AGCCAAATTTACCTCCATCAGACGAGAAGACCGGGCAACCTTCGC ATTCTCACCTGAAGAACAGCAAGCCCAGAGAGAAAGTCAAAAGC AAAAGAGACACAAAAATACTTTCATTTGTTTTGCTATTACTAGTTT CTCATTTTTTATTGCACTTGCAATCATTTTAGGAATATCCTCAAAAT ATGCTCCAGATGAAAATTGCCCAGATCAAAATCCTCGTCTCAGGA ATTGGGATCCAGGACAAGATTCTGCAAAGCAAGTTGTTATCAAGG AGGGAGATATGCTCCGTCTGACCTCAGACGCCACCGTGCATTCTAT AGTCATTCAGGATGGAGGACTGCTTGTATTTGGGGACAATAAAGA TGGATCCAGAAATATTACTTTGAGGACTCATTACATCCTGATCCAG GATGGTGGGGCGCTTCATATTGGAGCAGAAAAATGCCGCTATAAA TCCAAAGCGACAATTACCTTGTATGGCAAGTCAGATGAAGGTGAA AGTATGCCAACATTTGGCAAAAAGTTTATTGGTGTGGAAGCTGGC GGGACACTGGAGTTACATGGGGCACGGAAGGCATCGTGGACGTTG TTGGCAAGGACCCTGAATTCCTCAGGCTTGCCCTTTGGGTCCTATA CCTTTGAAAAGGACTTTTCCCGGGGCCTCAATGTGAGGGTCATTGA CCAAGACACGGCCAAAATTTTGGAAAGTGAGAGATTTGATACCCA TGAATACCGCAATGAGAGCAGGCGGCTTCAGGAGTTTCTGAGATT CCAGGATCCAGGTCGGATTGTTGCCATAGCTGTCGGGGATTCAGC CGCTAAAAGTCTCTTACAAGGAACCATCCAGATGATCCAGGAACG GTTGGGAAGTGAACTGATCCAAGGACTGGGCTACAGGCAAGCTTG GGCTTTAGTTGGTGTCATTGATGGTGGAAGCACTTCTTGCAATGAA TCCGTGAGAAACTATGAAAATCATAGCAGTGGCGGGAAGGCTCTT

	GCCCAAAGAGAATTTTATACTGTGGATGGCCAGAAGTTCTCTGTG<br>ACAGCTTATAGTGAATGGATTGAAGGCGTTTCTCTTTCAGGATTCC<br>GGGTAGAGGTTGTAGATGGAGTGAAGCTAAATTTGCTAGATGATG<br>TTAGTAGTTGGAAACCTGGAGACCAGATTGTGGTCGCAAGCACAG<br>ACTATTCCATGTACCAAGCAGAGGAGTTCACTCTTCTTCCCTGTTC<br>TGAATGCAGCCATTTTCAGGTCAAAGTCAAAGAAACCCCTCAGTT<br>CCTGCACATGGGTGAGATCATAGACGGTGTAGACATGAGAGCTGA<br>GGTTGGAATTCTTACCCGGAATATTGTGATCCAAGGAGAAGTGGA<br>GGACTCATGCTACGCAGAAAATCAGTGCCAATTTTTTGATTATGAT<br>ACCTTTGGGGGACACATTATGATAATGAAAAATTTTACTTCAGTCC<br>ATCTTTCTTATGTGGAATTGAAACACATGGGTCAGCAGCAGATGG<br>GGCGATACCCTGTTCATTTTCACCTGTGTGGTGACGTGGATTATAA<br>AGGAGGATACAGACATGCAACATTTGTGGACGGCCTGTCTATTCA<br>TCACAGCTTCTCAAGGTGCATCACTGTGCATGGGACAAATGGCTT<br>GCTAATAAAAGACACCATTGGGTTTGACACACTAGGTCATTGTTTC<br>TTTTTGGAAGATGGTATTGAACAGAGGAATACTTTGTTCCACAATC<br>TGGGACTCCTCACCAAGCCGGGTACTCTCCTGCCCACCGATAGGA<br>ACAACTCCATGTGTACCACCATGCGAGATAAAGTGTTTGGAAATT<br>ACATTCCTGTGCCTGCTACTGACTGTATGGCTGTTTCAACTTTCTG<br>GATTGCTCATCCCAACAATAATCTGATTAATAATGCAGCTGCAGG<br>CTCACAGGATGCTGGAATATGGTATTTATTCCACAAGGAACCAAC<br>TGGGGAATCCAGTGGATTGCAGCTCTTGGCAAAACCAGAACTCAC<br>TCCATTGGGTATATTTTATAACAACAGGGTCCATTCAAATTTTAAG<br>GCTGGCTTATTTATTGACAAAGGTGTCAAAACAACCAACTCTAGT<br>GCTGCTGACCCAAGGGAATACCTCTGTTTGGACAATAGTGCAAGA<br>TTTCGACCTCATCAGGATGCAAACCCCGAAAAACCACGTGTTGCT<br>GCTCTAATTGACAGGCTCATTGCTTTTAAAAATAATGATAATGGAG<br>CTTGGGTCAGAGGAGGAGATATTATCGTTCAAAATTCAGCATTTG<br>CAGATAATGGAATAGGACTGACCTTTGCCAGTGATGGAAGCTTCC<br>CAAGTGATGAAGGTTCCAGCCAAGAGGTATCTGAATCTCTCTTTGT<br>TGGGGAGAGCAGGAATTACGGCTTTCAGGGTGGTCAGAACAAGTA<br>TGTAGGCACTGGAGGAATAGACCAGAAGCCTCGAACATTACCCAG<br>GAACAGGACGTTCCCAATTAGAGGCTTTCAGATTTATGATGGGCC<br>CATTCATCTCACAAGGAGCACTTTCAAAAAATATGTGCCAACTCC<br>AGATAGGTACAGCAGTGCAATTGGCTTCCTCATGAAGAATTCCTG<br>GCAGATAACCCCCAGGAATAATATCTCCCTCGTGAAGTTTGGTCC<br>ACATGTCTCTCTGAATGTCTTTTTTGGAAAGCCTGGTCCCTGGTTT<br>GAAGATTGTGAGATGGATGGTGATAAGAACTCCATATTCCATGAC<br>ATTGATGGCTCTGTGACAGGATACAAGGATGCTTATGTGGGAAGA<br>ATGGACAACTACCTGATCCGCCATCCAAGCTGTGTAAATGTGTCTA<br>AGTGGAATGCAGTGATCTGCAGTGGGACCTATGCACAGGTCTATG<br>TACAGACATGGAGCACTCAGAATCTTTCTATGACCATTACACGAG<br>ATGAGTATCCGTCCAACCCTATGGTGCTCCGAGGTATTAATCAGA<br>AGGCTGCCTTTCCACAGTACCAGCCTGTCGTCATGCTGGAGAAGG

	GTTATACCATCCACTGGAATGGGCCGGCACCACGGACTACATTTCT ATACCTCGTCAACTTCAACAAGAATGACTGGATTCGAGTTGGCCTT TGCTATCCATCAAACACAAGTTTTCAAGTTACCTTTGGCTATTTGC AGCGGCAGAATGGCTCATTATCCAAAATCGAAGAATATGAGCCTG TGCATTCACTGGAAGAACTGCAAAGAAAGCAATCCGAGAGGAAA TTCTATTTTGACTCCAGCACGGGGTTACTGTTTTTGTATCTCAAAG CCAAAAGCCACAGGCATGGCCACAGTTACTGTTCATCTCAGGGAT GTGAAAGAGTCAGGATCCAAGCAGCCACAGACTCAAAGGACATC AGTAACTGCATGGCCAAAGCATACCCACAGTACTACAGAAAGCCG TCAGTGGTCAAGCGGATGCCGGCCATGCTCACTGGACTCTGTCAA GGCTGTGGCACTCGGCAGGTGGTGTTTACTAGTGATCCTCATAAA AGTTACCTCCCTGTGCAATTCCAGTCACCTGATAAAGCAGAAACC CAGCGTGGAGACCCGTCTGTTATTTCTGTCAATGGCACTGACTTTA CCTTCCGAAGTGCAGGCGTCCTCCTCCTTGTTGTGGATCCGTGCAG CGTTCCATTCCGCTTGACGGAAAAAACGGTTTTTCCTCTTGCTGAT GTCAGTCGCATTGAAGAGTATTTAAAAACAGGCATCCCTCCAAGG TCCATTGTTCTGTTGAGCACAAGAGGAGAAATAAAGCAGTTAAAC ATTTCACACTTACTAGTACCTCTGGGATTAGCCAAACCAGCTCATC TTTATGACAAAGGGAGTACCATATTTTTGGGATTCAGTGGAAACTT TAAACCATCATGGACTAAGCTATTTACCAGTCCTGCTGGACAGGG CCTTGGGGTGCTTGAACAATTCATACCTTTGCAGCTGGACGAATAT GGTTGTCCCAGAGCCACCACTGTCCGCAGAAGAGACCTGGAACTG CTAAAGCAAGCTTCAAAAGCACAT
HA-Ub	ATGCAGATCTTCGTGAAAACCCTTACCGGCAAGACCATCACCCTT GAGGTGGAGCCCAGTGACACCATCGAAAATGTGAAGGCCAAGAT CCAGGATAAGGAAGGCATTCCCCCCGACCAGCAGAGGCTCATCTT TGCAGGCAAGCAGCTGGAAGATGGCCGTACTCTTTCTGACTACAA CATCCAGAAGGAGTCGACCCTGCACCTGGTCCTGCGTCTGAGAGG TGGT
HA-Ub-K48	ATGCAGATCTTCGTGAGAACCCTTACCGGCAGGACCATCACCCTT GAGGTGGAGCCCAGTGACACCATCGAAAATGTGAGGGCCAGGAT CCAGGATAGGGAAGGCATTCCCCCCGACCAGCAGAGGCTCATCTT TGCAGGCAAGCAGCTGGAAGATGGCCGTACTCTTTCTGACTACAA CATCCAGAGGGAGTCGACCCTGCACCTGGTCCTGCGTCTGAGAGG TGGT
HA-Ub-K63	ATGCAGATCTTCGTGAGAACCCTTACCGGCAGGACCATCACCCTT GAGGTGGAGCCCAGTGACACCATCGAAAATGTGAGGGCCAGGAT CCAGGATAGGGAAGGCATTCCCCCCGACCAGCAGAGGCTCATCTT TGCAGGCAGGCAGCTGGAAGATGGCCGTACTCTTTCTGACTACAA CATCCAGAAGGAGTCGACCCTGCACCTGGTCCTGCGTCTGAGAGG TGGT

Supplementary Material 3

Gene_ID	M_S1	M_S2	M_S3	M1	M2	M3	log2FoldCh	pvalue
gene-ABCA	12.63812	12.89932	12.8279	11.21795	11.28745	11.29259	-1.52668	2.01E-73
gene-ACAP	5.842223	5.936132	3.366073	1.021046	3.523876	2.418884	-2.98828	0.001842
gene-ACKR	6.426812	6.139438	6.341135	7.47618	7.199523	7.520406	1.108081	4.47E-05
gene-ACOX	8.373197	8.337418	8.14049	7.063841	7.147037	7.45041	-1.06281	6.26E-07
gene-ACRV	5.257821	6.020922	5.542955	3.945932	4.389206	3.919302	-1.59819	0.003152
gene-ACTA	7.094553	7.785305	8.101897	5.467096	5.797351	5.509778	-2.14325	8.23E-10
gene-ACTN	6.274892	6.176874	7.373297	4.499348	5.316843	5.761757	-1.46027	0.003712
gene-ADAN	9.524885	9.367926	8.933341	10.55154	10.44681	10.521	1.212226	3.23E-15
gene-ADGI	7.489158	7.710692	7.590967	6.699593	6.444941	6.658128	-1.00222	4.74E-05
gene-AKR1	4.743968	5.673443	6.183735	4.039213	3.765853	0	-2.39132	0.007747
gene-ALDH	5.293404	5.140939	6.27329	4.432145	0	3.10572	-2.39794	0.010172
gene-ALOX	7.511992	8.58377	9.137932	6.326664	5.850837	5.790371	-2.56491	3.08E-11
gene-AMEI	8.027215	8.324966	8.548281	6.874319	6.977048	7.689458	-1.0941	9.42E-05
gene-AMH	11.72946	12.05211	12.04419	10.26455	10.34447	10.3716	-1.62287	8.21E-53
gene-ANGI	6.001304	6.632542	6.718088	5.795914	5.068574	4.574389	-1.27066	0.006065
gene-ANKD	7.541886	7.437513	7.810296	6.190957	6.756563	6.440695	-1.13296	4.80E-05
gene-ANKS	6.649097	7.896076	4.294358	3.945932	5.797351	4.574389	-1.95141	0.010274
gene-ANO9	10.10169	9.832638	9.987174	11.0169	11.10275	10.80576	1.003385	2.13E-17
gene-AOC3	8.515587	8.945504	8.59766	7.450939	7.699741	7.726948	-1.07182	1.34E-07
gene-APLN	6.519874	7.468034	7.512219	4.795151	5.850837	6.000896	-1.61528	0.000196
gene-AQP1	11.13268	11.16268	11.16789	10.08067	10.18961	10.13969	-1.0179	2.74E-35
gene-ARHG	6.105071	5.102546	6.048215	7.096763	6.728261	6.673571	1.040455	0.00712
gene-ARHG	6.32733	6.535006	7.237473	5.467096	5.927523	5.790371	-1.02809	0.008694
gene-ARHG	7.951279	8.26104	8.356699	7.326928	7.299092	6.860598	-1.02688	1.48E-05
gene-ASAH	8.41418	8.1665	8.106778	8.201517	10.15415	10.12993	1.501439	0.009596
gene-ATP1	7.761056	7.512639	7.936289	6.641278	6.298631	6.748401	-1.18062	8.28E-06
gene-BAAT	7.956838	8.479019	8.116491	7.298908	7.39223	6.860598	-1.00358	6.38E-05
gene-BHLH	8.882714	9.265219	9.327571	7.868157	7.805119	8.228316	-1.19584	1.68E-09
gene-BTBD	5.065416	4.893574	5.147699	6.170484	7.383182	7.319097	2.047858	1.78E-06
gene-C1orf	5.690344	5.8691	5.829451	4.795151	4.711826	4.507024	-1.15275	0.010353
gene-C3	12.44028	12.66933	12.61744	11.14637	11.09237	11.17815	-1.44006	3.86E-69
gene-C9orf	6.805151	7.203609	7.02701	5.795914	4.15413	4.974208	-1.924	1.96E-05
gene-CABP	5.427559	6.041363	6.467943	4.795151	3.649932	5.07068	-1.4561	0.008726
gene-CALM	0	3.897133	3.078708	4.89835	5.902412	5.951075	2.794929	0.001621
gene-CASS4	6.676307	7.212649	7.02701	4.126828	5.528595	5.9762	-1.61524	0.000642
gene-CATS	5.221338	5.102546	4.900298	2.843248	4.066412	3.277262	-1.68605	0.011559
gene-CCDC	7.519524	7.577062	7.246347	5.795914	7.012686	5.9762	-1.09677	0.003167
gene-CCDC	7.917471	7.350088	8.00064	8.607735	8.808664	9.014785	1.039667	4.03E-06
gene-CEMI	12.56153	12.51938	12.34922	13.63728	13.69026	13.58027	1.157224	5.06E-56
gene-CFAP	7.549264	8.299734	8.34848	6.363203	5.797351	6.347489	-1.93193	6.99E-10
gene-CFAP	6.426812	6.935267	6.768241	4.56356	5.113034	4.816206	-1.91479	1.07E-06
gene-CHI3I	11.25222	10.89989	10.65691	12.38968	12.38587	12.44465	1.450326	5.89E-29
gene-COL2	3.845582	4.41853	4.150502	5.655659	5.741804	5.439251	1.513718	0.003087
gene-COL4	8.953931	8.97768	8.851376	7.874499	7.845205	7.704571	-1.1218	3.20E-12

gene-CORI	5.957614	5.564672	5.829451	4.209425	4.527024	3.919302	-1.60414	0.001261
gene-CTSK	8.923028	9.28892	9.154526	7.809785	8.10939	8.044547	-1.13998	1.36E-10
gene-CUBN	9.448031	10.24214	10.16267	7.822962	7.770841	8.358232	-1.98788	4.63E-17
gene-DCAF	6.85363	6.924302	6.942167	5.433126	4.875621	5.365099	-1.68867	1.10E-06
gene-DEPP	10.79301	10.38993	10.5145	11.87783	11.71457	11.66175	1.179274	1.06E-23
gene-DGKI	5.979625	6.430394	6.048215	2.355456	3.973015	3.695528	-2.79096	1.08E-06
gene-DNAH	12.03522	12.2517	12.33889	10.22787	10.19479	10.48644	-1.90624	4.58E-65
gene-DNAH	6.410701	6.300742	6.483039	4.361657	5.198043	5.246193	-1.44251	0.000616
gene-DNAS	6.144574	5.749957	6.10786	7.170779	7.299092	6.528243	1.037525	0.002638
gene-DRD2	11.46708	11.87267	11.97547	10.71331	10.67339	10.64271	-1.11163	1.86E-18
gene-DRP2	5.912559	6.000187	6.718088	3.8462	4.066412	2.910993	-2.66456	1.27E-06
gene-ENPE	8.058574	8.080223	7.780059	8.954594	8.94095	9.324585	1.108176	2.97E-09
gene-ENTP	9.848666	9.778871	9.597532	10.9013	10.79606	10.77209	1.079807	2.69E-24
gene-EXOC	6.292584	6.711197	6.570436	7.541408	7.74282	7.704571	1.138392	6.72E-06
gene-F5	6.8655	7.239436	7.340521	5.595502	5.741804	6.404132	-1.21905	0.000466
gene-FAM1	5.912559	7.221634	6.373897	4.847673	4.527024	5.951075	-1.3884	0.009355
gene-FAM2	6.549608	7.341871	5.964646	5.923735	4.823062	5.204276	-1.35898	0.005784
gene-FAM2	6.57874	7.437513	6.10786	5.995322	5.022701	5.204276	-1.36253	0.004335
gene-FAM7	6.649097	6.367024	4.854601	7.588464	7.035965	7.248668	1.179372	0.007896
gene-FBXO	11.23459	10.79921	11.27096	9.442608	10.16475	9.581525	-1.35309	9.16E-13
gene-FBXW	4.890577	6.120349	7.136076	2.355456	0	0	-5.82332	2.50E-08
gene-FGA	12.89313	13.31694	13.42102	11.51142	11.57949	11.58777	-1.66822	4.89E-44
gene-FGF1	4.890577	4.41853	3.990696	6.063524	6.069654	5.672342	1.51085	0.001908
gene-FGF1	4.890577	4.594836	4.425162	6.149717	6.15718	5.761757	1.422044	0.001381
gene-FGG	13.28225	13.58045	13.75544	12.06241	12.2759	12.18963	-1.37399	3.27E-34
gene-FLAC	5.690344	6.723899	6.341135	4.209425	5.461406	4.870819	-1.40719	0.005518
gene-FNDC	5.36204	4.893574	5.513852	3.623316	3.649932	3.695528	-1.70485	0.004068
gene-FOXD	5.065416	5.64701	5.805633	3.036632	3.523876	3.695528	-2.20487	0.00026
gene-FZD4	14.0142	14.05602	14.19381	12.97529	13.02656	12.91887	-1.11591	6.48E-61
gene-GADL	7.541886	7.717059	7.405345	6.782872	6.624587	5.116601	-1.22656	0.002527
gene-GARN	10.35	10.5258	10.64102	9.559403	9.378963	9.518782	-1.02386	1.68E-17
gene-GATA	6.344394	6.79785	6.007035	5.433126	4.823062	4.816206	-1.39142	0.001442
gene-GGT5	4.637237	3.484463	3.711928	6.326664	6.023816	6.385497	2.292629	5.98E-06
gene-GJA5	7.418403	6.535006	7.02701	8.26082	8.204914	8.441777	1.275144	1.34E-06
gene-GNL1	6.474093	6.445811	5.985997	4.847673	5.022701	5.402651	-1.23407	0.002704
gene-GNL1	6.504775	6.505883	6.048215	4.89835	5.113034	5.402651	-1.24017	0.001988
gene-GON7	6.900534	7.333607	6.96385	8.047254	8.337295	8.044547	1.076084	3.49E-06
gene-GRAN	9.475398	9.347551	9.574814	7.181049	6.964971	6.80559	-2.48503	3.53E-45
gene-H2BC	6.344394	6.988873	6.840346	5.532727	4.768515	5.845952	-1.32171	0.001459
gene-H3C1	4.691589	5.102546	5.422846	3.359593	2.053372	3.430557	-2.17416	0.002797
gene-H3C1	6.064455	5.8691	5.653828	3.359593	3.765853	3.569118	-2.39983	6.13E-06
gene-H3C4	6.649097	4.479724	6.405932	7.416581	7.488182	7.799127	1.479833	0.001679
gene-H4C1	6.043711	4.479724	6.255818	6.796296	7.383182	6.658128	1.218257	0.011123
gene-HCLS	5.520575	6.176874	6.220226	3.945932	5.198043	3.695528	-1.61187	0.004905
gene-HPX	7.973386	8.299734	8.580218	7.007245	6.864527	7.603381	-1.11666	5.25E-05

gene-HSD1	7.07424	6.924302	6.792679	4.361657	5.022701	5.672342	-1.8517	8.35E-06
gene-IER3	5.742767	6.47616	6.007035	7.407862	7.269936	7.227898	1.207635	9.41E-05
gene-IFFO1	4.580757	4.751924	4.361242	5.971852	6.069654	6.162855	1.536757	0.000384
gene-IFITM	9.294063	9.488207	9.59058	8.31312	8.152627	8.212544	-1.23792	3.70E-16
gene-IQCN	6.292584	6.101004	6.390003	3.945932	4.315023	4.759445	-1.94225	1.89E-05
gene-ITGAI	5.490231	6.723899	6.357609	5.500285	4.236818	4.200872	-1.52866	0.006419
gene-ITPK1	10.58351	10.79996	10.68411	12.34356	11.16873	12.23425	1.312072	0.000248
gene-IZUM	7.318318	6.988873	7.15505	8.308443	8.157942	8.15869	1.055514	3.55E-07
gene-KCNK	6.064455	6.061519	6.048215	4.287548	3.765853	4.112983	-2.05292	1.31E-05
gene-KCNS	5.293404	5.749957	6.679294	4.432145	4.527024	4.283713	-1.65526	0.002425
gene-KIF26	10.16549	10.38394	10.35545	9.280126	9.030596	9.439701	-1.04652	5.06E-14
gene-KIF26	10.08275	10.40677	10.40681	9.243844	8.959331	9.279879	-1.14024	4.37E-15
gene-KLF2	7.094553	6.956949	7.541354	6.345049	5.495392	6.328103	-1.12463	0.001522
gene-KLHD	5.935262	5.724901	5.029295	7.063841	6.624587	6.913586	1.284655	0.000587
gene-KLHL:	5.866051	6.158277	6.390003	3.359593	0	2.910993	-3.73656	2.56E-07
gene-KRT3	5.32813	5.102546	5.32571	3.359593	2.644145	3.569118	-2.13803	0.000877
gene-KRT4	8.296359	8.844434	8.566111	7.789791	6.928119	7.367378	-1.18655	8.02E-06
gene-LAMI	5.690344	5.250344	5.852882	3.497472	4.768515	4.112983	-1.4515	0.010991
gene-LCT	6.841662	6.520518	7.272647	5.848409	5.426601	5.872959	-1.19698	0.00096
gene-LGAL:	6.394407	4.980828	5.805633	7.052698	6.609149	7.459347	1.258764	0.003618
gene-LGAL:	5.221338	6.736491	6.452689	5.25013	3.523876	4.816206	-1.61244	0.007486
gene-LOC1	4.521975	3.897133	4.80741	5.795914	5.741804	5.577017	1.291136	0.010192
gene-LOC1	6.274892	6.879591	7.315441	5.873957	5.461406	6.095657	-1.06494	0.007155
gene-LOC1	6.361258	6.988873	7.3974	5.873957	5.461406	6.095657	-1.15775	0.003064
gene-LOC1	7.352457	6.83345	6.875091	6.08556	5.495392	5.509778	-1.33125	0.000162
gene-LRRC	6.923426	6.8909	6.96385	4.795151	5.684034	6.162855	-1.29368	0.001351
gene-MAM	6.32733	5.416052	6.390003	7.354414	6.851465	7.130542	1.030384	0.004476
gene-MBL2	5.663401	5.536147	5.453823	3.945932	0	3.430557	-2.51013	0.0032
gene-MINA	4.185677	3.897133	3.366073	4.795151	5.876855	5.509778	1.676088	0.005816
gene-MMP	5.866051	4.980828	5.985997	3.945932	4.236818	3.81175	-1.7291	0.002174
gene-MRC:	6.084906	6.445811	6.255818	3.497472	3.765853	4.507024	-2.35959	1.75E-06
gene-MRN	7.989746	7.645424	7.405345	9.086106	9.087408	9.077079	1.388033	3.96E-13
gene-MSS5	6.144574	6.195234	7.02701	5.25013	5.528595	5.402651	-1.13628	0.006549
gene-MT1X	6.474093	5.699402	5.920974	7.279922	7.488182	7.528922	1.381062	9.63E-06
gene-MT2A	6.32733	6.821681	6.127206	8.619111	8.710805	8.631646	2.211255	6.55E-21
gene-MUC	7.8593	8.12879	7.761608	6.822775	6.259612	5.790371	-1.58268	4.13E-07
gene-MUC	6.607296	7.00977	7.057582	4.209425	5.797351	5.872959	-1.46065	0.0017
gene-MUC	5.32813	5.699402	4.758623	3.945932	3.649932	3.81175	-1.57465	0.008789
gene-MX2	8.148757	8.800138	8.913978	4.847673	5.198043	5.702767	-3.40206	6.77E-27
gene-MYH:	6.792772	6.231267	6.255818	5.21057	5.528595	5.577017	-1.02024	0.008243
gene-MYO:	6.877273	7.221634	7.192265	5.564456	5.495392	5.02325	-1.74829	2.32E-07
gene-NBPF	11.12159	11.45038	11.49505	10.38161	10.21788	10.47327	-1.00431	3.26E-16
gene-NBPF	6.549608	6.367024	6.405932	4.039213	5.713208	3.919302	-1.67017	0.002595
gene-NBPF	6.220476	6.83345	5.599456	3.623316	3.765853	3.10572	-2.89027	3.01E-07
gene-NBPF	6.394407	6.711197	6.584501	4.994657	4.975321	5.402651	-1.45881	0.000103

gene-NDNF	9.086614	9.052598	9.408863	7.634033	8.17377	8.026619	-1.23331	5.75E-10
gene-NEUF	7.220045	7.212649	6.953049	8.134396	8.098375	8.512184	1.132298	5.07E-07
gene-NEUF	9.073882	9.497484	9.555308	8.058439	8.471408	8.428185	-1.06289	3.28E-08
gene-NIBAI	8.168054	7.366382	7.698343	8.975396	8.75356	8.643458	1.019361	1.57E-05
gene-NIBAI	5.395172	5.140939	5.453823	6.128647	5.976473	7.920592	1.654259	0.002573
gene-NID2	6.564247	6.79785	6.556232	5.288633	5.769845	5.509778	-1.12503	0.001328
gene-NLGN	9.141339	9.109367	9.182537	8.17607	8.28482	7.611422	-1.09406	4.27E-08
gene-NLRC	6.504775	7.265735	6.639429	5.398337	5.354364	5.509778	-1.44262	0.000121
gene-NLRP	9.448031	9.196249	6.405932	6.433617	5.769845	6.777279	-2.46435	0.003516
gene-NR4A	8.215198	8.26104	9.184847	7.634033	7.419038	7.269144	-1.18272	5.21E-05
gene-NUTN	8.094318	8.661758	8.101897	7.270334	7.157688	7.085071	-1.14374	2.21E-06
gene-PDE2	5.957614	6.47616	6.497977	5.433126	5.528595	4.923439	-1.03067	0.011517
gene-PERN	6.274892	6.54935	5.185102	4.795151	3.873149	3.695528	-1.96266	0.000796
gene-PGBD	4.025628	3.599212	1.517096	5.362688	6.728261	3.695528	2.476238	0.006181
gene-PI16	5.842223	5.564672	5.22156	6.835835	6.069654	6.791504	1.057134	0.007867
gene-PILRA	5.023652	6.101004	5.453823	4.209425	4.45976	3.919302	-1.43153	0.011272
gene-PKD1	6.504775	7.050677	7.381376	6.211143	5.461406	4.574389	-1.47624	0.001559
gene-PLA2(	7.22926	8.278365	8.310905	5.713407	5.495392	6.072544	-2.2605	3.26E-10
gene-PLA2(	8.305102	8.207778	8.69162	7.484497	7.537949	7.174624	-1.01319	7.45E-06
gene-PLXN	4.025628	3.705503	4.294358	5.947994	6.023816	4.816206	1.726541	0.00305
gene-POU2	7.011525	7.090457	7.804299	6.270059	6.593545	5.845952	-1.08927	0.0021
gene-PPOX	8.920186	8.526919	8.810075	10.27418	9.853653	10.08732	1.322452	2.97E-16
gene-PTN	4.637237	4.594836	4.655802	6.308041	5.156164	6.458635	1.487459	0.003714
gene-PTRH	7.68941	7.76701	7.886069	8.912067	8.881151	8.884816	1.113024	3.49E-12
gene-PYGN	9.073882	9.521328	9.781247	6.713811	7.471204	6.748401	-2.4746	1.52E-21
gene-RADII	6.124957	6.399057	6.127206	4.56356	4.315023	3.569118	-2.0736	1.46E-05
gene-RARB	5.716794	5.063103	5.358821	2.843248	3.973015	4.283713	-1.65905	0.008643
gene-RDH1	6.238844	6.773618	7.077609	4.89835	5.654257	5.818429	-1.24803	0.00279
gene-RFTN	5.716794	6.773618	6.666128	4.683998	4.823062	5.02325	-1.64241	0.000333
gene-RGS2	5.145482	6.619005	6.718088	5.169896	5.022701	4.574389	-1.4009	0.008393
gene-RNF2	5.742767	6.430394	6.452689	4.847673	3.385742	2.910993	-2.35399	0.000125
gene-RPGR	6.201871	5.620082	5.731726	4.287548	4.591291	4.638748	-1.40006	0.003337
gene-RSPH	5.32813	5.97915	6.127206	2.619871	3.523876	4.283713	-2.32272	0.000191
gene-RSPH	5.145482	5.592643	6.202096	3.207131	4.15413	3.919302	-1.97943	0.000908
gene-SCAR	6.8655	6.399057	6.679294	5.768933	5.461406	5.641262	-1.04583	0.002641
gene-SCAR	6.989999	7.050677	7.02701	5.971852	5.684034	5.872959	-1.18896	7.06E-05
gene-SCGB	7.442375	5.724901	6.886489	8.34544	7.52155	8.153192	1.209085	0.002791
gene-SCML	7.811014	7.678427	7.761608	6.149717	7.047465	6.642517	-1.10031	0.00014
gene-SCNN	5.817994	5.384398	5.599456	6.398839	7.081425	6.874029	1.217693	0.000742
gene-SEMA	5.793352	5.749957	5.756785	4.126828	4.975321	2.910993	-1.58294	0.00969
gene-SGK1	11.76831	11.95646	11.93419	10.85555	10.82056	10.76219	-1.07583	4.60E-38
gene-SH3R	6.043711	5.798801	6.220226	7.007245	6.903017	7.813139	1.282693	0.00033
gene-SI	8.153605	8.59764	8.785932	7.150016	7.355693	7.414095	-1.23081	1.13E-07
gene-SIRPE	4.32973	4.479724	3.366073	5.713407	6.35525	5.732564	1.891879	0.00044
gene-SKAP	4.580757	4.937861	5.029295	5.923735	5.927523	5.925503	1.089652	0.011136

gene-SLC38	5.427559	5.21479	4.545086	6.018416	6.756563	6.561827	1.39557	0.001357
gene-SLC45	5.32813	5.592643	5.422846	3.623316	2.868193	3.277262	-2.2846	0.000166
gene-SLC5A	6.88895	6.605341	6.666128	5.169896	4.591291	5.439251	-1.64656	2.18E-05
gene-SLC5A	6.57874	6.158277	6.341135	4.947307	4.591291	5.609497	-1.28355	0.003264
gene-SLC8A	6.805151	8.055312	7.908605	5.995322	4.591291	5.204276	-2.3358	2.48E-07
gene-SNX2	2.942601	5.352034	4.545086	5.25013	10.14481	10.05673	5.017933	8.96E-05
gene-SOBP	4.521975	3.897133	4.224222	6.018416	5.068574	5.899471	1.534382	0.004667
gene-SOCS	8.369034	8.548501	8.75828	7.603814	7.346412	7.14169	-1.19526	3.07E-08
gene-SP8	11.61151	11.85726	11.69587	10.15028	10.34097	10.48426	-1.39457	3.44E-36
gene-SRMS	7.8593	8.119207	8.121323	6.270059	6.838284	6.072544	-1.61799	5.68E-09
gene-SRPK	4.025628	2.713598	3.711928	5.433126	5.461406	5.286927	1.905726	0.002066
gene-SULT	7.748291	7.257022	7.555704	8.44681	8.346635	8.974829	1.086412	7.57E-06
gene-SUSD	6.649097	6.577617	6.953049	5.564456	5.592794	5.818429	-1.08869	0.001227
gene-SYT1	5.579413	4.847886	5.22156	6.467576	6.427443	6.833356	1.364858	0.000465
gene-SYTL	7.951279	9.191684	8.381078	7.789791	7.374077	6.673571	-1.2571	0.000459
gene-TCAP	7.585598	7.41419	7.512219	5.971852	6.713899	4.974208	-1.46564	0.000356
gene-TEX1	7.578404	8.212292	8.511946	7.007245	7.000905	6.913586	-1.18305	2.76E-05
gene-TGIF	6.649097	6.879591	7.576965	0	0	0	-9.4458	6.53E-14
gene-TLE6	4.794511	3.804497	4.944591	5.532727	6.092039	6.140802	1.389144	0.006977
gene-TMEN	8.016608	6.334264	7.016673	5.625894	6.046917	5.609497	-1.53033	0.000764
gene-TMEN	3.845582	2.904224	1.923421	4.683998	5.238739	5.246193	2.121361	0.005293
gene-TNS4	8.005924	7.230562	7.06763	9.280126	8.95323	9.233743	1.673337	6.54E-11
gene-TNXB	6.361258	5.798801	6.238132	4.740644	4.823062	5.439251	-1.14175	0.009953
gene-TOR4	9.920119	10.0824	9.941459	8.933487	8.718019	8.864774	-1.14332	1.34E-19
gene-TPH1	5.866051	6.101004	6.068372	4.683998	4.926332	3.695528	-1.53008	0.002167
gene-TRPN	11.2231	11.5974	11.53543	10.02332	10.07067	10.12572	-1.3882	1.90E-33
gene-TTBK	7.900264	8.286951	8.145243	7.1183	6.770509	7.367378	-1.01972	3.46E-05
gene-TUBA	7.000802	6.773618	6.743382	4.847673	5.952206	4.700358	-1.5864	0.000246
gene-TUBB	7.923161	8.207778	8.164097	7.191247	6.928119	7.085071	-1.03704	1.89E-06
gene-UGT1	3.639819	2.493894	4.224222	4.740644	7.546079	5.899471	3.000332	8.06E-05
gene-UTS2	4.890577	3.984178	3.366073	5.128041	6.317752	5.609497	1.605897	0.009928
gene-VPS5	3.524772	3.359792	3.366073	7.052698	2.644145	4.507024	2.449261	0.011243
gene-WDR	4.936313	5.822617	6.048215	7.063841	6.928119	7.248668	1.426859	0.000141
gene-WNT	6.57874	6.38313	6.048215	7.97826	7.401221	7.174624	1.21682	0.000158
gene-ZAP7	4.580757	4.479724	4.708128	5.040502	6.19903	6.328103	1.404051	0.007439
gene-ZNF3	6.064455	5.536147	6.804744	3.359593	4.711826	3.10572	-2.39919	0.000125
gene-ZNF4	5.793352	5.507048	6.666128	3.359593	4.652817	2.68582	-2.35863	0.00036
gene-ZNF4	11.53647	11.63109	11.03528	9.987192	10.15548	10.39973	-1.23371	1.85E-14
gene-ZNF4	7.748291	7.678427	7.504842	4.126828	6.915623	6.528243	-1.3962	0.007881
gene-ZNF7	6.458504	7.879142	7.459771	5.362688	4.389206	4.974208	-2.45466	8.22E-08
new_gene	6.649097	6.54935	6.692342	7.150016	8.001187	7.689458	1.030731	0.000491

padj	Nr	Swiss-Prot	KEGG	gene_onto	eggNOG	Pfam	CAZy	Signal
1.25E-69	5XJY_A^Ch	O95477.3^	K05641|AE	GO:00085	COG1131|	PF12698.6	NA	SignalP-TM
0.01151	NP_05553	Q15027.1^	K12489|AC	GO:00050	COG5347|	PF16746.4	NA	NA
0.000524	NP_00128	O00590.2^	K04187|CC	GO:00199	KOG3656^	PF00001.2	NA	SignalP-TM
1.28E-05	XP_01685	Q9NUZ1.3	NA	GO:00039	COG1960|	PF01756.1	NA	SignalP-TM
0.017688	NP_06445	P26436.2^	NA	GO:00072	NA	PF15851.4	NA	NA
3.49E-08	NP_00109	P68139.1^	K10354|AC	GO:00055	COG5277|	PF00022.1	NA	NA
0.020138	NP_00109	P35609.1^	K05699|AC	GO:00510	COG5069|	PF00435.2	NA	NA
3.82E-13	XP_01150	Q6UY14.2^	NA	GO:00082	KOG3538|	PF05986.1	NA	NA
0.000551	NP_00129	Q96K78.2^	K08464|GI	GO:00049	KOG4193^	PF00002.2	NA	NA
0.036072	1ZUA_X^Cl	O60218.2^	K00011|E1	GO:00081	COG0656|	PF00248.2	NA	NA
0.04484	NP_73957	Q9H2A2.1	NA	GO:00471	COG1012|	PF00171.2	NA	NA
1.75E-09	XP_01688	Q9BYJ1.1^	K18684|AL	GO:00511	NA	PF00305.1	NA	NA
0.000987	NP_00109	Q8N944.2	NA	GO:00080	NA	PF09422.9	NA	NA
1.70E-49	P03971.3^	P03971.3^	K04665|AN	GO:00080	KOG3900^	PF04709.1	NA	NA
0.029578	XP_00114	O15123.1^	K05466|AN	GO:00468	KOG2579^	PF00147.1	NA	NA
0.000556	XP_01686	A6NHY2.3	NA	GO:00071	COG0666|	PF13637.5	NA	NA
0.045201	NP_00133	Q8BIZ1.3^	NA	GO:00468	COG0666|	PF00536.2	NA	NA
3.46E-15	XP_01687	A1A5B4.3^	K19503|AN	GO:00052	KOG2514^	PF04547.1	NA	NA
3.32E-06	XP_01152	Q16853.3^	K00276|AC	GO:00525	COG3733|	PF01179.1	NA	SignalP-TM
0.001808	NP_05910	Q9ULZ1.1^	K05225|AF	GO:00317	NA	PF15360.5	NA	NA
2.44E-32	NP_00135	Q8NBQ7.1	K09870|AC	GO:00152	NA	PF00230.1	NA	SignalP-TM
0.033667	NP_00135	Q6P4F7.2^	NA	GO:00050	KOG2710^	PF00620.2	NA	NA
0.039433	NP_06580	Q2M1Z3.2	NA	GO:00050	NA	PF00620.2	NA	NA
0.0002	NP_00122	Q9NXL2.2^	NA	GO:00050	KOG3519^	PF00621.1	NA	NA
0.042816	XP_01153	Q9NR71.2	K12349|AS	GO:00055	KOG2232^	PF04734.1	NA	SignalP-TM
0.000121	XP_01151	Q4VNC1.3	K14951|AT	GO:00055	KOG0208^	PF00122.1	NA	SignalP-TM
0.000708	NP_00169	Q14032.1^	K00659|BA	GO:00476	COG1073^	PF08840.1	CE7	NA
6.68E-08	AAH25968	Q9C0J9.1^	K03730|BH	GO:00434	KOG4304^	PF00010.2	NA	NA
3.20E-05	NP_00113	C9JJ37.1^R	NA	NA	KOG4350^	PF00651.3	NA	NA
0.045476	XP_00524	O95561.1^	NA	NA	NA	PF15081.5	NA	NA
1.80E-65	NP_00005	P01024.2^	K03990|C3	GO:00317	KOG1366^	PF07678.1	NA	NA
0.000256	NP_00101	Q5JTZ5.2^	NA	NA	NA	PF15733.4	NA	NA
0.039528	XP_00672	Q866X0.1^	NA	GO:00055	COG5126|	PF00036.3	NA	NA
0.010363	NP_61965	Q8TD86.2^	K02183|CA	GO:00055	COG5126|	PF00036.3	NA	NA
0.00486	NP_06508	Q9NQ75.2	NA	GO:19907	NA	PF08824.9	NA	NA
0.049594	NP_07904	Q9H7T0.2^	K16893|CA	GO:00301	NA	PF15149.5	NA	NA
0.017737	NP_00112	Q4G0S7.3^	NA	NA	NA	PF01966.2	NA	NA
6.51E-05	XP_00525	Q9Y6R9.3^	K16755|CC	GO:00058	NA	PF00038.2	NA	NA
1.18E-52	NP_03752	Q9UHN6.1	NA	GO:00452	NA	PF15711.4	NA	NA
3.02E-08	NP_00117	Q8TBY9.2^	NA	GO:00033	NA	PF00400.3	NA	NA
2.06E-05	XP_01150	Q6ZU64.2^	NA	GO:00037	NA	PF15780.4	NA	NA
3.66E-26	AAP36929	P36222.2^	K17523|CH	GO:00302	COG3325|	PF00704.2	GH18	NA
0.017397	XP_01152	Q9P218.4^	NA	GO:00055	KOG3544^	PF00041.2	CBM37	NA
2.30E-10	NP_00008	Q01955.3^	K06237|CC	GO:00052	KOG3544^	PF01391.1	NA	NA

0.008477	NP_00126	Q9Y5Q5.2	K09614|CC	GO:00042	COG5640|	PF00089.2	NA	SignalP-TM
6.63E-09	NP_00038	P43235.1^	K01371|CT	GO:000551	COG4870|	PF00112.2	NA	NA
7.14E-15	NP_00107	O60494.5^	K14616|CU	GO:00055	KOG3714^	PF00431.1	NA	NA
2.11E-05	NP_00102	Q3SXM0.1	NA	NA	KOG2695^	PF00400.3	NA	NA
4.13E-21	NP_00895	Q5RBE4.1^	NA	GO:00057	NA	PF15343.5	NA	NA
2.08E-05	NP_00130	O75912.1^	K00901|dg	GO:00055	KOG0782^	PF00609.1	NA	NA
1.71E-61	XP_016861	Q9P2D7.5^	K10408|DN	GO:00055	COG5245|	PF08393.1	NA	NA
0.004693	XP_016861	Q6ZR08.2^	K10408|DN	GO:00055	COG5245|	PF03028.1	NA	NA
0.015339	NP_00136	Q92874.1^	K11995|DN	GO:00055	NA	PF03372.2	NA	NA
3.81E-16	EAW67225	P14416.2^	K04145|DF	GO:00049	KOG3656^	PF00001.2	NA	SignalP-TM
2.39E-05	XP_016884	Q13474.2^	NA	GO:00082	NA	PF09068.1	NA	NA
1.12E-07	CAP09197.	Q07075.3^	K11141|EN	GO:00041	COG0308|	PF01433.1	NA	SignalP-TM
1.11E-21	XP_01151	Q5MY95.2	K01510|EN	GO:01024	COG5371|	PF01150.1	NA	NA
0.000101	NP_00107	Q17RC7.2^	NA	GO:00001	COG5173|	PF06046.1	NA	NA
0.003698	NP_00012	P12259.4^	K03902|F5	GO:00055	NA	PF00754.2	CBM32	NA
0.041918	XP_00526	Q8NE31.2	NA	NA	NA	PF03962.1	NA	NA
0.028513	EAW87151	Q8IXL6.2^	NA	GO:00055	KOG3829^	NA	NA	SignalP-TM
0.022622	EAW87151	Q8IXL6.2^	NA	GO:00055	KOG3829^	NA	NA	SignalP-TM
0.036614	XP_01151	Q5JUQ0.1^	NA	NA	NA	PF01960.1	NA	NA
7.15E-11	NP_68047	Q96EF6.1^	K10101|FB	GO:00436	NA	PF04300.1	NA	NA
7.43E-07	XP_01687	Q5XX13.2^	K10266|FB	GO:00436	NA	PF00400.3	NA	NA
6.52E-41	NP_00049	P02671.2^	K03903|FG	GO:00052	KOG2579^	PF00147.1	NA	NA
0.011848	NP_00410	Q92914.1^	K04358|FG	GO:00080	KOG3885^	PF00167.1	NA	NA
0.009082	NP_00129	Q92914.1^	K04358|FG	GO:00080	KOG3885^	PF00167.1	NA	NA
2.55E-31	NP_00050	P02679.3^	K03905|FG	GO:00508	KOG2579^	PF00147.1	NA	NA
0.027387	NP_00112	Q96Q35.2	NA	GO:00057	NA	NA	NA	NA
0.021562	XP_01688	Q9BVV2.1	NA	NA	NA	NA	NA	NA
0.002292	NP_95458	Q6VB84.2^	K09397|FC	GO:00009	COG5025|	PF00250.1	NA	NA
2.01E-57	EAW75147	Q9ULV1.2^	K02354|FZ	GO:00015	KOG3577^	PF01534.1	NA	NA
0.014856	NP_99724	Q6ZQY3.4^	K18966|G	GO:00040	COG0076|	PF00282.1	NA	NA
2.79E-15	NP_00127	Q5VVW2.2	NA	GO:00050	KOG3686^	PF02145.1	NA	NA
0.009428	NP_53672	Q9BWX5.1	K17896|G	GO:00037	COG5641|	PF05349.1	NA	NA
9.20E-05	XP_01152	P36269.2^	K18592|G	GO:00363	COG0405|	PF01019.2	NA	SignalP-TM
2.50E-05	AAV38221.	P36382.3^	K07614|GJ	GO:00712	NA	PF00029.1	NA	SignalP-TM
0.015633	NP_00526	P36915.2^	NA	GO:00055	COG1161|	PF01926.2	NA	NA
0.012246	NP_00526	P36915.2^	NA	GO:00055	COG1161|	PF01926.2	NA	NA
5.75E-05	NP_11587	Q9BXV9.2	NA	GO:00004	NA	PF15387.5	NA	NA
5.07E-42	XP_011541	Q3KR37.1^	NA	GO:00154	KOG1032^	PF16016.4	NA	NA
0.009511	XP_00402	Q5QNW6.3	K11252|H	GO:00036	KOG1744^	PF00125.2	NA	NA
0.016062	3AFA_A^C	P68431.2^	K11254|H	GO:00452	COG2036|	PF00125.2	NA	NA
9.41E-05	XP_001862	P02299.4^	K11253|H	GO:00314	COG2036|	PF00125.2	NA	NA
0.010662	3AFA_A^C	P68431.2^	K11254|H	GO:00452	COG2036|	PF00125.2	NA	NA
0.048153	EAW55528	Q6WV72.3	K11254|H	GO:00036	COG2036|	PF15511.5	NA	NA
0.024903	NP_00532	P14317.3^	K06106|CT	GO:00037	NA	PF02218.1	NA	NA
0.000599	NP_00060	P02790.2^	K18977|HF	GO:00152	KOG1565^	PF00045.1	NA	NA

0.000122	XP_01687	P37058.2^	K10207|H	GO:004704	COG0300|	PF00106.2	NA		NA
0.000986	NP_00388	P46695.4^	NA	GO:00096	NA	NA	NA		NA
0.003146	NP_00118	Q0D2I5.2^	NA	GO:00058	NA	PF00038.2	NA		NA
5.04E-14	XP_023397	A6NMD0.1	K06566|IF	GO:00160	NA	PF04505.1	NA		SignalP-TM
0.000248	NP_00113	Q9H0B3.1	NA	GO:00057	NA	PF00612.2	NA		NA
0.030923	EAW52144	P11215.2^	K06461|IT	GO:00015	NA	PF00092.2	NA		NA
0.002207	AAP36313.	Q13572.2^	K00913|IT	GO:00055	NA	PF05770.1	NA		NA
7.83E-06	NP_87238	Q8IYV9.2^	NA	GO:00428	NA	PF16706.4	NA		NA
0.000179	NP_20313	Q9Y2U2.1	K04918|K	GO:00052	COG1226|	PF07885.1	NA		NA
0.014376	XP_01688	Q96KK3.2^	K04931|K	GO:00052	COG1226|	PF00520.3	NA		NA
5.14E-12	NP_06048	Q2KJY2.1^	K10404|KI	GO:00055	COG5059|	PF00225.2	NA		NA
5.10E-13	XP_01688	Q2KJY2.1^	K10404|KI	GO:00055	COG5059|	PF00225.2	NA		NA
0.00984	NP_05735	Q9Y5W3.2	K17845|KL	GO:00036	COG5048|	PF00096.2	NA		NA
0.004508	NP_00100	Q8NEP7.2	NA	GO:00303	KOG0379^	PF13415.5	NA		NA
5.84E-06	EAW66474	A6NCF5.2^	K13957|KL	NA	KOG1072^	PF01344.2	AA5		NA
0.006303	XP_024304	P12035.3^	K07605|K	GO:00051	NA	PF00038.2	NA		NA
0.000118	NP_00226	P19013.4^	K07605|K	GO:00051	NA	PF00038.2	NA		NA
0.047694	NP_03138	A4D0S4.1^	K06245|LA	GO:00071	KOG0994^	PF00053.2	NA		NA
0.006777	NP_00229	P09848.3^	K01229|LC	GO:00084	COG2723|	PF00232.1	GH1		NA
0.019713	AAP36586.	P09382.2^	K06830|LG	GO:00303	KOG3587^	PF00337.2	NA		NA
0.035041	AAP36224.	P56470.1^	K10091|LG	GO:00302	KOG3587^	PF00337.2	NA		NA
0.044896	NP_00130	NA	NA	NA	NA	NA	NA		NA
0.033804	XP_011527	NA	NA	NA	NA	NA	NA		NA
0.017299	XP_011527	NA	NA	NA	NA	NA	NA		NA
0.001551	XP_01151	NA	NA	NA	NA	NA	NA		NA
0.008921	NP_00107	Q9NT99.3	K16360|LR	GO:00519	COG4886|	PF13855.5	NA		NA
0.023142	XP_024307	Q6ZN01.1	NA	GO:00010	NA	NA	NA		NA
0.017873	CAB56045.	P11226.2^	K03991|M	GO:00055	NA	PF00059.2	NA		NA
0.028631	NP_05602	Q9UPX6.3	NA	GO:00015	NA	PF06789.1	NA		NA
0.013137	XP_01685	O75900.2^	K08001|M	GO:00042	KOG1565^	PF00413.2	NA		SignalP-TM
3.14E-05	NP_00242	P22897.1^	K06560|M	GO:00380	NA	PF00059.2	NA		NA
3.29E-11	NP_00101	Q6NTE8.2	NA	GO:00036	NA	PF15749.4	NA		NA
0.031411	NP_00101	Q4VC12.2	K17656|M	GO:00468	NA	PF01753.1	NA		NA
0.000137	NA	NA	NA	NA	NA	PF00131.1	NA		NA
1.94E-18	NA	P02795.1^	NA	GO:00081	NA	PF00131.1	NA		NA
8.93E-06	NP_00103	P15941.3^	K06568|M	GO:00020	NA	PF01390.1	NA		NA
0.010766	AAL89737.	Q685J3.2^	NA	GO:00301	NA	PF01390.1	NA		NA
0.039748	NP_00126	Q8N307.3	NA	GO:00001	NA	NA	NA		NA
3.72E-24	NP_00245	P20592.1^	NA	GO:00055	COG0699|	PF01031.1	NA		NA
0.037826	NP_05579	Q9Y2K3.5^	K10352|M	GO:00510	COG5022|	PF00063.2	NA		NA
5.37E-06	XP_016874	Q8N1T3.2	K10356|M	GO:00037	COG5022|	PF00063.2	NA		NA
4.47E-14	NP_05619	A0A087W	NA	GO:00057	NA	PF06758.1	NA		NA
0.015138	NP_00113	Q96M43.2	NA	GO:00057	NA	PF06758.1	NA		NA
6.76E-06	XP_005271	Q5VWK0.2	NA	GO:00057	NA	PF06758.1	NA		NA
0.001061	XP_01153	Q96M43.2	NA	GO:00057	NA	PF06758.1	NA		NA

2.53E-08	NP_07885	Q8TB73.2^	NA	GO:00055	KOG4806^	PF10179.8	CBM16	NA
1.06E-05	XP_01153	O76050.1^	K01931|N	GO:00468	KOG4172|	PF07177.1	NA	NA
9.55E-07	NP_00127	Q96EH8.2^	K15689|N	GO:00468	KOG4625^	NA	NA	NA
0.000211	XP_011507	Q9BZQ8.1	NA	GO:00019	NA	NA	NA	NA
0.015043	XP_016881	Q86XR2.2^	NA	NA	NA	PF05400.1	NA	NA
0.008813	NP_03138	Q14112.3^	K06826|N	GO:00055	NA	PF07474.1	NA	NA
1.21E-06	XP_006724	Q9NZ94.2^	K07378|N	GO:00508	COG2272|	PF00135.2	CE10	NA
0.001215	NP_06703	Q9NPP4.2	K12805|N	GO:00055	NA	PF05729.1	NA	NA
0.019273	NP_06032	Q9NX02.1	K19409|N	GO:00055	NA	PF05729.1	NA	NA
0.000595	AAB36006.	Q92570.3^	K08559|N	GO:00354	KOG4217^	PF00105.1	NA	NA
3.82E-05	NP_00134	B1AL46.3^	NA	NA	NA	PF12881.6	NA	NA
0.049438	NP_00113	O00408.1^	K18283|P	GO:00041	KOG3689^	PF00233.1	NA	NA
0.005815	NP_00127	Q5SV97.4^	NA	GO:00063	NA	NA	NA	NA
0.030015	NP_73660	P0DP91.1^	NA	GO:00435	NA	PF13843.5	NA	NA
0.036533	NP_69920	Q6UXB8.1	K20412|P	GO:00304	COG2340|	PF00188.2	NA	NA
0.048607	NP_03846	Q9UKJ1.3^	K15411|P	GO:00422	NA	PF07686.1	NA	NA
0.010041	NP_44312	Q7Z442.5^	K04988|P	GO:00052	KOG3599^	PF08016.1	NA	NA
1.49E-08	XP_016882	Q9UP65.2	K16342|P	GO:00055	KOG1325^	PF01735.1	NA	NA
0.000111	NP_00119	Q3MJ16.4	K16342|P	GO:00055	KOG1028|	PF01735.1	NA	NA
0.017233	NP_00115	Q9ULL4.2^	K06821|P	GO:00986	KOG3610^	PF08337.1	NA	SignalP-TM
0.012818	XP_016882	P09086.3^	K09364|P	GO:00012	KOG3802^	PF00157.1	NA	NA
4.11E-14	NP_00133	P50336.1^	K00231|P	GO:00506	COG1232|	PF01593.2	NA	NA
0.020145	NP_00130	P21246.1^	K16642|P	GO:00353	NA	PF01091.1	NA	NA
2.48E-10	NP_00133	Q86Y79.1^	K01056|P	GO:00040	COG0193|	PF01195.1	NA	NA
4.74E-19	NP_00560	P11217.6^	K00688|E	GO:00081	COG0058|	PF00343.1	GT35	NA
0.000198	NP_06052	Q96JH8.5^	NA	GO:00072	NA	PF01843.1	NA	NA
0.039274	NP_00095	P10826.2^	K08528|N	GO:00036	KOG3575^	PF00104.2	NA	NA
0.016035	BAD92569.	O75452.2^	K11154|R	GO:00470	KOG1610^	PF00106.2	NA	NA
0.002791	XP_011508	Q52LD8.3^	NA	GO:00332	NA	PF15250.5	NA	NA
0.038381	NP_73346	O76081.4^	K16449|R	GO:00050	KOG3589^	PF00615.1	NA	NA
0.001244	NP_00118	M0QZC1.2	NA	GO:00468	NA	PF13639.5	NA	NA
0.018484	XP_011535	Q96KN7.2	K16512|R	GO:00424	NA	PF11618.7	NA	NA
0.001771	XP_011513	P0C881.1^	NA	NA	COG4642|	PF02493.1	NA	NA
0.006466	XP_006715	B2RC85.2^	NA	NA	COG4642|	PF02493.1	NA	NA
0.015342	XP_01688	Q14162.3^	NA	GO:00301	KOG1218^	NA	NA	SignalP-TM
0.000767	NP_00368	Q14162.3^	NA	GO:00301	KOG1218^	PF00053.2	NA	NA
0.016039	NP_00102	Q4G0G5.1	NA	GO:00055	COG5048|	PF01099.1	NA	NA
0.001362	NP_00127	Q8N228.2	K11466|S	GO:00056	KOG3766^	PF12140.7	NA	NA
0.005486	NP_00112	P51172.3^	K04826|S	GO:00152	KOG4294^	PF00858.2	NA	NA
0.043164	NP_07176	Q9H3S1.2^	K06521|S	GO:00454	KOG3611^	PF01403.1	NA	NA
5.05E-35	NP_00113	O00141.2^	K13302|S	GO:00055	KOG0598^	PF00069.2	NA	NA
0.002777	NP_00109	Q8TEJ3.2^	K12171|S	GO:00468	KOG2177^	PF14604.5	NA	NA
2.83E-06	NP_00103	P14410.6^	K01203|S	GO:00302	COG1501|	PF01055.2	GH31	SignalP-TM
0.003534	NP_00131	O00241.5^	K06551|S	GO:00071	NA	PF07686.1	NA	NA
0.04818	XP_01688	Q86WV1.3	K17699|S	GO:00510	NA	PF00169.2	NA	NA

0.008943	NP_00107	A6NNN8.1	K14994|SL	GO:001517	COG0814|	PF01490.1	NA	SignalP-TM
0.001578	NP_05726	Q9UMX9.2	K15378|SL	GO:00085	KOG0637^	PF07690.1	NA	NA
0.000282	NP_44317	Q8WWX8.	K14391|SL	GO:000541	COG4146^	PF00474.1	NA	SignalP-TM
0.018165	NP_00133	Q8WWX8.	K14391|SL	GO:000541	COG4146^	PF00474.1	NA	SignalP-TM
5.70E-06	NP_05587	Q9UPR5.2	K05849|SL	GO:000543	KOG1306^	PF03160.1	GT66	NA
0.000944	XP_005254	Q96L94.1^	K17941|SN	GO:003509	NA	PF00787.2	NA	NA
0.02387	XP_005267	A7XYQ1.2^	NA	GO:004687	NA	PF15279.5	NA	NA
9.02E-07	XP_016784	O15524.1^	K04694|SC	GO:004693	KOG4566^	PF00017.2	NA	NA
3.21E-33	NP_87435	Q8IXZ3.3^	K09198|SP	GO:000367	COG5048|	PF13465.5	NA	NA
2.00E-07	NP_54301	Q9H3Y6.1^	K08895|SR	GO:000552	COG0515|	PF07714.1	NA	NA
0.012648	NP_00116	B8Y466.1^	K08832|SR	GO:000552	KOG1290^	PF00069.2	NA	NA
0.000112	AAX36847.	P0DMM9.1	K01014|SL	GO:004768	KOG1584^	PF00685.2	NA	NA
0.008293	EAW64451	O60279.3^	NA	GO:000554	NA	PF00084.1	NA	NA
0.003694	XP_024304	Q8IV01.1^	K19912|SY	GO:00055	NA	PF00168.2	NA	NA
0.003658	NP_54277	Q96C24.2^	K17598|SY	GO:004687	KOG1028^	PF00168.2	NA	NA
0.002955	NP_00366	O15273.1^	K19879|TC	GO:003612	NA	PF09470.9	NA	NA
0.000346	XP_01688	Q8IWB6.2	K17540|TE	GO:000552	NA	PF07714.1	NA	NA
6.48E-12	NP_00118	Q9CQT9.1	NA	GO:000573	KOG0773|	PF07019.1	NA	NA
0.033158	NP_07903	Q9H808.2^	NA	GO:007049	KOG0639^	PF00400.3	NA	NA
0.005615	XP_011517	Q5W0B7.1	NA	GO:001602	NA	PF09772.8	NA	SignalP-TM
0.026484	NP_00123	Q2WGJ8.1	NA	GO:001602	NA	PF15158.5	NA	NA
3.47E-09	NP_11625	Q8IZW8.3^	K18080|TN	GO:000377	NA	PF08416.1	NA	NA
0.044073	BAD92249.	P22105.5^	K06252|TN	GO:009863	KOG1225|	PF00147.1	NA	NA
3.30E-17	NP_06019	Q9NXH8.2	NA	GO:000552	KOG2170^	PF06309.1	NA	NA
0.013099	NP_00417	P17752.4^	K00502|TP	GO:00055	COG3186|	PF00351.2	NA	NA
1.42E-30	NP_00117	Q9BX84.2^	K04981|TR	GO:000552	NA	PF02816.1	NA	NA
0.000417	XP_01686	Q5TCY1.2^	K08815|TT	GO:000552	KOG1164^	PF00069.2	NA	NA
0.002194	NP_99719	Q6PEY2.2^	K07374|TL	GO:000552	COG5023|	PF00091.2	NA	NA
3.34E-05	NP_11040	Q9H4B7.1	K07375|TL	GO:000552	COG5023|	PF00091.2	NA	NA
0.000861	NP_00045	P22309.1^	K00699|U	GO:001989	COG1819|	PF00201.1	GT1	SignalP-TM
0.044	NP_06182	Q9UKP6.1	K04241|U	GO:000852	KOG3656^	PF00001.2	NA	SignalP-TM
0.048529	NP_00127	Q8N1B4.1	K20298|VF	GO:001713	KOG1961^	PF04129.1	NA	NA
0.001369	XP_00671	Q86TI4.3^	NA	NA	NA	PF00400.3	NA	NA
0.001517	XP_003281	P56706.2^	K00572|W	GO:00051	KOG3913^	PF00110.1	NA	SignalP-TM
0.034868	EAX01919.	P43403.1^	K07360|ZA	GO:000552	COG0515^	PF07714.1	NA	NA
0.001247	NP_11621	Q96SR6.3^	K09228|KF	GO:000367	COG5048|	PF13465.5	NA	NA
0.002984	NP_00133	Q8NDP4.1	K09228|KF	GO:000367	COG5048|	PF13465.5	NA	NA
1.96E-12	BAC87108.	Q02386.2^	K09228|KF	GO:000367	COG5048|	PF13465.5	NA	NA
0.036569	NP_99721	Q7Z7K2.1^	NA	GO:000367	COG5048|	PF13465.5	NA	NA
2.14E-06	NP_00101	Q0D2J5.2^	K09228|KF	GO:000367	COG5048|	PF13465.5	NA	NA
0.003859	BAG62859.	P21918.2^	NA	GO:003524	KOG3656^	PF00001.2	NA	NA

tmhmm
i25-47o639-661i681-703o718-740i745-767o1346-1368i1655-1677o1702-1724i1737-1759o1769-1791i[illegible]
NA
o49-71i84-106o121-140i161-183o216-238i251-273o293-315i
NA
NA
NA
NA
NA
o141-163i172-194o237-259i272-294o333-355i375-397o402-424i
NA
NA
NA
NA
NA
NA
NA
NA
i189-211o266-285i331-353o373-392i
i5-27o
i7-29o
o15-34i47-66o76-98i193-215o
NA
NA
NA
i12-34o
i34-56o225-242i401-423o438-457i901-923o
NA
NA
NA
NA
NA
NA
i184-206o
NA
NA
o5-24i195-217o1058-1080i
NA
NA
i83-105o
NA
i187-209o
NA
NA
NA

i46-68o
NA
NA
NA
NA
NA
NA
NA
NA
o37-59i72-94o109-131i152-174o194-216i375-397o407-429i
NA
i21-40o
i117-139o543-565i
NA
NA
NA
i9-31o
i9-31o
NA
NA
NA
NA
NA
NA
i9-31o
NA
NA
NA
o10-32i221-243o253-275i301-323o394-416i437-459o474-496i
NA
NA
NA
i7-29o
i20-37o77-99i165-184o207-229i
NA
NA
NA
o666-688i
NA
NA
NA
NA
NA
NA
NA

NA
NA
NA
i28-50o
NA
NA
NA
o117-139i
i7-29o92-114i121-140o168-190i197-216o231-253i
o306-328i379-398o437-459i
NA
NA
NA
NA
NA
NA
NA
NA
o1881-1903i
NA
NA
NA
NA
NA
NA
o575-597i
NA
NA
o892-914i
i20-42o
o1388-1410i
NA
NA
NA
NA
NA
o4392-4414i
NA
NA
NA
NA
NA
NA
NA
NA

NA
NA
NA
NA
NA
NA
o591-613i
NA
NA
NA
NA
NA
NA
NA
i7-29o
o196-218i
o661-680i867-889o904-926i1137-1159o1179-1201i1260-1282o1495-1517i1530-1552o1584-1606i162[illegible]
NA
NA
NA
NA
NA
i13-30o
NA
NA
NA
NA
i144-166o
NA
NA
o201-223i
NA
NA
NA
NA
NA
NA
NA
NA
o681-703i
NA
NA
i13-32o
o154-176i
NA

i21-43o58-80i176-198o218-240i253-275o295-317i343-365o375-397i410-432o
i43-65o70-92i105-127o137-159i179-200o215-237i320-342o365-387i394-416o421-443i476-498o503-5
o15-37i42-64o74-96i109-131o141-163i243-265o311-333i354-376o386-408i415-437o457-479i591-610
o29-46i66-88o103-125i138-160o180-202i326-348o358-380i387-409o429-451i563-582o
i73-95o162-184i197-219o223-245i745-767o782-804i824-843o853-875i896-918o
NA
NA
NA
NA
NA
NA
NA
o466-488i
NA
NA
NA
NA
o70-89i96-118o133-152i
NA
i173-192o212-244i
NA
NA
NA
NA
NA
o738-755i834-856o900-922i935-954o969-988i1047-1069o
NA
NA
NA
i13-35o491-513i
o53-75i88-110o125-147i168-190o210-232i259-281o296-318i
NA
NA
NA
NA
NA
NA
NA
NA
NA
o156-178i190-212o227-246i

1848-1870o

7-1649o1687-1709i

25i
o

Supplementary Material 4

Term	Database	ID	Input_num	Backgroun	P_Value	Corrected_	Input	Hyperlink
Taurine an	KEGG PATH	ko00430	3	12	0.000345	0.037617	K00659:ge	http://wwv
Compleme	KEGG PATH	ko04610	6	99	0.000476	0.037617	K03990:ge	http://wwv
Alcoholism	KEGG PATH	ko05034	7	188	0.002521	0.132782	K11254:ge	http://wwv
Rap1 signa	KEGG PATH	ko04015	7	217	0.005392	0.172507	K06461:ge	http://wwv
Galactose r	KEGG PATH	ko00052	3	35	0.005459	0.172507	K00011:ge	http://wwv
Systemic lu	KEGG PATH	ko05322	7	235	0.008119	0.208134	K05699:ge	http://wwv
Ras signalir	KEGG PATH	ko04014	7	241	0.009221	0.208134	K16342:ge	http://wwv
Amoebiasis	KEGG PATH	ko05146	4	109	0.022229	0.375313	K05699:ge	http://wwv
Pathogenic	KEGG PATH	ko05130	3	62	0.023518	0.375313	K07374:ge	http://wwv
Starch and	KEGG PATH	ko00500	3	63	0.024472	0.375313	K00688:ge	http://wwv
Arachidoni	KEGG PATH	ko00590	3	67	0.028494	0.375313	K16342:ge	http://wwv
alpha-Linol	KEGG PATH	ko00592	2	26	0.028505	0.375313	K16342:ge	http://wwv
Linoleic aci	KEGG PATH	ko00591	2	30	0.036502	0.394675	K16342:ge	http://wwv
beta-Alanir	KEGG PATH	ko00410	2	31	0.038621	0.394675	K00276:ge	http://wwv
Legionellos	KEGG PATH	ko05134	3	76	0.038741	0.394675	K03990:ge	http://wwv
Platelet act	KEGG PATH	ko04611	4	132	0.039967	0.394675	K16342:ge	http://wwv
Viral carcin	KEGG PATH	ko05203	6	279	0.053365	0.398269	K05699:ge	http://wwv
Tropane, p	KEGG PATH	ko00960	1	5	0.055342	0.398269	K00276:ge	http://wwv
Pentose an	KEGG PATH	ko00040	2	40	0.059635	0.398269	K00011:ge	http://wwv
ECM-recep	KEGG PATH	ko04512	3	92	0.060921	0.398269	K06252:ge	http://wwv
Small cell lu	KEGG PATH	ko05222	3	92	0.060921	0.398269	K06245:ge	http://wwv
PI3K-Akt si	KEGG PATH	ko04151	7	363	0.061466	0.398269	K04358:ge	http://wwv
Pertussis	KEGG PATH	ko05133	3	93	0.062469	0.398269	K03990:ge	http://wwv
GnRH signa	KEGG PATH	ko04912	3	95	0.065618	0.398269	K16342:ge	http://wwv
Staphyloco	KEGG PATH	ko05150	4	159	0.068683	0.398269	K03990:ge	http://wwv
Cyanoamin	KEGG PATH	ko00460	1	7	0.073105	0.398269	K18592:ge	http://wwv
Porphyrin a	KEGG PATH	ko00860	2	46	0.075354	0.398269	K00231:ge	http://wwv
Inflammato	KEGG PATH	ko04750	3	102	0.077199	0.398269	K16342:ge	http://wwv
Ether lipid	KEGG PATH	ko00565	2	47	0.07809	0.398269	K16342:ge	http://wwv
Gap junctio	KEGG PATH	ko04540	3	103	0.078922	0.398269	K04145:ge	http://wwv
Phagosome	KEGG PATH	ko04145	6	310	0.079164	0.398269	K03990:ge	http://wwv
Phosphatic	KEGG PATH	ko04070	3	104	0.080662	0.398269	K02183:ge	http://wwv
Melanoger	KEGG PATH	ko04916	3	106	0.084192	0.403102	K00572:ge	http://wwv
Carbohydra	KEGG PATH	ko04973	2	51	0.089335	0.415147	K01203:ge	http://wwv
Choline me	KEGG PATH	ko05231	3	111	0.0933	0.416237	K16342:ge	http://wwv
Ovarian ste	KEGG PATH	ko04913	2	53	0.095127	0.416237	K16342:ge	http://wwv
Pathways i	KEGG PATH	ko05200	7	407	0.097473	0.416237	K04358:ge	http://wwv
Glyceropho	KEGG PATH	ko00564	3	115	0.100867	0.419396	K16342:ge	http://wwv
Isoquinolin	KEGG PATH	ko00950	1	11	0.107642	0.434709	K00276:ge	http://wwv
Basal cell c	KEGG PATH	ko05217	2	58	0.110053	0.434709	K02354:ge	http://wwv
Serotonerg	KEGG PATH	ko04726	3	123	0.116704	0.449738	K00502:ge	http://wwv
Plant-path	KEGG PATH	ko04626	1	13	0.124428	0.460916	K02183:ge	http://wwv
Long-term	KEGG PATH	ko04730	2	64	0.128702	0.460916	K16342:ge	http://wwv
VEGF signa	KEGG PATH	ko04370	2	64	0.128702	0.460916	K16342:ge	http://wwv

Vascular sr	KEGG PATH	ko04270	3	130	0.131274	0.460916	K16342:ge	http://wwv
Glycerolipi	KEGG PATH	ko00561	2	69	0.144761	0.496327	K00011:ge	http://wwv
Retinol me	KEGG PATH	ko00830	2	70	0.148022	0.496327	K11154:ge	http://wwv
Focal adhe	KEGG PATH	ko04510	4	215	0.153181	0.496327	K05699:ge	http://wwv
Steroid hor	KEGG PATH	ko00140	2	72	0.154589	0.496327	K00699:ge	http://wwv
Primary bil	KEGG PATH	ko00120	1	17	0.157066	0.496327	K00659:ge	http://wwv
Regulation	KEGG PATH	ko04810	4	220	0.162106	0.501451	K05699:ge	http://wwv
Phenylalan	KEGG PATH	ko00360	1	18	0.165034	0.501451	K00276:ge	http://wwv
Melanoma	KEGG PATH	ko05218	2	77	0.171238	0.505976	K04358:ge	http://wwv
Pantothen	KEGG PATH	ko00770	1	19	0.172929	0.505976	K18966:ge	http://wwv
Fc epsilon I	KEGG PATH	ko04664	2	79	0.17798	0.511287	K16342:ge	http://wwv
Phospholip	KEGG PATH	ko04072	3	153	0.183066	0.516509	K16342:ge	http://wwv
Tight juncti	KEGG PATH	ko04530	3	156	0.190191	0.516578	K05699:ge	http://wwv
Insulin sign	KEGG PATH	ko04910	3	157	0.192582	0.516578	K00688:ge	http://wwv
Hippo signa	KEGG PATH	ko04390	3	158	0.19498	0.516578	K02354:ge	http://wwv
Biosynthes	KEGG PATH	ko01040	1	22	0.196169	0.516578	K00659:ge	http://wwv
Renin-angi	KEGG PATH	ko04614	1	23	0.203771	0.517257	K11141:ge	http://wwv
Vitamin dig	KEGG PATH	ko04977	1	23	0.203771	0.517257	K14616:ge	http://wwv
Chemical c	KEGG PATH	ko05204	2	88	0.208769	0.517257	K00699:ge	http://wwv
Oxytocin si	KEGG PATH	ko04921	3	164	0.209522	0.517257	K16342:ge	http://wwv
Transcripti	KEGG PATH	ko05202	3	168	0.219348	0.533185	K06461:ge	http://wwv
Aldosteron	KEGG PATH	ko04925	2	94	0.22959	0.541839	K02183:ge	http://wwv
cGMP-PKG	KEGG PATH	ko04022	3	174	0.234261	0.541839	K05849:ge	http://wwv
mTOR signa	KEGG PATH	ko04150	3	176	0.239273	0.541839	K02354:ge	http://wwv
Tuberculos	KEGG PATH	ko05152	4	260	0.239353	0.541839	K03990:ge	http://wwv
Protein dig	KEGG PATH	ko04974	2	97	0.240055	0.541839	K05849:ge	http://wwv
Phototrans	KEGG PATH	ko04745	1	29	0.247905	0.551676	K02183:ge	http://wwv
Ascorbate	KEGG PATH	ko00053	1	30	0.255021	0.559555	K00699:ge	http://wwv
Phototrans	KEGG PATH	ko04744	1	31	0.26207	0.559555	K02183:ge	http://wwv
Circadian r	KEGG PATH	ko04710	1	31	0.26207	0.559555	K03730:ge	http://wwv
Glucagon s	KEGG PATH	ko04922	2	109	0.282059	0.594203	K00688:ge	http://wwv
Fructose ar	KEGG PATH	ko00051	1	35	0.28961	0.595504	K00011:ge	http://wwv
Osteoclast	KEGG PATH	ko04380	3	196	0.290214	0.595504	K04694:ge	http://wwv
Tyrosine m	KEGG PATH	ko00350	1	37	0.302995	0.613759	K00276:ge	http://wwv
MAPK signa	KEGG PATH	ko04010	4	293	0.308346	0.616692	K16342:ge	http://wwv
Glutamater	KEGG PATH	ko04724	2	119	0.316945	0.620436	K16342:ge	http://wwv
Aldosteron	KEGG PATH	ko04960	1	40	0.322604	0.620436	K13302:ge	http://wwv
cAMP signa	KEGG PATH	ko04024	3	210	0.326365	0.620436	K04145:ge	http://wwv
Glycine, se	KEGG PATH	ko00260	1	41	0.329018	0.620436	K00276:ge	http://wwv
Proteoglyc	KEGG PATH	ko05205	3	213	0.334124	0.620436	K02354:ge	http://wwv
Tryptophar	KEGG PATH	ko00380	1	42	0.335371	0.620436	K00502:ge	http://wwv
Leukocyte	KEGG PATH	ko04670	2	125	0.337706	0.620436	K05699:ge	http://wwv
Mineral ab	KEGG PATH	ko04978	1	43	0.341665	0.620495	K04981:ge	http://wwv
Fat digestic	KEGG PATH	ko04975	1	46	0.360194	0.646711	K05641:ge	http://wwv
Dopaminer	KEGG PATH	ko04728	2	140	0.388689	0.684292	K04145:ge	http://wwv

Drug metal	KEGG PATH	ko00983	1	51	0.389931	0.684292	K00699:gei	http://wwv
FoxO signa	KEGG PATH	ko04068	2	143	0.398689	0.684292	K17845:gei	http://wwv
Primary im	KEGG PATH	ko05340	1	53	0.401438	0.684292	K07360:gei	http://wwv
Glutathion	KEGG PATH	ko00480	1	54	0.407111	0.684292	K18592:gei	http://wwv
Cocaine ad	KEGG PATH	ko05030	1	54	0.407111	0.684292	K04145:gei	http://wwv
Sphingolipi	KEGG PATH	ko00600	1	58	0.429271	0.689857	K12349:gei	http://wwv
Signaling p	KEGG PATH	ko04550	2	153	0.431457	0.689857	K02354:gei	http://wwv
Type II diak	KEGG PATH	ko04930	1	59	0.434682	0.689857	K04694:gei	http://wwv
Wnt signali	KEGG PATH	ko04310	2	154	0.434683	0.689857	K02354:gei	http://wwv
MicroRNAs	KEGG PATH	ko05206	2	157	0.444302	0.689857	K04694:gei	http://wwv
Apoptosis	KEGG PATH	ko04210	2	157	0.444302	0.689857	K07374:gei	http://wwv
Leishmania	KEGG PATH	ko05140	2	157	0.444302	0.689857	K03990:gei	http://wwv
ABC transp	KEGG PATH	ko02010	1	61	0.445351	0.689857	K05641:gei	http://wwv
Cell adhesi	KEGG PATH	ko04514	3	266	0.468288	0.703132	K07378:gei	http://wwv
Non-small	KEGG PATH	ko05223	1	66	0.471156	0.703132	K08528:gei	http://wwv
Renin secre	KEGG PATH	ko04924	1	67	0.476172	0.703132	K02183:gei	http://wwv
Shigellosis	KEGG PATH	ko05131	1	67	0.476172	0.703132	K06106:gei	http://wwv
NOD-like re	KEGG PATH	ko04621	1	67	0.476172	0.703132	K12805:gei	http://wwv
Glioma	KEGG PATH	ko05214	1	70	0.490938	0.712137	K02183:gei	http://wwv
Long-term	KEGG PATH	ko04720	1	71	0.495768	0.712137	K02183:gei	http://wwv
Amphetam	KEGG PATH	ko05031	1	72	0.500553	0.712137	K02183:gei	http://wwv
Bile secreti	KEGG PATH	ko04976	1	74	0.509986	0.712137	K00659:gei	http://wwv
Arrhythmo	KEGG PATH	ko05412	1	74	0.509986	0.712137	K05699:gei	http://wwv
Axon guida	KEGG PATH	ko04360	2	180	0.514881	0.712137	K06821:gei	http://wwv
Inositol ph	KEGG PATH	ko00562	1	76	0.519243	0.712137	K00913:gei	http://wwv
Gastric acid	KEGG PATH	ko04971	1	77	0.523806	0.712137	K02183:gei	http://wwv
Prolactin si	KEGG PATH	ko04917	1	77	0.523806	0.712137	K04694:gei	http://wwv
Adherens j	KEGG PATH	ko04520	1	80	0.537239	0.712137	K05699:gei	http://wwv
Drug metal	KEGG PATH	ko00982	1	80	0.537239	0.712137	K00699:gei	http://wwv
Calcium sig	KEGG PATH	ko04020	2	188	0.538024	0.712137	K05849:gei	http://wwv
Purine met	KEGG PATH	ko00230	2	189	0.540863	0.712137	K01510:gei	http://wwv
Bacterial in	KEGG PATH	ko05100	1	82	0.545985	0.712939	K06106:gei	http://wwv
Metabolism	KEGG PATH	ko00980	1	84	0.554566	0.718209	K00699:gei	http://wwv
Peroxisome	KEGG PATH	ko04146	1	88	0.571249	0.7338	K00659:gei	http://wwv
TGF-beta si	KEGG PATH	ko04350	1	90	0.579356	0.737358	K04665:gei	http://wwv
Hematopoi	KEGG PATH	ko04640	1	91	0.583353	0.737358	K06461:gei	http://wwv
Huntingtor	KEGG PATH	ko05016	2	208	0.592515	0.739326	K10408:gei	http://wwv
Salivary sec	KEGG PATH	ko04970	1	94	0.595117	0.739326	K02183:gei	http://wwv
Salmonella	KEGG PATH	ko05132	1	95	0.598965	0.739326	K12805:gei	http://wwv
Neuroactiv	KEGG PATH	ko04080	3	330	0.612259	0.739326	K04145:gei	http://wwv
Toxoplasm	KEGG PATH	ko05145	2	216	0.612942	0.739326	K04694:gei	http://wwv
Circadian e	KEGG PATH	ko04713	1	99	0.613995	0.739326	K02183:gei	http://wwv
Morphine a	KEGG PATH	ko05032	1	100	0.617665	0.739326	K18283:gei	http://wwv
HIF-1 signa	KEGG PATH	ko04066	1	102	0.6249	0.742363	K05466:gei	http://wwv
Pyrimidine	KEGG PATH	ko00240	1	113	0.662332	0.780958	K01510:gei	http://wwv

AGE-RAGE	KEGG PATH	ko04933	1	117	0.675003	0.786429	K06237:gen	http://www
T cell recep	KEGG PATH	ko04660	1	118	0.678096	0.786429	K07360:gen	http://www
Chagas dise	KEGG PATH	ko05142	1	120	0.684195	0.786429	K03990:gen	http://www
NF-kappa E	KEGG PATH	ko04064	1	121	0.687202	0.786429	K07360:gen	http://www
Toll-like rec	KEGG PATH	ko04620	1	125	0.698946	0.786429	K01371:gen	http://www
Estrogen si	KEGG PATH	ko04915	1	125	0.698946	0.786429	K02183:gen	http://www
Insulin resi	KEGG PATH	ko04931	1	126	0.701813	0.786429	K00688:gen	http://www
Neurotropl	KEGG PATH	ko04722	1	128	0.707466	0.78718	K02183:gen	http://www
Sphingolipi	KEGG PATH	ko04071	1	135	0.726426	0.794516	K12349:gen	http://www
Oocyte me	KEGG PATH	ko04114	1	137	0.731615	0.794516	K02183:gen	http://www
Ubiquitin n	KEGG PATH	ko04120	1	137	0.731615	0.794516	K04694:gen	http://www
Lysosome	KEGG PATH	ko04142	1	138	0.734173	0.794516	K01371:gen	http://www
Parkinson's	KEGG PATH	ko05012	1	148	0.758458	0.815214	K04145:gen	http://www
Adrenergic	KEGG PATH	ko04261	1	160	0.784709	0.83773	K02183:gen	http://www
Jak-STAT si	KEGG PATH	ko04630	1	169	0.802519	0.850993	K04694:gen	http://www
Alzheimer's	KEGG PATH	ko05010	1	188	0.835457	0.871518	K02183:gen	http://www
Rheumatoi	KEGG PATH	ko05323	1	189	0.83703	0.871518	K01371:gen	http://www
Herpes sim	KEGG PATH	ko05168	2	341	0.838423	0.871518	K03990:gen	http://www
HTLV-I infe	KEGG PATH	ko05166	2	393	0.890895	0.920009	K02354:gen	http://www
Olfactory tı	KEGG PATH	ko04740	3	567	0.909451	0.931385	K05849:gen	http://www
Epstein-Ba	KEGG PATH	ko05169	1	255	0.9137	0.931385	K01510:gen	http://www
Cytokine-c	KEGG PATH	ko04060	1	308	0.948312	0.960065	K04665:gen	http://www
Endocytosi	KEGG PATH	ko04144	1	320	0.953988	0.960065	K12489:gen	http://www
Natural kill	KEGG PATH	ko04650	1	521	0.993538	0.993538	K07360:gen	http://www

v.genome.jp/kegg-bin/show_pathway?ko00430/K00659%09green/K18592%09red/K18966%09green
v.genome.jp/kegg-bin/show_pathway?ko04610/K03990%09green/K03991%09green/K03905%09green
v.genome.jp/kegg-bin/show_pathway?ko05034/K11254%09yellow/K02183%09red/K04145%09green/I
v.genome.jp/kegg-bin/show_pathway?ko04015/K06461%09green/K04358%09red/K17699%09red/K04
v.genome.jp/kegg-bin/show_pathway?ko00052/K00011%09green/K01229%09green/K01203%09green
v.genome.jp/kegg-bin/show_pathway?ko05322/K05699%09green/K11254%09yellow/K03990%09greer
v.genome.jp/kegg-bin/show_pathway?ko04014/K16342%09green/K02183%09red/K04358%09red/K05
v.genome.jp/kegg-bin/show_pathway?ko05146/K05699%09green/K06245%09green/K06461%09green
v.genome.jp/kegg-bin/show_pathway?ko05130/K07374%09green/K07375%09green/K06106%09green
v.genome.jp/kegg-bin/show_pathway?ko00500/K00688%09green/K00699%09red/K01203%09green
v.genome.jp/kegg-bin/show_pathway?ko00590/K16342%09green/K18592%09red
v.genome.jp/kegg-bin/show_pathway?ko00592/K16342%09green
v.genome.jp/kegg-bin/show_pathway?ko00591/K16342%09green
v.genome.jp/kegg-bin/show_pathway?ko00410/K00276%09green/K18966%09green
v.genome.jp/kegg-bin/show_pathway?ko05134/K03990%09green/K06461%09green/K12805%09green
v.genome.jp/kegg-bin/show_pathway?ko04611/K16342%09green/K03905%09green/K03903%09green
v.genome.jp/kegg-bin/show_pathway?ko05203/K05699%09green/K11254%09yellow/K03990%09greer
v.genome.jp/kegg-bin/show_pathway?ko00960/K00276%09green
v.genome.jp/kegg-bin/show_pathway?ko00040/K00011%09green/K00699%09red
v.genome.jp/kegg-bin/show_pathway?ko04512/K06252%09green/K06245%09green/K06237%09green
v.genome.jp/kegg-bin/show_pathway?ko05222/K06245%09green/K08528%09green/K06237%09green
v.genome.jp/kegg-bin/show_pathway?ko04151/K04358%09red/K13302%09green/K06252%09green/K
v.genome.jp/kegg-bin/show_pathway?ko05133/K03990%09green/K06461%09green/K02183%09red
v.genome.jp/kegg-bin/show_pathway?ko04912/K16342%09green/K02183%09red
v.genome.jp/kegg-bin/show_pathway?ko05150/K03990%09green/K03991%09green/K03905%09green
v.genome.jp/kegg-bin/show_pathway?ko00460/K18592%09red
v.genome.jp/kegg-bin/show_pathway?ko00860/K00231%09red/K00699%09red
v.genome.jp/kegg-bin/show_pathway?ko04750/K16342%09green/K02183%09red
v.genome.jp/kegg-bin/show_pathway?ko00565/K16342%09green
v.genome.jp/kegg-bin/show_pathway?ko04540/K04145%09green/K07374%09green/K07375%09green
v.genome.jp/kegg-bin/show_pathway?ko04145/K03990%09green/K03991%09green/K06461%09green
v.genome.jp/kegg-bin/show_pathway?ko04070/K02183%09red/K00913%09red/K00901%09green
v.genome.jp/kegg-bin/show_pathway?ko04916/K00572%09red/K02354%09green/K02183%09red
v.genome.jp/kegg-bin/show_pathway?ko04973/K01203%09green/K01229%09green
v.genome.jp/kegg-bin/show_pathway?ko05231/K16342%09green/K00901%09green
v.genome.jp/kegg-bin/show_pathway?ko04913/K16342%09green
v.genome.jp/kegg-bin/show_pathway?ko05200/K04358%09red/K02354%09green/K08528%09green/K
v.genome.jp/kegg-bin/show_pathway?ko00564/K16342%09green/K00901%09green
v.genome.jp/kegg-bin/show_pathway?ko00950/K00276%09green
v.genome.jp/kegg-bin/show_pathway?ko05217/K02354%09green/K00572%09red
v.genome.jp/kegg-bin/show_pathway?ko04726/K00502%09green/K16342%09green
v.genome.jp/kegg-bin/show_pathway?ko04626/K02183%09red
v.genome.jp/kegg-bin/show_pathway?ko04730/K16342%09green
v.genome.jp/kegg-bin/show_pathway?ko04370/K16342%09green

v.genome.jp/kegg-bin/show_pathway?ko04270/K16342%09green/K02183%09red
v.genome.jp/kegg-bin/show_pathway?ko00561/K00011%09green/K00901%09green
v.genome.jp/kegg-bin/show_pathway?ko00830/K11154%09green/K00699%09red
v.genome.jp/kegg-bin/show_pathway?ko04510/K05699%09green/K06252%09green/K06245%09green
v.genome.jp/kegg-bin/show_pathway?ko00140/K00699%09red/K10207%09green
v.genome.jp/kegg-bin/show_pathway?ko00120/K00659%09green
v.genome.jp/kegg-bin/show_pathway?ko04810/K05699%09green/K06461%09green/K04358%09red
v.genome.jp/kegg-bin/show_pathway?ko00360/K00276%09green
v.genome.jp/kegg-bin/show_pathway?ko05218/K04358%09red
v.genome.jp/kegg-bin/show_pathway?ko00770/K18966%09green
v.genome.jp/kegg-bin/show_pathway?ko04664/K16342%09green
v.genome.jp/kegg-bin/show_pathway?ko04072/K16342%09green/K00901%09green
v.genome.jp/kegg-bin/show_pathway?ko04530/K05699%09green/K10352%09green/K06106%09green
v.genome.jp/kegg-bin/show_pathway?ko04910/K00688%09green/K04694%09green/K02183%09red
v.genome.jp/kegg-bin/show_pathway?ko04390/K02354%09green/K04665%09green/K00572%09red
v.genome.jp/kegg-bin/show_pathway?ko01040/K00659%09green
v.genome.jp/kegg-bin/show_pathway?ko04614/K11141%09red
v.genome.jp/kegg-bin/show_pathway?ko04977/K14616%09green
v.genome.jp/kegg-bin/show_pathway?ko05204/K00699%09red/K01014%09red
v.genome.jp/kegg-bin/show_pathway?ko04921/K16342%09green/K02183%09red
v.genome.jp/kegg-bin/show_pathway?ko05202/K06461%09green/K08559%09green/K11253%09green
v.genome.jp/kegg-bin/show_pathway?ko04925/K02183%09red/K18283%09green
v.genome.jp/kegg-bin/show_pathway?ko04022/K05849%09green/K02183%09red/K18283%09green
v.genome.jp/kegg-bin/show_pathway?ko04150/K02354%09green/K13302%09green/K00572%09red
v.genome.jp/kegg-bin/show_pathway?ko05152/K03990%09green/K06461%09green/K06560%09green
v.genome.jp/kegg-bin/show_pathway?ko04974/K05849%09green/K06237%09green
v.genome.jp/kegg-bin/show_pathway?ko04745/K02183%09red
v.genome.jp/kegg-bin/show_pathway?ko00053/K00699%09red
v.genome.jp/kegg-bin/show_pathway?ko04744/K02183%09red
v.genome.jp/kegg-bin/show_pathway?ko04710/K03730%09green
v.genome.jp/kegg-bin/show_pathway?ko04922/K00688%09green/K02183%09red
v.genome.jp/kegg-bin/show_pathway?ko00051/K00011%09green
v.genome.jp/kegg-bin/show_pathway?ko04380/K04694%09green/K06551%09red/K01371%09green
v.genome.jp/kegg-bin/show_pathway?ko00350/K00276%09green
v.genome.jp/kegg-bin/show_pathway?ko04010/K16342%09green/K04358%09red
v.genome.jp/kegg-bin/show_pathway?ko04724/K16342%09green
v.genome.jp/kegg-bin/show_pathway?ko04960/K13302%09green
v.genome.jp/kegg-bin/show_pathway?ko04024/K04145%09green/K04665%09green/K02183%09red
v.genome.jp/kegg-bin/show_pathway?ko00260/K00276%09green
v.genome.jp/kegg-bin/show_pathway?ko05205/K02354%09green/K06106%09green/K00572%09red
v.genome.jp/kegg-bin/show_pathway?ko00380/K00502%09green
v.genome.jp/kegg-bin/show_pathway?ko04670/K05699%09green/K06461%09green
v.genome.jp/kegg-bin/show_pathway?ko04978/K04981%09green
v.genome.jp/kegg-bin/show_pathway?ko04975/K05641%09green
v.genome.jp/kegg-bin/show_pathway?ko04728/K04145%09green/K02183%09red

v.genome.jp/kegg-bin/show_pathway?ko00983/K00699%09red
v.genome.jp/kegg-bin/show_pathway?ko04068/K17845%09green/K13302%09green
v.genome.jp/kegg-bin/show_pathway?ko05340/K07360%09red
v.genome.jp/kegg-bin/show_pathway?ko00480/K18592%09red
v.genome.jp/kegg-bin/show_pathway?ko05030/K04145%09green
v.genome.jp/kegg-bin/show_pathway?ko00600/K12349%09red
v.genome.jp/kegg-bin/show_pathway?ko04550/K02354%09green/K00572%09red
v.genome.jp/kegg-bin/show_pathway?ko04930/K04694%09green
v.genome.jp/kegg-bin/show_pathway?ko04310/K02354%09green/K00572%09red
v.genome.jp/kegg-bin/show_pathway?ko05206/K04694%09green/K06252%09green
v.genome.jp/kegg-bin/show_pathway?ko04210/K07374%09green/K01371%09green
v.genome.jp/kegg-bin/show_pathway?ko05140/K03990%09green/K06461%09green
v.genome.jp/kegg-bin/show_pathway?ko02010/K05641%09green
v.genome.jp/kegg-bin/show_pathway?ko04514/K07378%09green/K16360%09green/K06461%09green
v.genome.jp/kegg-bin/show_pathway?ko05223/K08528%09green
v.genome.jp/kegg-bin/show_pathway?ko04924/K02183%09red
v.genome.jp/kegg-bin/show_pathway?ko05131/K06106%09green
v.genome.jp/kegg-bin/show_pathway?ko04621/K12805%09green
v.genome.jp/kegg-bin/show_pathway?ko05214/K02183%09red
v.genome.jp/kegg-bin/show_pathway?ko04720/K02183%09red
v.genome.jp/kegg-bin/show_pathway?ko05031/K02183%09red
v.genome.jp/kegg-bin/show_pathway?ko04976/K00659%09green
v.genome.jp/kegg-bin/show_pathway?ko05412/K05699%09green
v.genome.jp/kegg-bin/show_pathway?ko04360/K06821%09red/K06521%09green
v.genome.jp/kegg-bin/show_pathway?ko00562/K00913%09red
v.genome.jp/kegg-bin/show_pathway?ko04971/K02183%09red
v.genome.jp/kegg-bin/show_pathway?ko04917/K04694%09green
v.genome.jp/kegg-bin/show_pathway?ko04520/K05699%09green
v.genome.jp/kegg-bin/show_pathway?ko00982/K00699%09red
v.genome.jp/kegg-bin/show_pathway?ko04020/K05849%09green/K02183%09red
v.genome.jp/kegg-bin/show_pathway?ko00230/K01510%09red/K18283%09green
v.genome.jp/kegg-bin/show_pathway?ko05100/K06106%09green
v.genome.jp/kegg-bin/show_pathway?ko00980/K00699%09red
v.genome.jp/kegg-bin/show_pathway?ko04146/K00659%09green
v.genome.jp/kegg-bin/show_pathway?ko04350/K04665%09green
v.genome.jp/kegg-bin/show_pathway?ko04640/K06461%09green
v.genome.jp/kegg-bin/show_pathway?ko05016/K10408%09green
v.genome.jp/kegg-bin/show_pathway?ko04970/K02183%09red
v.genome.jp/kegg-bin/show_pathway?ko05132/K12805%09green
v.genome.jp/kegg-bin/show_pathway?ko04080/K04145%09green/K04241%09red/K05225%09green
v.genome.jp/kegg-bin/show_pathway?ko05145/K04694%09green/K06245%09green
v.genome.jp/kegg-bin/show_pathway?ko04713/K02183%09red
v.genome.jp/kegg-bin/show_pathway?ko05032/K18283%09green
v.genome.jp/kegg-bin/show_pathway?ko04066/K05466%09green
v.genome.jp/kegg-bin/show_pathway?ko00240/K01510%09red

v.genome.jp/kegg-bin/show_pathway?ko04933/K06237%09green
v.genome.jp/kegg-bin/show_pathway?ko04660/K07360%09red
v.genome.jp/kegg-bin/show_pathway?ko05142/K03990%09green
v.genome.jp/kegg-bin/show_pathway?ko04064/K07360%09red
v.genome.jp/kegg-bin/show_pathway?ko04620/K01371%09green
v.genome.jp/kegg-bin/show_pathway?ko04915/K02183%09red
v.genome.jp/kegg-bin/show_pathway?ko04931/K00688%09green
v.genome.jp/kegg-bin/show_pathway?ko04722/K02183%09red
v.genome.jp/kegg-bin/show_pathway?ko04071/K12349%09red
v.genome.jp/kegg-bin/show_pathway?ko04114/K02183%09red
v.genome.jp/kegg-bin/show_pathway?ko04120/K04694%09green
v.genome.jp/kegg-bin/show_pathway?ko04142/K01371%09green
v.genome.jp/kegg-bin/show_pathway?ko05012/K04145%09green
v.genome.jp/kegg-bin/show_pathway?ko04261/K02183%09red
v.genome.jp/kegg-bin/show_pathway?ko04630/K04694%09green
v.genome.jp/kegg-bin/show_pathway?ko05010/K02183%09red
v.genome.jp/kegg-bin/show_pathway?ko05323/K01371%09green
v.genome.jp/kegg-bin/show_pathway?ko05168/K03990%09green/K15411%09green
v.genome.jp/kegg-bin/show_pathway?ko05166/K02354%09green/K00572%09red
v.genome.jp/kegg-bin/show_pathway?ko04740/K05849%09green/K02183%09red/K18283%09green
v.genome.jp/kegg-bin/show_pathway?ko05169/K01510%09red
v.genome.jp/kegg-bin/show_pathway?ko04060/K04665%09green
v.genome.jp/kegg-bin/show_pathway?ko04144/K12489%09green
v.genome.jp/kegg-bin/show_pathway?ko04650/K07360%09red

/K03903%09green/K03902%09green/K06461%09green
11252%09green/K11253%09green
145%09green/K05466%09green/K02183%09red

n/K11252%09green/K11253%09green
466%09green/K07360%09red
/K06237%09green

n/K11252%09green

06245%09green/K05466%09green/K06237%09green

/K06461%09green

/K07374%09green/K07375%09green/K06560%09green

06245%09green/K00572%09red/K06237%09green

/K06237%09green

/K02183%09red

Supplementary Material 5

Supplementary Table 3. Information of clinical samples

	Control	Intestinal obstruction caused by carcinoma of colon
Age (years)	53±6	53±6
Gender (M/F)	2/1	2/1
Time of biopsy	2019.6.1-2019.12.31	
