# Supplementary material for: HRD1-induced TMEM2 ubiquitination promotes ER stress-mediated apoptosis through a non-canonical pathway in intestinal ischemia/reperfusion
Source: Cell Death Dis. 2024 Feb 20;15(2):154. doi: 10.1038/s41419-024-06504-0 (PMC10879504; doi:10.1038/s41419-024-06504-0)

Figure 1D

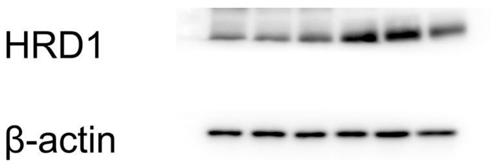

Figure 1H

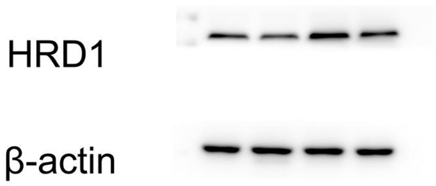

Figure 1J

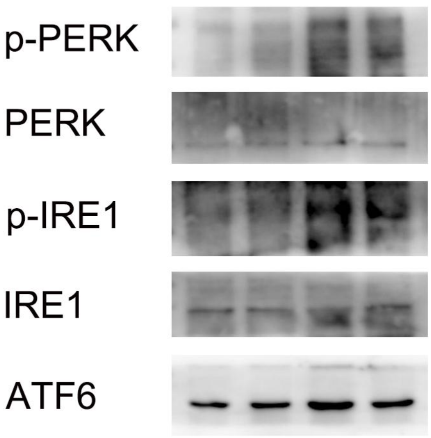

Figure 1L

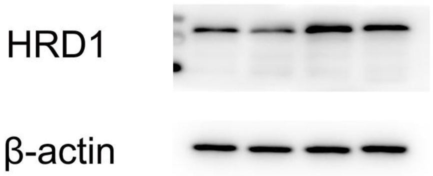

Figure 2D

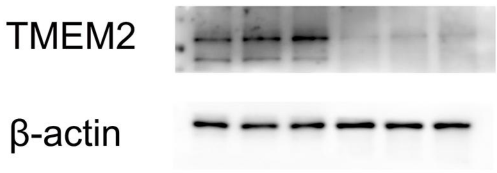

Figure 1E

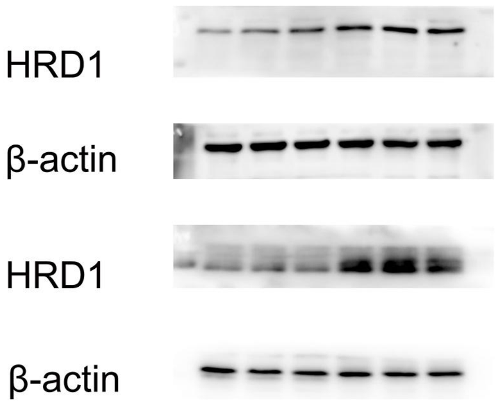

Figure 1J

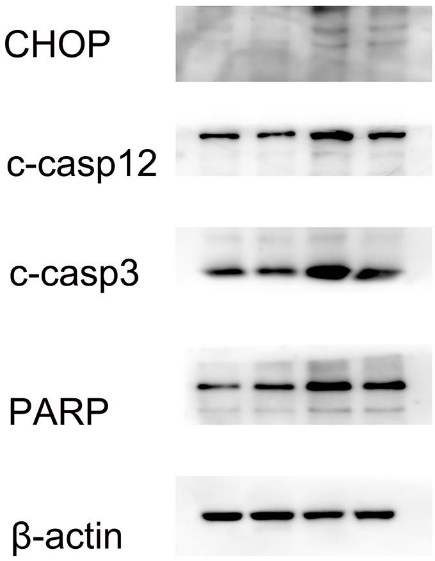

Figure 2C

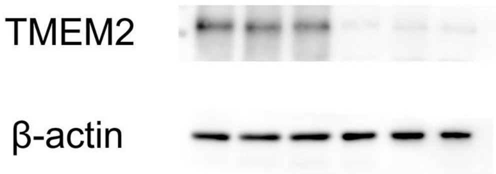

Figure 2F

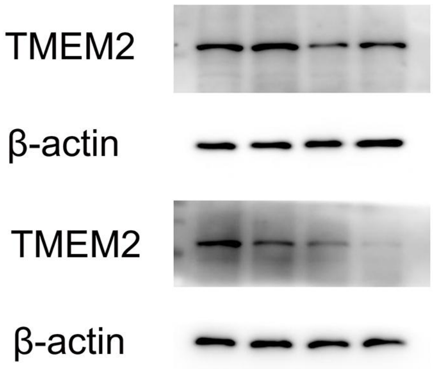

Figure 1G

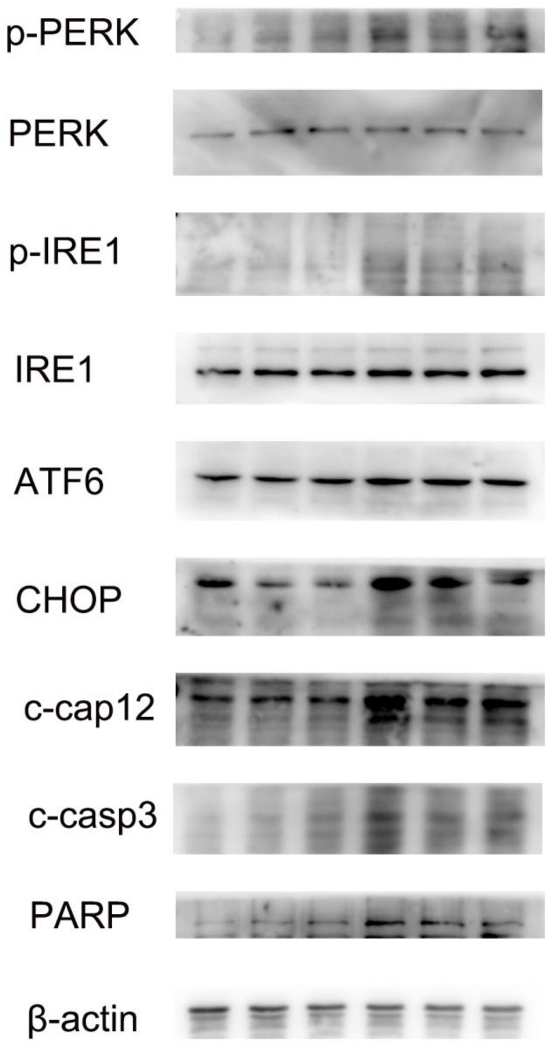

Figure 2A

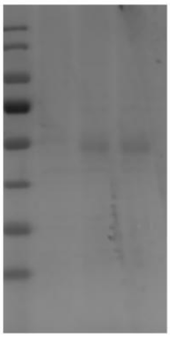

Figure 2G

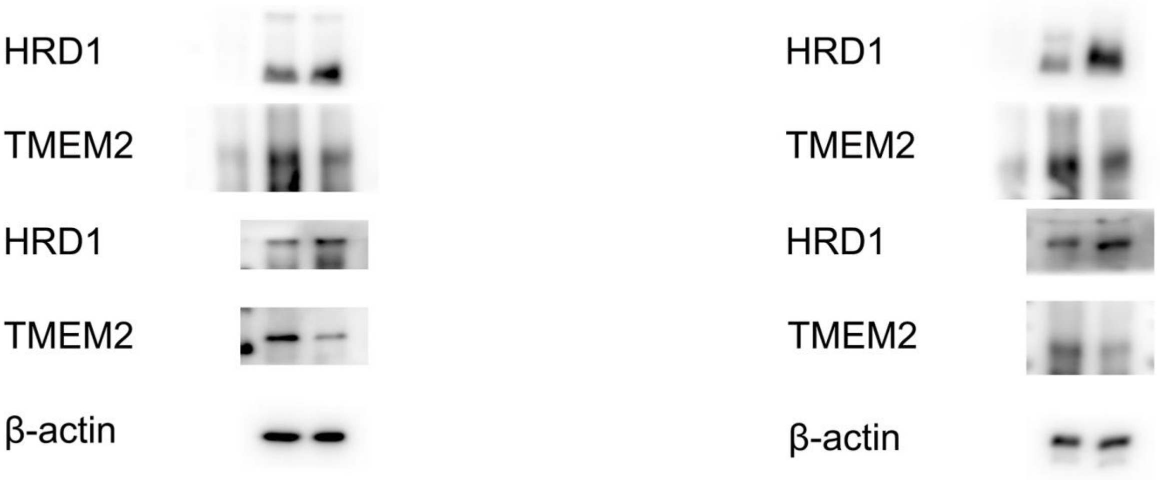

Figure 2H

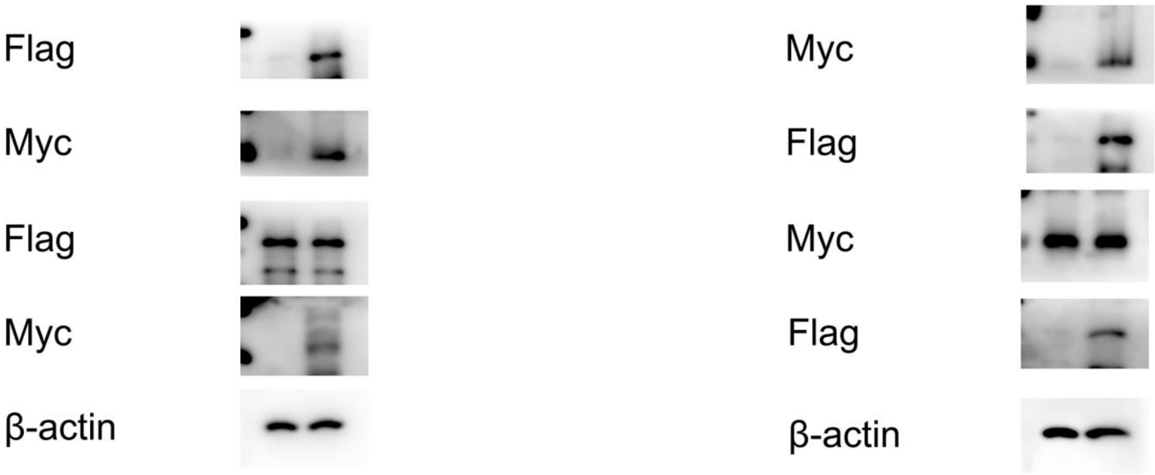

Figure 2I

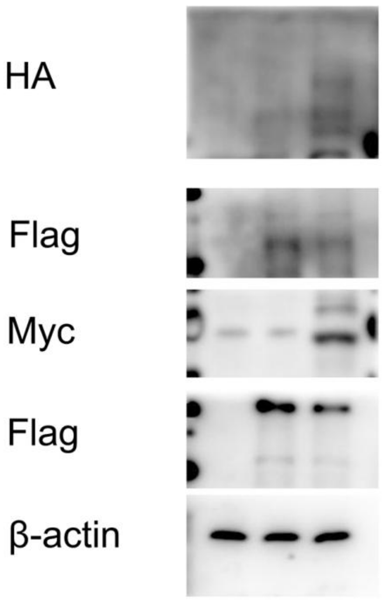

Figure 2J

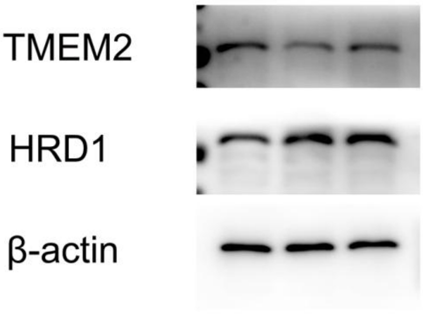

Figure 2K

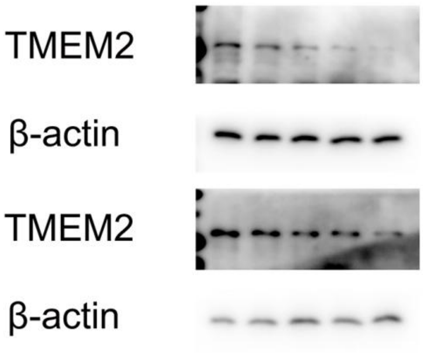

Figure 3A

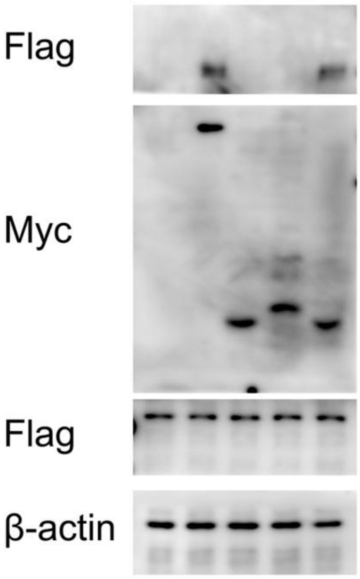

Figure 3B

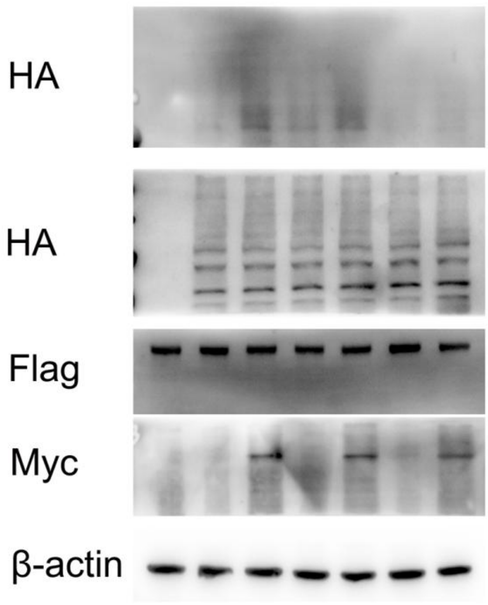

Figure 3C

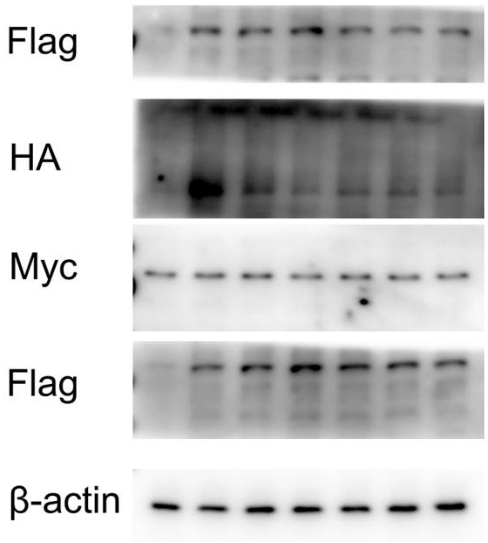

Figure 3D

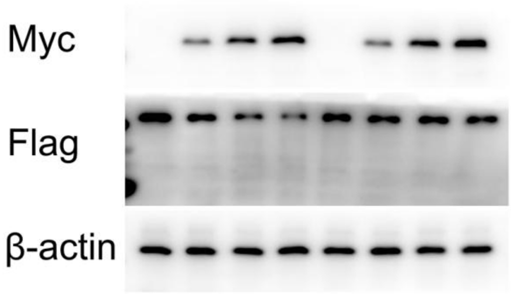

Figure 3E

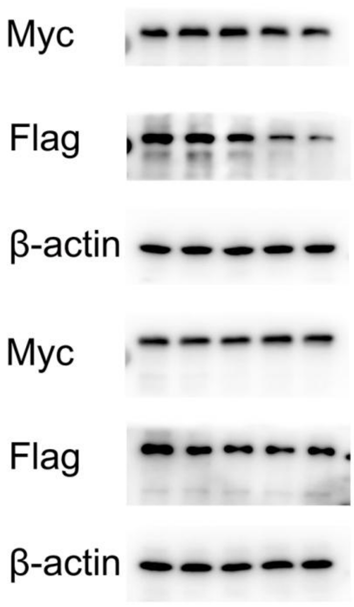

Figure 4A

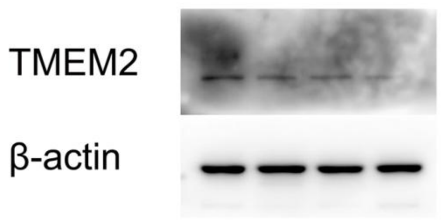

Figure 4E

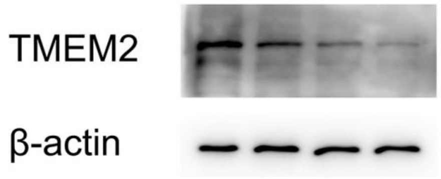

Figure 5E

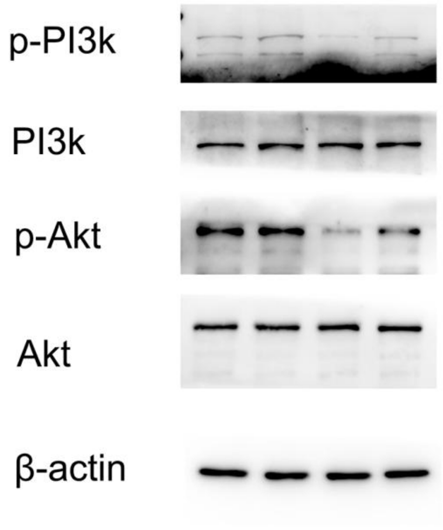

Figure 5H

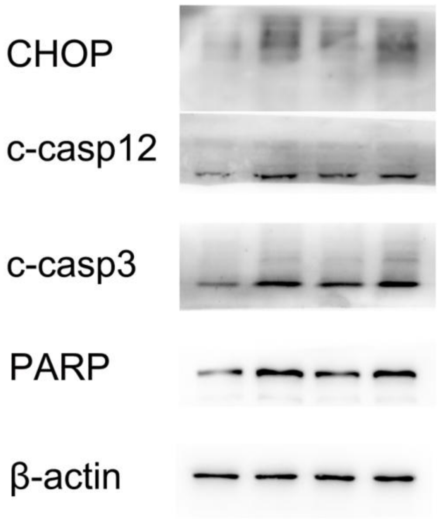

Figure 4C

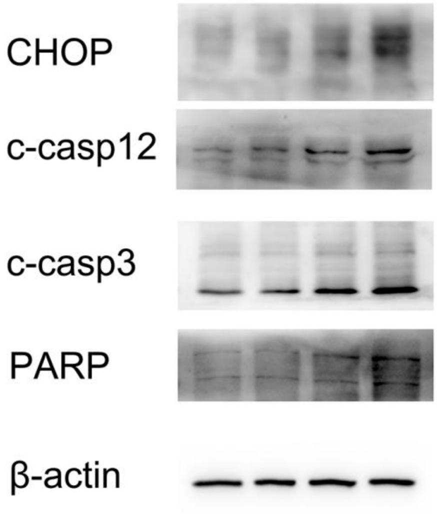

Figure 5F

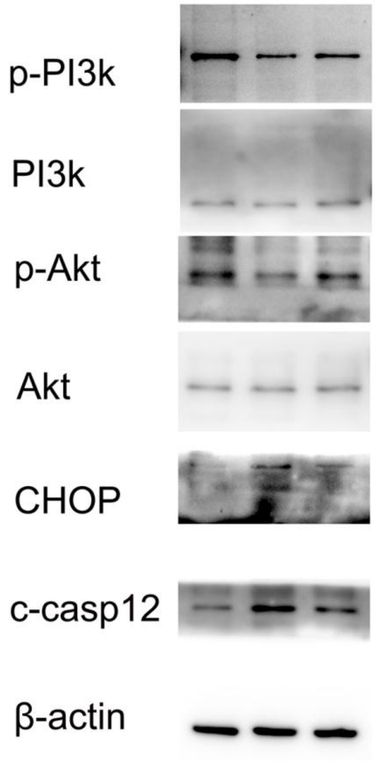

Figure 6B

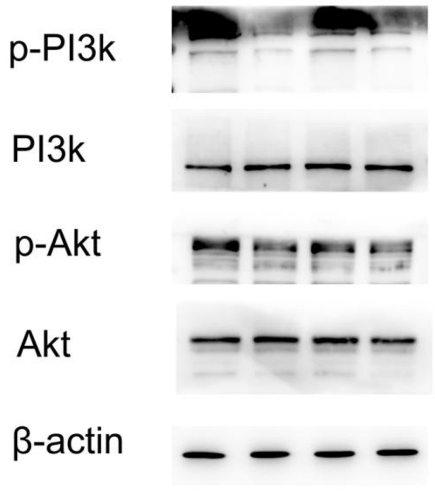

Figure 6C

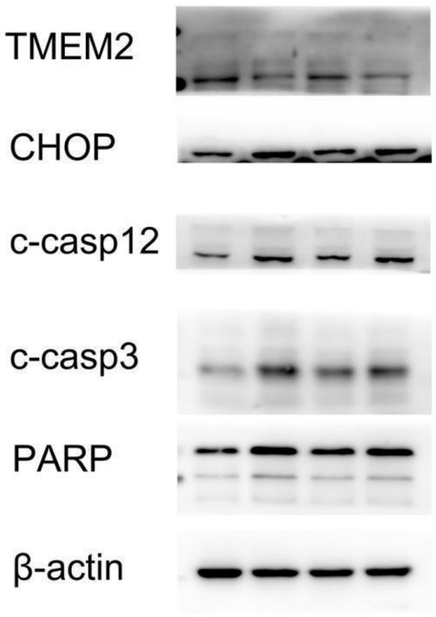

Figure S3A

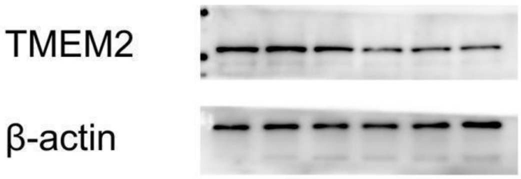

Figure S2B

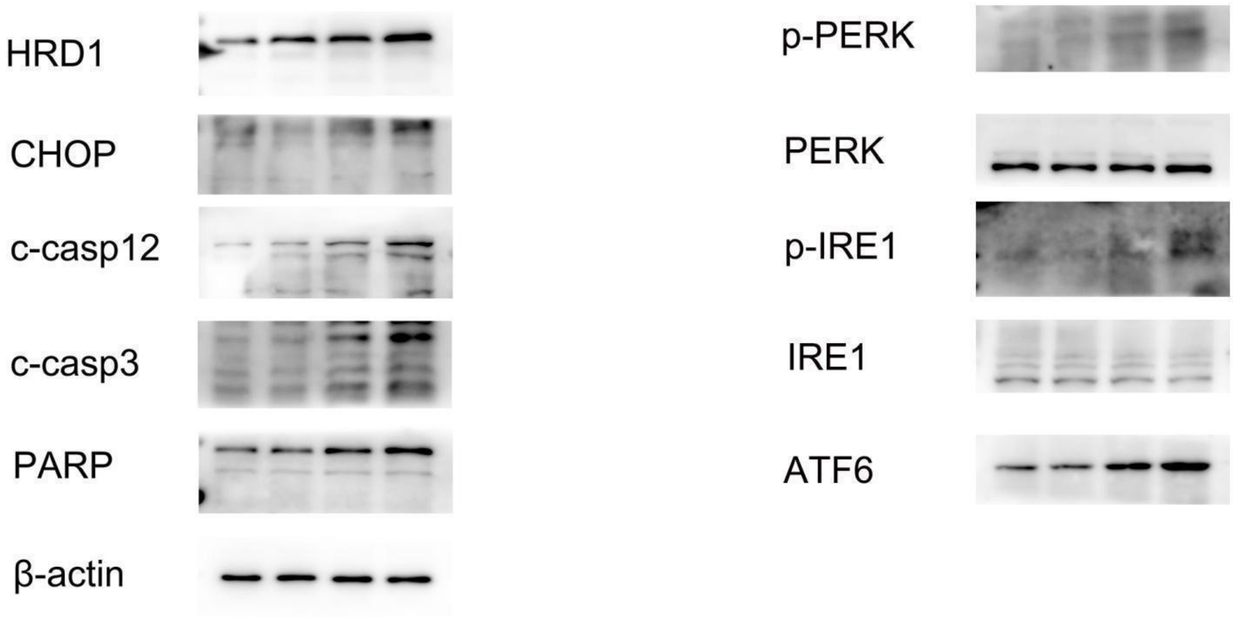

Figure S5A

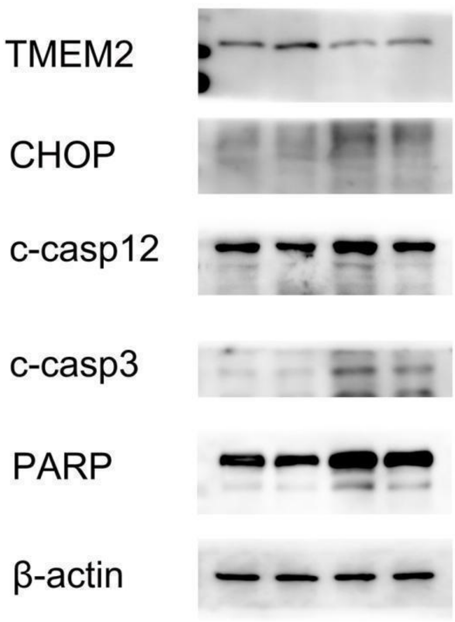

Figure S4A

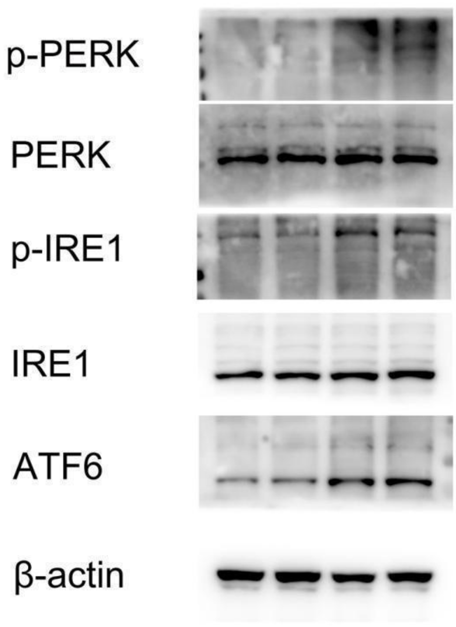

Supplement: Supplementary file 2 — Original western blots [file 41419_2024_6504_MOESM2_ESM.pdf]
